# Effect of Ecdysterone on the Hepatic Transcriptome and Lipid Metabolism in Lean and Obese Zucker Rats

**DOI:** 10.3390/ijms22105241

**Published:** 2021-05-15

**Authors:** Magdalena J. M. Marschall, Robert Ringseis, Denise K. Gessner, Sarah M. Grundmann, Erika Most, Gaiping Wen, Garima Maheshwari, Holger Zorn, Klaus Eder

**Affiliations:** 1 Institute of Animal Nutrition and Nutrition Physiology, Justus-Liebig-University Giessen, Heinrich-Buff-Ring 26-32, 35392 Giessen, Germany; Magdalena.Marschall@ernaehrung.uni-giessen.de (M.J.M.M.); Denise.Gessner@ernaehrung.uni-giessen.de (D.K.G.); Sarah.Grundmann@ernaehrung.uni-giessen.de (S.M.G.); erika.most@ernaehrung.uni-giessen.de (E.M.); Gaiping.wen@ernaehrung.uni-giessen.de (G.W.); maheshwari@uni-bonn.de (G.M.); Klaus.Eder@ernaehrung.uni-giessen.de (K.E.); 2 Institute of Food Chemistry and Food Biotechnology, Justus-Liebig-University Giessen, Heinrich-Buff-Ring 17, 35392 Giessen, Germany; holger.zorn@lcb.chemie.uni-giessen.de; 3 Fraunhofer Institute for Molecular Biology and Applied Ecology, Ohlebergsweg 12, 35392 Giessen, Germany

**Keywords:** ecdysterone, 20-hydroxyecdysone, lipid metabolism, liver, triglyceride, cholesterol, obese Zucker rat, obesity, transcriptome

## Abstract

Conflicting reports exist with regard to the effect of ecdysterone, the predominating representative of steroid hormones in insects and plants, on hepatic and plasma lipid concentrations in different rodent models of obesity, fatty liver, and diabetes, indicating that the effect is dependent on the rodent model used. Here, the hypothesis was tested for the first time that ecdysterone causes lipid-lowering effects in genetically obese Zucker rats. To test this hypothesis, two groups of male obese Zucker rats (*n* = 8) were fed a nutrient-adequate diet supplemented without or with 0.5 g ecdysterone per kg diet. To study further if ecdysterone is capable of alleviating the strong lipid-synthetic activity in the liver of obese Zucker rats, the study included also two groups of male lean Zucker rats (*n* = 8) which also received either the ecdysterone-supplemented or the non-supplemented diet. While hepatic and plasma concentrations of triglycerides and cholesterol were markedly higher in the obese compared to the lean rats (*p* < 0.05), hepatic and plasma triglyceride and cholesterol concentrations did not differ between rats of the same genotype fed the diets without or with ecdysterone. In conclusion, the present study clearly shows that ecdysterone supplementation does not exhibit lipid-lowering actions in the liver and plasma of lean and obese Zucker rats.

## 1. Introduction

Ecdysteroids are a class of steroid hormones occurring in insects, where they are referred to as zooecdysteroids, and in plants, where they are termed as phytoecdysteroids. While zooecdysteroids in insects regulate important developmental processes, such as embryogenesis, moulting (ecdysis), metamorphosis, reproduction, and diapause [1], phytoecdysteroids provide protection against invertebrate predators by acting as feeding deterrents and by disrupting critical developmental processes of such invertebrates [2]. The ecdysteroids in insects and plants comprise a great number of different analogues, with 20-hydroxyecdysone, also called ecdysterone, being the quantitatively dominating biologically active analogue in both insects and plants. Owing to the role of ecdysteroids in insects’ development and their importance as moulting hormones, the haemolymph concentration of ecdysterone is largely dependent on the insect´s developmental stage, with ecdysteroid levels being low through the intermoult but showing a major peak during each moult in the cuticle induction phase [3,4]. In plants, evidence has been gained that all species have the capacity to produce at least low levels of phytoecdysteroids [5], but their concentration varies according to species, ecotype, developmental stage, plant part, and presence/absence of stress conditions. Very high levels of ecdysterone have been documented for roots and stems, respectively, of medicinal plants, such as *Cyanotis arachnoidea* [6] and *Diploclisia glaucescens* [7], but also food plants, such as spinach (*Spinacia oleracea*) and quinoa (*Chenopodium quinoa*), were reported to contain noticeable levels of phytoecdysteroids [8], whose levels are strongly inducible by mechanical or insect damage of the root [9,10].

Although phytoecdysteroids are effective toxins or antifeedants towards non-adapted herbivorous invertebrate predators, ecdysteroids are apparently non-toxic to mammals and even have been shown to exert a variety of interesting metabolic actions, such as antiobesogenic, hypoglycaemic, and protein anabolic effects [11,12,13]. In addition, ecdysterone was reported to decrease liver and plasma triglyceride and cholesterol concentrations in streptozotocin-induced steatotic male Wistar rats [14]. In contrast, no effect of different ecdysterone doses on serum triglyceride and cholesterol concentrations was found in female ovariectomised Sprague Dawley rats fed a high-fat/high-fructose diet [15]. Moreover, in two studies with male C57BL/6J mice fed with a high-fat diet supplemented with ecdysterone, no alterations were observed in plasma and/or liver triglyceride and cholesterol concentrations compared to the non-supplemented high-fat diet [12,16]. Since the ecdysterone doses administered orally via the diet or gavage were comparable between these rodent studies [12,14,15,16], the inconsistent outcomes with regard to the effects of ecdysterone on hepatic and plasma lipid concentrations indicate that the effect is dependent on the rodent model used. Besides the abovementioned inducible rodent models of obesity, fatty liver, and diabetes, the obese Zucker rat is a genetic rodent model for obesity, fatty liver, and hyperlipidaemia, which has not yet been used to investigate the effects of ecdysterone.

In view of this, the present study aimed to test the hypothesis that ecdysterone causes lipid-lowering effects in obese Zucker rats. To test this hypothesis, two groups of obese Zucker rats were fed a nutrient-adequate diet supplemented without (group OC) or with (group OE) 0.5 g ecdysterone per kg diet. This ecdysterone concentration was appropriate to achieve a similar dose of ecdysterone per kg body weight as applied in other rodent studies, in which ecdysterone caused either hepatic and plasma lipid-lowering effects or antiobesity effects in different rodent models [14,15,16]. In order to decipher the potential lipid-lowering actions of ecdysteroids in Zucker rats, measurements of liver and plasma lipid concentrations and hepatic transcriptome analysis were carried out. To study further if ecdysterone is capable of alleviating the strong lipid-synthetic activity in the liver of obese Zucker rats, the study included also two groups of lean Zucker rats which also received either the ecdysterone-supplemented (group LE) or the non-supplemented diet (group LC).

## 2. Results

### 2.1. Growth Performance and Organ Weights

Initial and final body weights, body weight gain, feed intake, feed:gain ratio, and organ weights of the rats were only influenced by the genotype but not by ecdysterone (Table 1). Obese rats had higher initial and final body weights, body weight gain, feed intake, weights of liver, heart and kidneys, and lower weights of selected muscles than lean rats (*p* < 0.05). No interactions between ecdysterone and genotype were observed with regard to these parameters.

### 2.2. Hepatic and Plasma Lipid Concentrations

**Figure 1:**
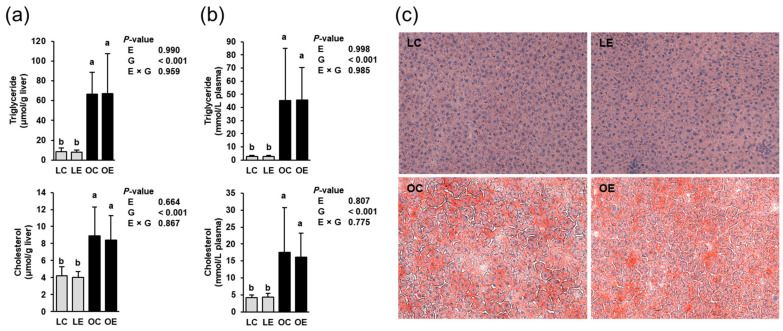
Liver (**a**) and plasma (**b**) triglycerides and cholesterol concentrations of lean and obese Zucker rats fed a semisynthetic diet without or with 0.5 g ecdysterone per kg diet for four weeks. Data are means ± SD; *n* = 8 rats per group. Means not sharing the same letters (^a,b^) differ (*p* < 0.05). Oil Red O-stained liver sections (**c**) of lean and obese Zucker rats fed a semisynthetic diet without or with 0.5 g ecdysterone per kg diet for four weeks. Images are shown for one animal per group (magnification × 20). Abbreviations: E, ecdysterone; G, genotype; LC, lean rats fed without ecdysterone; LE, lean rats fed with ecdysterone; OC, obese rats fed without ecdysterone; OE, obese rats fed with ecdysterone.

Liver and plasma triglyceride and cholesterol concentrations of the rats were influenced by the genotype but not by ecdysterone (Figure 1a); the obese rats had higher concentrations of triglycerides and cholesterol in liver and plasma than the lean rats (*p* < 0.05). There was no interaction between ecdysterone and genotype with regard to the liver and plasma triglyceride and cholesterol concentrations. In agreement with the quantitative measurement of hepatic lipid concentrations, the Oil Red O-stained liver sections of the two lean groups (LC, LE) showed a normal appearance of the parenchyma structure with normal liver cell morphology, clear edges, clearly visible haematoxylin-stained nuclei, and no abnormalities (Figure 1b). In contrast, the Oil Red O-stained liver sections of the obese groups (OC, OE) exhibited a pathological parenchyma structure with enlarged liver cells and a marked accumulation of lipids. No difference was observed between lean rats fed with (LE) or without ecdysterone (LC) and between obese rats fed with (OE) or without ecdysterone (OC).

In line with the hepatic triglyceride concentrations, concentrations of fatty acids of hepatic total lipids were mainly affected by the genotype (Table 2). Hepatic concentrations of most individual fatty acids (14:0, 14:1 n-5, 16:0, 16:1 n-7, 18:0, 18:1 n-9, 18:3 n-3, 18:3 n-6, 20:3 n-6) and the sum of all individual fatty acids were higher and those of 20:4 n-6 and 22:6 n-3 were lower in obese rats than in lean rats. Only the concentration of 22:5 n-3 was affected by ecdysterone; rats fed ecdysterone had lower concentrations of 22:5 n-3 than rats fed without.

### 2.3. Genotype- and Ecdysterone-Regulated Transcripts in the Liver

**Figure 2:**
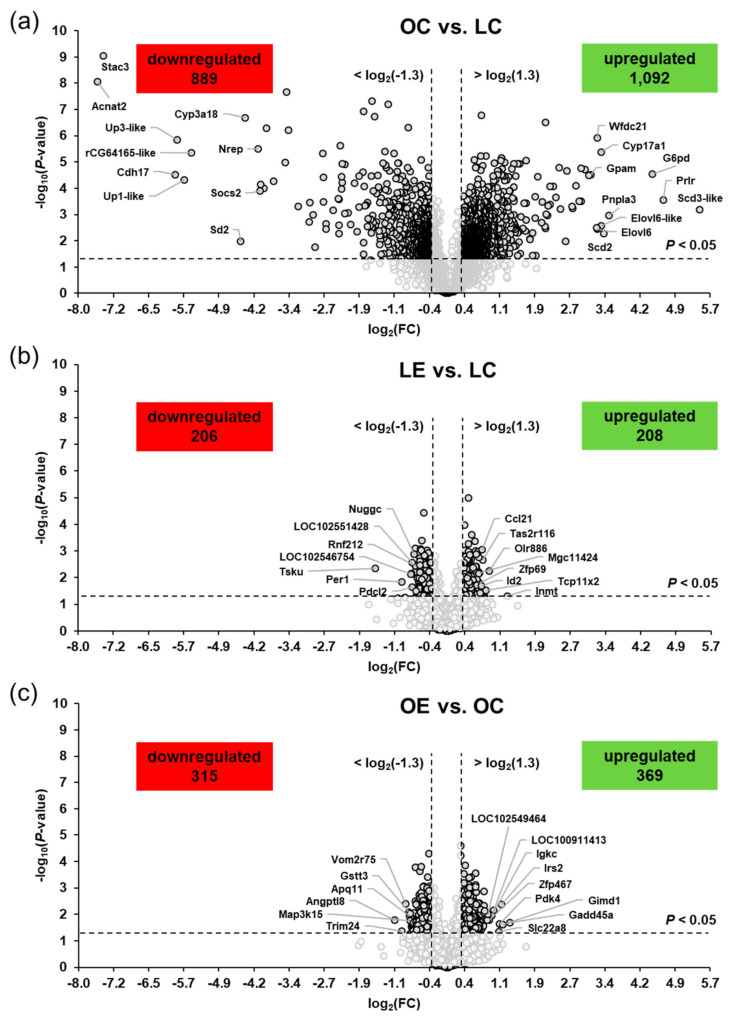
Volcano plots showing the differentially regulated hepatic transcripts between the groups OC vs. LC (**a**), groups LE vs. LC (**b**), and groups OE vs. OC (**c**). The double filtering criteria are indicated by horizontal (*p*-value < 0.05) and vertical (FC: > log_2_(1.3) or <log_2_(−1.3)) dashed lines. Transcripts in the upper left and the upper right corner represent the downregulated and the upregulated transcripts, respectively. Abbreviations: LC, lean rats fed without ecdysterone; LE, lean rats fed with ecdysterone; OC, obese rats fed without ecdysterone; OE, obese rats fed with ecdysterone.

Differential transcript profiling was carried out to identify the genotype- and ecdysterone-induced changes in hepatic gene expression of the rats. As expected, profound changes in hepatic gene expression were induced by the genotype. When comparing the obese and the lean groups of rats fed the basal diet without ecdysterone (OC vs. LC), a total of 1981 hepatic transcripts were differentially regulated according to the two-filter criteria (FC > 1.3 or < −1.3, *p* < 0.05); of those, 1092 transcripts were upregulated and 889 transcripts were downregulated in the OC group, compared to the LC group (Figure 2a). Amongst the upregulated transcripts, 172 transcripts were regulated >2.0-fold and the 10 most strongly upregulated transcript were in decreasing order of their FCs (in brackets) as follows: *Sdc3-like* (44.5), *Prlr* (25.7), *G6pd* (21.8), *Pnpla3* (11.4), *Scd2* (10.5), *Cyp17a1* (10.2), *Elovl6-like* (10.1), *Wfdc21* (9.6), *Elovl6* (9.5), and *Gpam* (8.6). A total of 162 transcripts were downregulated < −2.0-fold, with the 10 most strongly downregulated transcripts being in increasing order of their FCs (in brackets) as follows: *Acnat2* (−190), *Stac3* (−173), *Cdh17* (−59.1), *Up3-like* (−57.6), *Up1-like* (−51.4), *rCG64165-like* (−46.1), *Sds* (−22.1), *Cyp3a18* (−20.7), *Nrep* (−17.0), and *Socs2* (-16.6). The FC and *p*-value of all differentially expressed transcripts between groups OC vs. LC are listed in Supplementary materials: Table S1.

In comparison to the profound genotype effect on hepatic gene expression, the effect of ecdysterone on hepatic transcriptome was markedly weaker. Based on the same filter criteria as applied for studying the genotype effect, a total of 414 transcripts were identified as differentially expressed (upregulated: 208, downregulated: 206) between the ecdysterone-supplemented and the non-supplemented lean groups (LE vs. LC) (Figure 2b). Amongst the upregulated genes, only one gene (*Inmt*) was regulated >2.0-fold. The top 10 upregulated transcripts were in decreasing order of their FCs (in brackets) as follows: *Inmt* (2.47), *Olr886* (1.87), *Tcp11x2* (1.79), *Tas2r116* (1.70), *Ccl21* (1.69), *Id2* (1.67), *Zfp69* (1.63), *Olr377* (1.62), *MGC114246* (1.62) and *Lpcat2* (1.60). Additionally, only one gene (*Tsku*) amongst the downregulated genes exhibited a regulation < −2.0-fold. The top 10 downregulated transcripts were in increasing order of their FCs (in brackets) as follows: *Tsku* (−2.95), *Per1* (−1.98), *LOC102546754* (−1.72), *Mansc1* (−1.71), *Pdcl2* (−1.69), *LOC102551428* (−1.69), *Nuggc* (−1.64), *RGD1561778* (−1.63), *Mgat4e* (−1.62) and *Rnf212* (−1.61). The FC and *p*-value of all differentially expressed transcripts between groups LE vs. LC are shown in Supplementary materials: Table S2.

When studying the ecdysterone effect within the obese rats, a total of 684 transcripts were identified as differentially expressed (upregulated: 369, downregulated: 315) between the ecdysterone-supplemented and the non-supplemented groups (OE vs. OC) (Figure 2c). Amongst the upregulated genes, only six genes were regulated >2.0-fold and the strongest ecdysterone-induced gene was *Gadd45a* (2.57). The top 10 upregulated transcripts were in decreasing order of their FCs (in brackets) as follows: *Gadd45a* (2.57), *Gimd1* (2.32), *Irs2* (2.27), *Pdk4* (2.19), *Slc22a8* (2.17), *LOC100911413* (2.01), *Igkc* (1.97), *Zfp467* (1.88), *LOC102549464* (1.86), and *Junb* (1.83). Only one gene (*Angptl8*) exhibited a regulation <−2.0-fold. The top 10 downregulated transcripts were in increasing order of their FCs (in brackets) as follows: *Angptl8* (−2.19), *Trim24* (−1.98), *Vom2r75* (−1.86), *Ces4a* (−1.78), *Aqp11* (−1.76), *Gstt3* (−1.74), *Map3k15* (−1.73), *Enpp5* (−1.72), *Hebp2* (−1.71), and *RGD1559459* (−1.69). The FC and *p*-value of all differentially expressed transcripts between groups OE vs. OC are displayed in Supplementary materials: Table S3.

### 2.4. Technical Validation of Microarray Data

Microarray data of 19 differentially expressed transcripts between groups OC and LC were validated by qPCR. As shown in Supplementary materials: Table S4, the effect direction (positive or negative FC) was the same between microarray and qPCR for all validated transcripts, whereas the effect size (value of FC) differed to some extent for the validated transcripts between microarray and qPCR. Statistical analysis of qPCR data revealed that 17 (*G6pd*, *Scd2*, *Elovl6*, *Gpam*, *Cd36*, *Me1*, *Fasn*, *Fads2*, *Elovl5*, *Car3*, *Dhrs7, Sult1c3*, *Nrep*, *Cyp3a18*, *Sds*, *Cdh17*, *Acnat2*) of the validated transcripts were regulated significantly (*p* < 0.05), whereas two (*Srebf1*, *Ldlr*) of the transcripts were not regulated (*p* > 0.05).

### 2.5. Biological Processes and Pathways Affected by the Genotype- and Ecdysterone-Regulated Transcripts in the Liver

**Figure 3:**
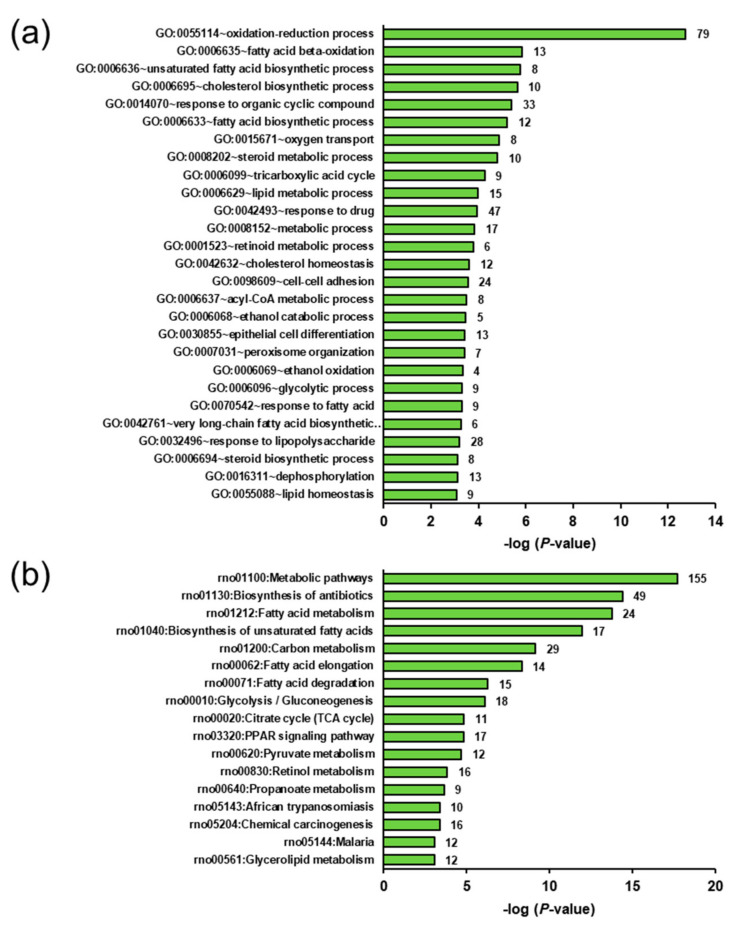
Enriched gene ontology (GO) biological process terms (**a**) and KEGG pathways (**b**) assigned to the genes upregulated in the liver of group OC (obese rats fed without ecdysterone) vs. LC (lean rats fed without ecdysterone). GO terms and KEGG pathways are sorted by their enrichment *p*-values (EASE score) (top: lowest *p*-value, bottom: highest *p*-value). Only GO terms and KEGG pathways with *p*-values < 0.001 and *p*-values < 0.01, respectively are shown. The number of genes is shown next to the bars.

To identify biological processes and pathways affected by the genotype- and ecdysterone-regulated transcripts, GSEA was performed using GO biological process terms and/or KEGG pathways, respectively. With regard to genotype effect, GSEA of the transcripts upregulated in group OC vs. LC revealed that several of the most enriched biological process terms were related to lipid synthesis, such as unsaturated fatty acid biosynthetic process, cholesterol biosynthetic process, fatty acid biosynthetic process, steroid metabolic process, and lipid metabolic process (Figure 3a). The most enriched KEGG pathways assigned to the transcripts upregulated in group OC vs. LC predominantly comprised lipid metabolic pathways, such as fatty acid metabolism, biosynthesis of unsaturated fatty acids, fatty acid elongation, fatty acid degradation, and PPAR signalling pathway (Figure 3b).

**Figure 4:**
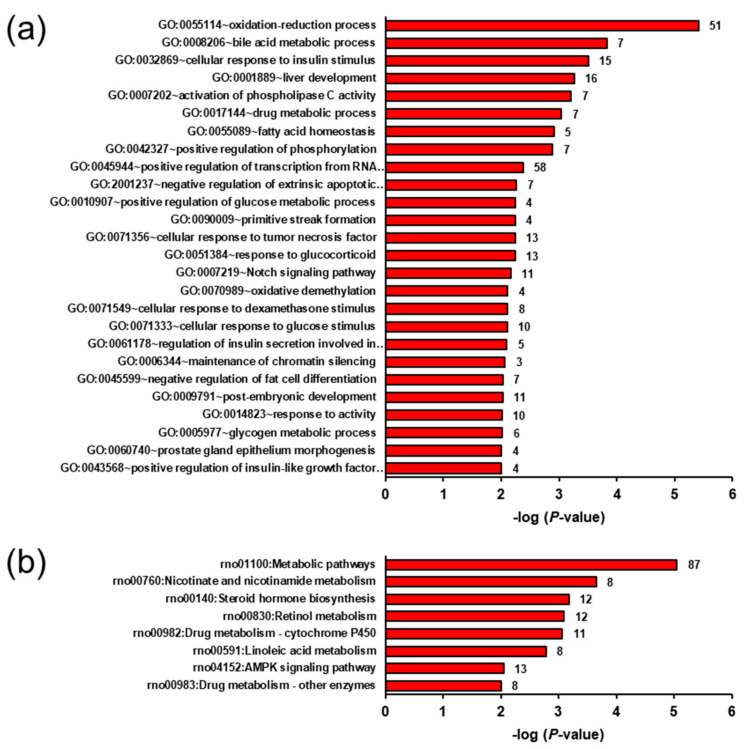
Enriched gene ontology (GO) biological process terms (**a**) and KEGG pathways (**b**) assigned to the genes downregulated in the liver of group OC (obese rats fed without ecdysterone) vs. LC (lean rats fed without ecdysterone). GO terms and KEGG pathways are sorted by their enrichment *p*-values (EASE score) (top: lowest *p*-value, bottom: highest *p*-value). Only GO terms and KEGG pathways with *p*-values < 0.001 and *p*-values < 0.01, respectively are shown. The number of genes is shown next to the bars.

The most enriched biological process terms assigned to the transcripts downregulated in group OC vs. LC included heterogeneous terms, such as oxidation-reduction process, bile acid metabolic process, cellular response to insulin stimulus, liver development, activation of phospholipase C activity, and drug metabolic process (Figure 4a). The most enriched KEGG pathways assigned to these downregulated transcripts comprised amongst others metabolic pathways, nicotinate and nicotinamide metabolism, steroid hormone biosynthesis, retinol metabolism, drug metabolism—cytochrome P450, linoleic acid metabolism, and AMPK signalling pathway (Figure 4b).

**Figure 5:**
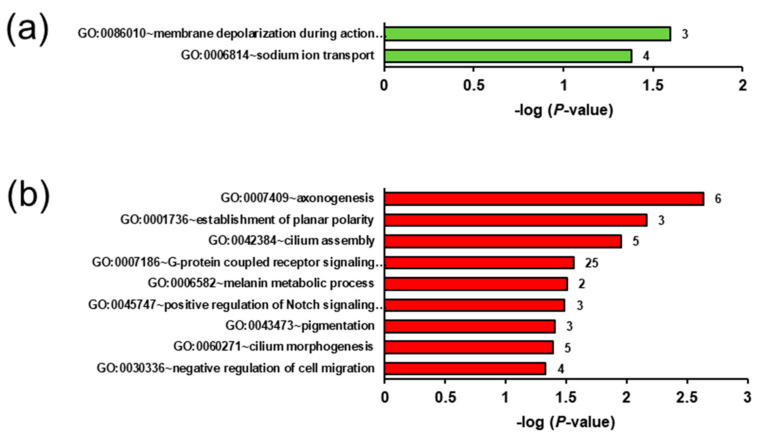
Enriched gene ontology (GO) biological process terms assigned to the genes upregulated (**a**) and downregulated (**b**) in the liver of group LE (lean rats fed with ecdysterone) vs. LC (lean rats fed without ecdysterone). GO terms are sorted by their enrichment *p*-values (EASE score) (top: lowest *p*-value, bottom: highest *p*-value). All GO terms with *p*-values < 0.05 are shown. The number of genes is shown next to the bars.

Regarding the ecdysterone effect in the lean groups, GSEA of the transcripts upregulated in group LE vs. LC revealed only two enriched (*p* < 0.05) biological process terms, namely, membrane depolarisation during action potential and sodium ion transport (Figure 5a), and only one enriched (*p* < 0.05) KEGG pathway, i.e., carbohydrate digestion and absorption. The enriched biological process terms assigned to the transcripts downregulated in group LE vs. LC were axonogenesis, the establishment of planar polarity, cilium assembly, G-protein coupled receptor signalling pathway, melanin metabolic process, positive regulation of Notch signalling pathway, pigmentation, cilium morphogenesis, and negative regulation of cell migration (Figure 5b). No significantly enriched KEGG pathways were assigned to these downregulated transcripts.

**Figure 6:**
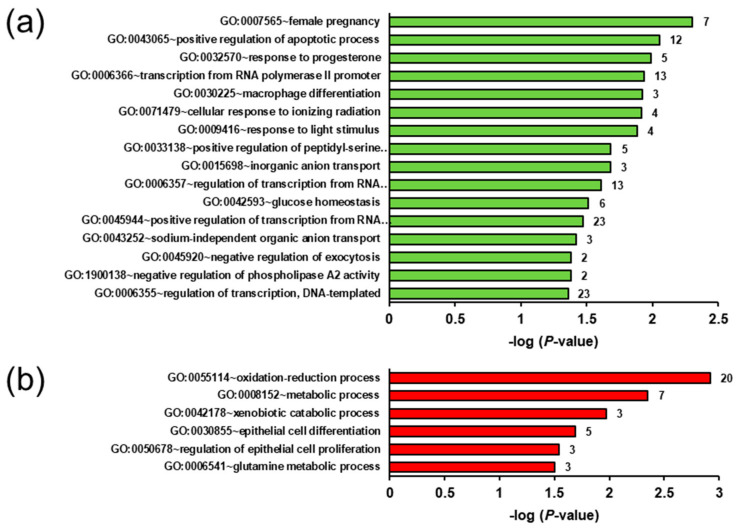
Enriched gene ontology (GO) biological process terms (**a**,**b**) assigned to the genes upregulated (**a**) and downregulated (**b**) in the liver of group OE (obese rats fed with ecdysterone) vs. OC (obese rats fed without ecdysterone). GO terms are sorted by their enrichment *p*-values (EASE score) (top: lowest *p*-value, bottom: highest *p*-value). All GO terms with *p*-values < 0.05 are shown. The number of genes is shown next to the bars.

Investigating the ecdysterone effect in the obese groups, GSEA of the transcripts upregulated in group OE vs. OC showed that female pregnancy, positive regulation of the apoptotic process, response to progesterone, transcription from RNA polymerase II promoter, macrophage differentiation, cellular response to ionizing radiation, response to light stimulus, positive regulation of peptidyl-serine phosphorylation and inorganic anion transport are the most enriched biological process terms assigned to the upregulated transcripts (Figure 6a). Only two enriched (*p* < 0.05) KEGG pathways, i.e., osteoclast differentiation and taste transduction, were assigned to these upregulated transcripts. Only six enriched (*p* < 0.05) biological process terms (oxidation-reduction process, metabolic process, xenobiotic catabolic process, epithelial cell differentiation, regulation of epithelial cell proliferation, glutamine metabolic process) (Figure 6b), and five enriched (*p* < 0.05) KEGG pathways, i.e., metabolic pathways, glutathione metabolism, metabolism of xenobiotics by cytochrome P450, drug metabolism-cytochrome P450 and amoebiasis, were identified within the set of transcripts downregulated in group OE vs. OC.

### 2.6. Effect of Ecdysterone on Genes Involved in Lipid Synthetic Pathways in the Liver

In order to further investigate the potential of ecdysterone in regulating the expression of hepatic lipid synthesizing genes, we filtered all hepatic genes involved in fatty acid, triglyceride, cholesterol, and phospholipid synthesis from microarray data that were upregulated >1.5-fold in obese rats, compared with lean rats (OC vs. LC) (Table 3). Out of 32 filtered genes, that were upregulated 1.56- to 44.5-fold in group OC compared with group LC, only one gene (*Fasn*) was slightly downregulated (−1.39-fold) in the liver of obese rats fed ecdysterone compared with obese rats fed without ecdysterone (OE vs. OC).

### 2.7. Plasma Concentration of Fructosamine

**Figure 7:**
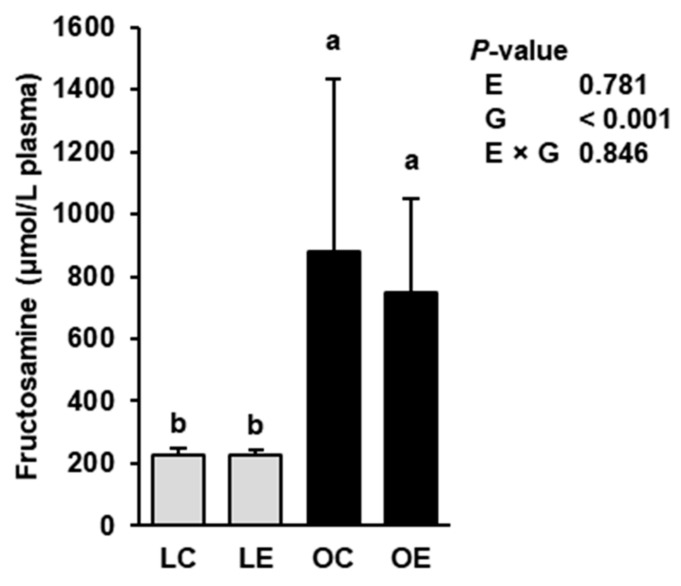
Plasma fructosamine concentration of lean and obese Zucker rats fed a semisynthetic diet without or with 0.5 g ecdysterone per kg diet for four weeks. Data are means ± SD; *n* = 8 rats per group. Means not sharing the same letters (^a,b^) differ (*p* < 0.05). Abbreviations: E, ecdysterone; G, genotype; LC, lean rats fed without ecdysterone; LE, lean rats fed with ecdysterone; OC, obese rats fed without ecdysterone; OE, obese rats fed with ecdysterone.

To clarify if ecdysterone had an effect on glucose metabolism and insulin sensitivity in the Zucker rat model, the plasma concentration of fructosamine, which is a time-averaged indicator of blood glucose levels used to assess the glycaemic status of diabetic subjects, was determined. Plasma concentration of fructosamine was influenced by the genotype but not by ecdysterone (Figure 7); the obese rats had a markedly higher plasma concentration of fructosamine than the lean rats (*p* < 0.05). There was no interaction between ecdysterone and genotype with regard to this parameter.

### 2.8. Histological Analysis of M. rectus Femoris

**Figure 8:**
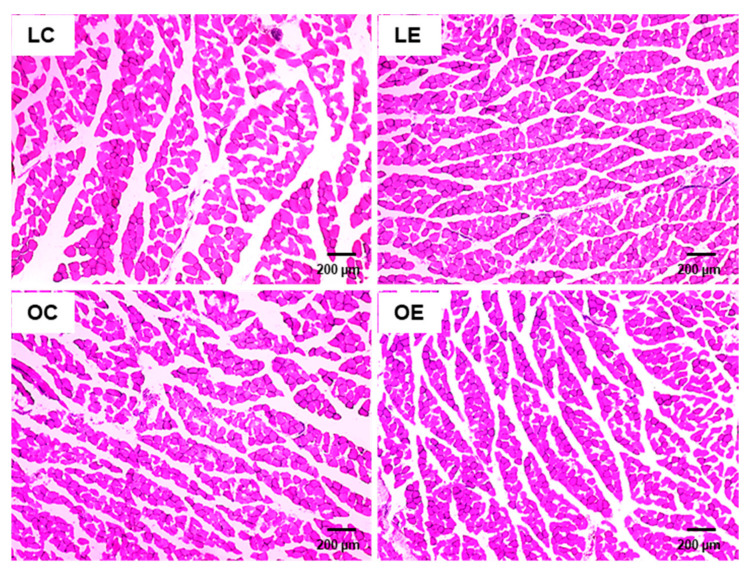
Haematoxylin- and eosin-stained *M. gastrocnemius* sections of lean and obese Zucker rats fed a semisynthetic diet without or with 0.5 g ecdysterone per kg diet for four weeks. Images are shown for one animal per group (magnification × 4). The scale bar indicates 200 µm. Abbreviations: LC, lean rats fed without ecdysterone; LE, lean rats fed with ecdysterone; OC, obese rats fed without ecdysterone; OE, obese rats fed with ecdysterone.

Haematoxylin- and eosin-stained sections of *M. rectus femoris* in all groups showed a homogenous fibre size distribution and polygonal-shaped muscle fibres (Figure 8), which appeared slightly smaller in the obese groups than in the lean groups.

## 3. Discussion

The present study clearly shows that ecdysterone does not exhibit lipid-lowering actions in the liver and plasma of obese Zucker rats as demonstrated by unaltered triglyceride and cholesterol concentrations between obese rats fed with or without ecdysterone. In addition, no lipid-modulating effects of ecdysterone were found in lean Zucker rats. The lack of effect of ecdysterone on hepatic triglyceride concentrations has been confirmed by the measurement of concentrations of fatty acids from hepatic total lipids, which revealed the expected genotype effect, i.e., strong increases in the concentrations of fatty acids originating from lipogenesis, such as 14:0, 16:0, 16:1 n-7, 18:0, and 18:1 n-9, and decreases in the concentrations of 20:4 n-6 and 22:6 n-3 in the obese rats, compared to the lean rats, but virtually no ecdysterone effect. Moreover, Oil Red O-staining of liver sections revealed a marked accumulation of lipids in the livers of the obese compared to the lean rats, but no differences regarding lipid accumulation and morphology were observed between rats of each genotype fed with or without ecdysterone. Thus, our hypothesis that ecdysterone causes lipid-lowering effects in obese Zucker rats has to be rejected. Recently, genome-wide differential transcriptome analysis of the liver between obese and lean Zucker rats revealed a coordinated induction of many genes involved in fatty acid, triglyceride, and cholesterol synthesis in the liver of obese Zucker rats, compared with lean Zucker rats [17,18], thus largely explaining the development of fatty liver and hyperlipidaemia in obese Zucker rats. In line with this effect on gene expression, hepatic activities of lipogenic and cholesterogenic enzymes, such as G6pd, Fasn, Me, and Hmgcr, were shown to be strongly elevated in obese Zucker rats, compared with lean Zucker rats [17,18]. In the present study, differential transcriptome analysis of the liver confirmed the strong induction of many (>30) lipogenic and cholesterogenic genes, such as *Scd3-like* (44.5-fold), *G6pd* (21.8-fold), *Scd2* (10.5-fold), *Elovl6-like* (10.1-fold), *Elovl6* (9.5-fold), *Gpam* (8.6-fold), *Cd36* (8.4-fold), *Fabp4* (7.0-fold), *Me1* (6.6-fold), and many others, in the liver of obese Zucker rats, compared with lean Zucker rats. In addition, bioinformatic enrichment analysis revealed that several of the most enriched biological process terms and KEGG pathways assigned to genes upregulated in the obese compared to the lean rats were related to lipid synthesis, such as unsaturated fatty acid biosynthetic process, cholesterol biosynthetic process, fatty acid biosynthetic process, and fatty acid elongation. In contrast, only one lipogenic gene (*Fasn*) was slightly reduced (−1.39-fold) in the obese rats fed with ecdysterone compared with those fed without, whereas expression of the vast majority of lipogenic and cholesterogenic genes being upregulated in the obese rats compared with the lean rats, were not affected by ecdysterone. In agreement with this, none of the enriched biological process terms and KEGG pathways identified within the transcripts regulated between obese rats fed with ecdysterone compared with those fed without, were dealing with lipid synthesis. Thus, in connection with the unaltered hepatic lipid concentrations, our findings from transcriptome analysis clearly indicate that ecdysterone exhibits no effect on hepatic lipid synthetic pathways in obese Zucker rats.

While the lack of a liver and plasma lipid-lowering effect of ecdysterone in lean Zucker rats is not surprising because physiologically normal levels of lipids in plasma and liver are unlikely to be reduced, it could be argued that the lack of an ecdysterone effect in obese rats is due to an insufficient dose. However, based on our results from HPLC and MS analyses of ecdysterone indicating the absence of any impurities and based on the fact that the rats of either genotype fed the ecdysterone-supplemented diet received a daily dose of approximately 20 mg ecdysterone per kg body weight, which is within the range of other rodent studies reporting biological effects of ecdysterone, we exclude an insufficient dose as a cause for the lack of an ecdysterone effect on hepatic lipid metabolism in Zucker rats. In fact, in 6-week-old streptozotocin-induced steatotic male Wistar rats, daily intragastric administration of ecdysterone at a dose of 5 mg/kg body weight for a duration of 30 days decreased liver and plasma triglyceride and cholesterol concentrations [14]. Rather, it is a matter of fact that results from animal studies dealing with the effect of ecdysterone on hepatic lipid metabolism are conflicting. In contrast to Naresh Kumar et al. [14], no effect of daily intragastric administration of different ecdysterone doses (5, 10, and 20 mg/kg body weight) for 8 weeks on serum triglyceride and cholesterol concentrations was found in 10-week-old female ovariectomised Sprague Dawley rats fed a high-fat/high-fructose diet [15]. In addition, in two studies with 6-week-old male C57BL/6J mice, 3 weeks-feeding of a high-fat diet supplemented with ecdysterone providing a daily dose of 6 mg/kg body weight did not alter plasma and/or liver triglyceride and cholesterol concentrations, compared to the non-supplemented high-fat diet [12,16]. Interestingly, in the studies from Buniam et al. [15] and Foucault et al. [16], in which ecdysterone failed to reduce high-fat-/high-fructose-diet-induced liver and plasma lipid concentrations, ecdysterone exhibited an antiobesity activity, as evidenced from reduced weights of different adipose tissue depots. Such an antiobesity effect has been also reported in another study with 6-week-old C57BL/6J mice, which were fed a high-fat diet and received a daily ecdysterone dose of 10 mg/kg body weight for 13 weeks, but no effect of ecdysterone on hepatic lipogenesis was found in this study [11]. Thus, the results from Kizelsztein [11], Buniam [15], and Foucault [16] indicate that ecdysterone exerts effects on lipid metabolism in a tissue-specific manner. In the present study, we did not determine the weights of adipose tissue depots of the rats, but the observation that final body weights, body weight gain, and feed intake did not differ between groups of the same genotype fed with or without ecdysterone suggests that ecdysterone had no antiobesity activity in Zucker rats. Despite the age of the experimental animals from the abovementioned studies was clearly younger than in the present study (25-week-old), the older age of the Zucker rats alone cannot sufficiently explain the lack of an ecdysterone effect because ecdysterone also failed to exert lipid-modulating effects in markedly younger animals [12,15,16]. Similarly, the duration of ecdysterone administration appears not to be critical because in the studies with no ecdysterone effect on plasma and/or hepatic lipid concentrations the treatment duration ranged from 3 to 13 weeks [11,12,15,16], while in the study reporting liver and plasma lipid-modulating effects the treatment duration was 4 weeks [14]. One discrepancy between the study from Naresh Kumar et al. [14] and the other studies, which reported no liver and plasma lipid-modulating effects [12,15,16], is that the latter studies investigated the preventive potential of ecdysterone to alleviate the metabolic impairment induced by simultaneous feeding of a high-fat or high-fructose diet. In contrast, Naresh Kumar et al. [14] rather studied the therapeutic potential of ecdysterone to correct metabolic derangements of diabetes, which was induced by streptozotocin injection before the 30-day-period of ecdysterone administration started. This suggests that ecdysterone exerts primarily therapeutic efficacy to correct diabetes-induced metabolic derangements but has no preventive potential to alleviate diet-induced hyperlipidaemia. However, in our study, in which the 4-week-period of ecdysterone administration started at an age of 25 weeks, at which steatosis and hyperlipidaemia had already been developed in the obese Zucker rat, ecdysterone obviously had no therapeutic efficacy. Thus, based on this, it appears that the effect of ecdysterone on hepatic lipid metabolism is highly specific to the experimental model used (i.e., streptozotocin-induced diabetic rat model vs. high-fat-diet-induced mouse model vs. Zucker rat). Albeit not under the special focus of this study, we also evaluated the glycaemic status of the Zucker rats by determining the plasma concentration of fructosamine, a time-averaged indicator of blood glucose levels, in order to clarify if ecdysterone had an effect on glucose metabolism. As expected, the plasma concentration of fructosamine was markedly elevated in the two groups of obese Zucker rats, compared to the two lean Zucker rats, but groups of each genotype fed with or without ecdysterone did not differ. This clearly showed that administration of ecdysterone in the Zucker rat model, unlike in streptozotocin-induced diabetic rats, neither modulates hepatic lipid metabolism nor affects glucose tolerance. Thus, further studies are required to explain the reason for the strong hepatic and plasma lipid-lowering effects of ecdysterone in streptozotocin-induced diabetic rats, but the lack of an ecdysterone effect in obese Zucker rats and diet-induced obese mice. Given that adipose tissue depots were not excised from the rats of this study, future studies should also evaluate if ecdysterone causes an effect on adipose tissue metabolism in Zucker rats, such as in mouse models of diet-induced obesity [15,16].

Apart from the lack of ecdysterone on hepatic expression of lipid synthetic genes, the results from differential transcriptome analysis indicated that ecdysterone causes only very moderate effects on the intermediary metabolism of the liver in Zucker rats of both genotypes. Despite the fact that we identified a great number of transcripts to be differentially expressed between ecdysterone-supplemented and non-supplemented lean rats due to the low filter settings applied, the observation that only two genes were regulated >2.0-fold or <−2.0-fold clearly shows that the effect of ecdysterone on hepatic transcriptome was only weak. Likewise, in obese rats, only seven genes were regulated either >2.0-fold or <−2.0-fold. In line with this weak regulation of hepatic gene expression, bioinformatic enrichment analysis of the ecdysterone-regulated transcripts revealed either no or only a low number of enriched biological process terms and KEGG pathways, respectively. In addition, the highly heterogeneous biological process terms and KEGG pathways identified as enriched, which is likely the result of the weak regulation of a large number of genes by ecdysterone, indicate that ecdysterone did not cause considerable effects on specific pathways in the liver of both lean and obese Zucker rats.

Evidence from several earlier studies exists that ecdysteroids exert anabolic effects in a wide variety of vertebrates, such as mice [19,20], rats [21], pigs [22], and Japanese quails [23]. In line with this, it has been demonstrated that ecdysteroids, including ecdysterone, increase protein synthesis in C2C12 myotubes [24]. Moreover, Gorelick-Feldman et al. [24] showed that daily administration of ecdysterone (50 mg/kg body weight) via gavage for 4 weeks increases front limb grip strength of rats indicating that the protein anabolic effect of ecdysterone translates into improved physical performance. The observations from C2C12 cell incubations that ecdysterone does not bind to the androgen receptor, but the protein anabolic effects of ecdysterone are completely abolished by a PI3K inhibitor suggest that ecdysteroids act on the PI3K pathway which is known to promote skeletal muscle growth [25]. In view of these reported effects, we also determined the weights of selected muscles of the rats of both genotypes and carried out a histological analysis of *M. rectus femoris*. However, similar to other parameters addressed in this study, the weights of different muscles excised, such as *M. rectus femoris*, *M. gastrocnemius*, *M. soleus*, *M. vastus intermedius* and *M. vastus medialis*, and muscle morphology were not affected by ecdysterone supplementation. In contrast, muscle weights of the Zucker rats were clearly affected by the genotype, i.e., muscle weights were lower in the obese rats than in the lean rats, which is in line with earlier reports about obesity-related skeletal muscle changes, including muscle atrophy, a switch towards a faster contractile phenotype and impaired mitochondrial oxidative capacity [26,27,28]. The observation that anti-inflammatory interventions are capable of attenuating these deleterious skeletal muscle changes in obese Zucker rats by inhibiting inflammatory signalling pathways in skeletal muscle [29], highlights the role of obesity-associated chronic inflammation for skeletal muscle atrophy. Our findings, therefore, suggest that ecdysterone has neither anabolic nor anti-catabolic effects on skeletal muscle in Zucker rats.

Interestingly, recent reports showed that feeding of protein-rich insect meal produced from industrialised mass-rearing of the edible species *Tenebrio molitor* markedly decreases liver and/or plasma lipids in obese Zucker rats [17,18,30]. As an important mechanism underlying this lipid-lowering action of *Tenebrio molitor* meal, a marked inhibition of lipid synthetic pathways in the liver has been identified [17,18]. While we have shown recently that the characteristically low methionine concentration of insect meal or a decreased cysteine synthesis secondary to a reduced methionine availability are not causative [30], the results from the present study suggest that ecdysteroids are also not responsible for the lipid-lowering effects of insect meal in obese Zucker rats.

In conclusion, the present study clearly shows that ecdysterone supplementation does not exhibit lipid-lowering actions in the liver and plasma of lean and obese Zucker rats.

## 4. Materials and Methods

### 4.1. Animals and Diets

The animal experiment was approved by the local Animal Care and Use Committee (Permission no. and date: JLU 725_M, 16 September 2019). All experimental procedures described followed established guidelines for the care and handling of laboratory animals. The experiment included 16 male, 25-week-old, homozygous (fa/fa) obese Zucker rats (Crl:ZUC-Lepr^fa^) and 16 male, 25-week-old, heterozygous (fa/+) lean Zucker rats, which were purchased from Charles River (Sulzfeld, Germany). The animals were kept in groups of two animals each under controlled conditions (12-h light:12-h dark, 22 ± 1 °C ambient temperature, 50–60 % relative humidity). The lean rats were randomly assigned to two groups [lean control (LC), lean ecdysterone (LE)] of eight rats each. Additionally, the obese rats were randomly assigned to two groups [obese control (OC), obese ecdysterone (OE)] of eight rats each. All groups received the same basal diet (Table 4), which was sufficient to meet the requirements of the rat for maintenance according to the National Research Council (NRC) [31] and supplemented (groups LE and OE) or not (groups LC and OC) with 0.5 g ecdysterone (provided from Alibaba, China) per kg diet.

**Figure 9:**
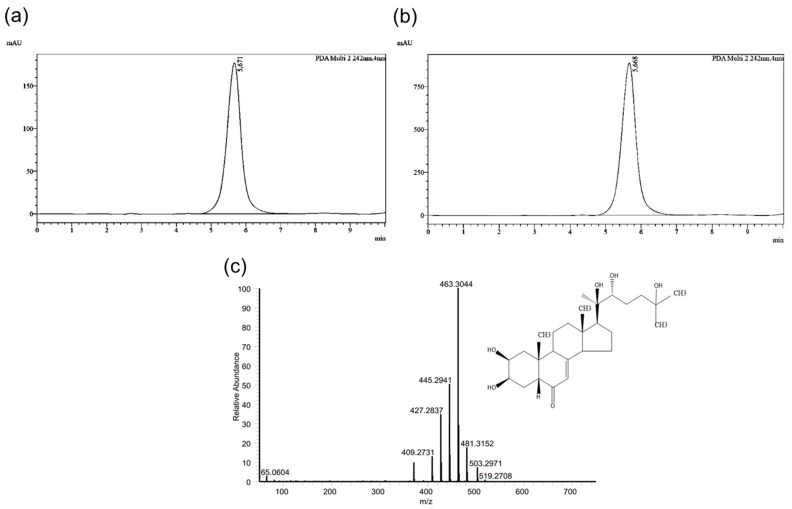
Chromatograms from the HPLC analysis of ecdysterone at a concentration of 150 µg/mL (**a**) and 750 µg/mL (**b**) in MeOH. (**c**) Representative high-resolution total ion current (TIC) chromatogram of ecdysterone.

The chemical characterisation of the aforementioned ecdysterone was carried out by subjecting it to high-performance liquid chromatography (HPLC) according to the method described by Sreejit et al. [32], with slight modifications, and mass spectrometry (MS). In brief, a stock solution of ecdysterone (1.5 mg/mL) was prepared in methanol (MeOH). Two dilutions of the stock solution (150 µg/mL and 750 µg/mL) were prepared and analysed by HPLC on a Prominence system (Shimadzu, Duisburg, Germany) equipped with the HPLC pump LC-20AD, the autosampler SIL-20AC HT, the diode-array-detector (DAD) SPD-M20A, the communication bus module CBM-20A and the LabSolutions Multi LC Data System Manager. An EC 250/4 Nucleosil 100-5 C_18_ column with a matching guard column was used. The injection volume was 20 µL. The mobile phase consisted of MeOH (A) and water (B) in a 1:1 ratio. Analysis was carried out under isocratic conditions, at a flow rate of 0.8 mL/min at ambient temperature. The analysis resulted in a single peak that eluted at approximately 5.67 min, with detection at 242 nm (Figure 9a,b). Ecdysterone was further subjected to liquid chromatography–mass spectrometry (LC–MS) analysis. Reversed-phase ultra-high-performance liquid chromatography separation was performed using an UltiMate 3000 RSLC HPLC system (Thermo Fisher Scientific, Bremen, Germany) on a Kinetex C_18_ (100 × 2.1 mm, 2.6 µm, 100 A particle size) column (Phenomenex, Torrance, CA, USA), coupled with a Q Exactive HF-X (Thermo Scientific, Bremen, Germany) mass spectrometer. Chromatographic analysis was performed at a flow rate of 200 µL/min with the mobile phase described above. Full scan mass spectra were measured in a mass range of m/z 100 to 1000 at a resolution of 240,000 (m/z 200), an automatic gain control target of 3 × 10^6^, and a maximum injection time of 100 ms. High-resolution MS analysis of the sample revealed an exact mass of ecdysterone at m/z of 481.3152 [M+H]^+^ (Figure 9c).

The basal diet contained 19.4 MJ gross energy/kg DM and provided the following crude nutrients as determined by official methods [33] (% DM): crude protein, 21.2; crude fat, 5.6; crude ash, 3.2; crude fibre, 3.8. The rats of all groups had free access to their diets which were fed for 4 weeks. Water was constantly available ad libitum from nipple drinkers.

### 4.2. Sample Collection

Rats were decapitated under CO_2_ anaesthesia in the non-fasted state. Blood was taken up in heparin-coated polyethylene tubes (AppliChem, Darmstadt, Germany) and the plasma was separated from the remaining blood components by centrifugation (1100× *g*, 10 min) at 4 °C. The liver was removed, washed in ice-cold NaCl solution (0.9%), weighed, and several small aliquots were placed in 2 mL reaction tubes and snap frozen in liquid nitrogen. In addition, several whole organs (heart, kidneys, *M. rectus femoris*, *M. gastrocnemius*, *M. soleus*, *M. vastus intermedius*, *M. vastus medialis*) were excised and weighed. From kidneys, perirenal adipose tissue was removed prior to weighing. Plasma and liver samples were stored at −80 °C until analysis.

### 4.3. Determination of TG and Cholesterol Concentrations in Liver and Plasma

Liver samples were ground in a mortar under liquid nitrogen, and lipids extracted from ground liver samples with a mixture of n-hexane and isopropanol (3:2, vol/vol) according to Hara and Radin [34]. The lipid extracts were dried under nitrogen and lipids dissolved with chloroform and Triton X-100 (1:1, *v*/*v*), as described in [35]. Triglyceride and cholesterol concentrations of both, liver lipid extracts and plasma samples were determined using enzymatic reagent kits (Fluitest CHOL, cat. no. 4241, Fluitest TG, cat. no. 5741, both from Analyticon Biotechnologies, Lichtenfels, Germany).

### 4.4. Determination of the Concentrations of Fatty Acids of Hepatic Total Lipids

Concentrations of fatty acids of hepatic total lipids were determined as fatty acid methyl esters by gas chromatography–flame ionisation detection (GC–FID) after transesterification of lipids from hepatic lipid extracts by trimethylsulfonium hydroxide, as described recently in detail [36].

### 4.5. RNA Extraction

Total RNA from frozen liver aliquots (15–20 mg) was isolated using TRIzol reagent (Invitrogen, Karlsruhe, Germany) according to the manufacturer’s protocol and subsequently analysed for quantity and quality using an Infinite 200M microplate reader equipped with a NanoQuant plate (both from Tecan, Mainz, Germany). The average RNA concentration and A260/A280 ratio of all total RNA samples were 2.27 ± 0.4 µg/µL (*n* = 32) and 1.93 ± 0.07 (*n* = 32), respectively.

### 4.6. Microarray Analysis and Bioinformatic Analysis

For microarray analysis, six liver total RNA samples/group were randomly selected. After checking RNA quality [A260:A280 ratios and RNA integrity number values were 1.84 ± 0.05 (mean ± SD) and 7.4 ± 0.6, respectively], as described in [37], RNA samples were processed at the Genomics Core Facility, KFB—Center of Excellence for Fluorescent Bioanalytics (Regensburg, Germany) following the Applied Biosystems GeneChip Whole Transcript (WT) PLUS Reagent Kit User Guide (Thermo Fisher Scientific, Waltham, MA, USA). In brief, 200 ng of total RNA was used to generate double-stranded cDNA. 12 µg of subsequently synthesised cRNA were purified and reverse transcribed into single-stranded (ss) cDNA, whereat unnatural dUTP residues were incorporated. Purified ss cDNA was fragmented using a combination of uracil DNA glycosylase (UDG) and apurinic/apyrimidinic endonuclease 1 (APE 1), followed by terminal labelling with biotin. 3.8 µg of fragmented and labelled ss cDNA were hybridised to Applied Biosystems GeneChip Clariom S rat arrays for 16 h at 45 °C and 60 rpm in an Applied Biosystems GeneChip hybridisation oven 640. Hybridised arrays were washed and stained in an Applied Biosystems GeneChip Fluidics Station FS450, and the fluorescent signals were measured with an Applied Biosystems GeneChip Scanner 3000 7G System. Fluidics and scan functions were controlled by the Applied Biosystems GeneChip Command Console v4.3.3 software. Summarised probe set signals in log_2_ scale were calculated by using the GCCN–SST–RMA algorithm with the Applied Biosystems GeneChip Expression Console v1.4 Software. After exporting into Microsoft Excel, average signal values, comparison fold changes (FC), and significance *p*-values were calculated. The microarray data have been deposited in MIAME compliant format in the NCBI’s Gene Expression Omnibus public repository ([38]; GEO accession no. GSE168390). Owing to the rather moderate differences in the hepatic transcriptomes between groups of the same genotype fed with or without ecdysterone, transcripts with an FC > 1.3 and a Student’s t-test *p*-value < 0.05 were defined as upregulated, and those with aN FC < −1.3 and a Student’s t-test *p*-value < 0.05 as downregulated for the comparison OE vs. OC and LE vs. LC. Identical or similar filter criteria were used in several recent studies [17,37,39], in which transcriptomic alterations caused by the intervention were only moderate and the use of more stringent filter criteria (e.g., false discovery rate and/or FC > 2.0 or < −2.0) failed to identify a substantial number of genes being sufficient to perform gene set enrichment analysis (GSEA). Filtering of differentially expressed transcripts between groups OE vs. OC and LE vs. LC that was based on the Benjamini and Hochberg false discovery rate adjustment method failed because adjusted *p*-values for all transcripts were > 0.05. GSEA was performed with the identified differentially expressed transcripts in order to identify enriched Gene Ontology (GO) terms within GO category biological process using the DAVID 6.8 bioinformatics resource [40,41]. GO terms were defined as enriched if *p* < 0.05. GSEA was performed separately for the up- and downregulated transcripts, respectively.

### 4.7. Validation of Microarray Data Using qPCR Analysis

Microarray data of selected differentially expressed transcripts between groups OC and LC were validated by qPCR. For qPCR analysis, total RNA from all rats (n = 8/group) was used. The cDNA was synthesised, as described recently [42]. The qPCR analysis was carried out with a Rotor-Gene Q system (Qiagen, Hilden, Germany) using gene-specific primer pairs from Eurofins MWG Operon (Ebersberg, Germany), as described recently [42]. Characteristics of primers designed using Primer3 and Basic Local Alignment Search Tool (BLAST) are shown in Supplementary material: Table S5. Normalisation of qPCR data was carried out according to Vandesompele et al. [43] using the three most stable (Actb, Atp5b, Canx) out of seven potential reference genes tested.

### 4.8. Determination of Fructosamine Concentration in Plasma

The fructosamine concentration in plasma was determined as a kinetic measurement at 37 °C using a colorimetric assay kit (Fluitest FRUC, cat. no. 5601, Analyticon Biotechnologies, Lichtenfels, Germany), together with a calibrator (Precimat Fructosamine, Roche Dignastics GmbH, Mannheim, Germany). Absorption was recorded with a microplate reader and the corresponding software (Infinite M200 with i-Control 2.0, both from Tecan, Mainz, Germany).

### 4.9. Histological Analysis of Liver and M. rectus Femoris

Frozen liver and *M*. *rectoris femoris* samples from three animals per group were embedded in Tissue-Tek O.C.T. Compound (Sakura Finetek Europe, AJ Alphen aan den Rijn, the Netherlands) and cryosectioned in 15 µm slices at −20 °C using a CryoStar NX50 microtome (Thermo-Scientific, Germany). Liver and *M. rectus femoris* sections were stained with Oil Red O Stain Kit (ScyTek Laboratories Logan, UT, USA) and Haematoxylin and Eosin Fast Staining Kit (Morphisto, Offenbach, Germany), respectively, according to the manufacturer´s protocol. Stained sections were photographed with an EVOS M5000 microscope (Thermo Fisher Scientific, Dreieich, Germany).

### 4.10. Statistical Analysis

Statistical analyses were performed using the Minitab statistics software (Rel. 13.1, Minitab, State College, PA, USA). All data were checked for normality distribution by an Anderson–Darling test. Normally distributed data were analysed by a two-way ANOVA. Not normally distributed data were log-transformed prior to two-way ANOVA. If the statistical analysis revealed an effect for diet, genotype, or the interaction of diet and genotype, the differences between groups were assessed using the Tukey test. Differences with a significance level of *p* < 0.05 were classified as significant.

## Figures and Tables

**Table 1:** Growth performance and organ weights of lean and obese Zucker rats fed a semisynthetic diet without or with 0.5 g ecdysterone per kg diet for four weeks.

Genotype	Lean		Obese		Two-Way ANOVA *p*-Value		
Ecdysterone (g/kg Diet)	0	0.5	0	0.5	E	G	E × G
Body weight, g							
Initial	441 ± 29 ^b^	446 ± 49 ^b^	570 ± 74 ^a^	561 ± 29 ^a^	0.919	<0.001	0.706
Final	465 ± 34 ^b^	476 ± 51 ^b^	611 ± 79 ^a^	609 ± 32 ^a^	0.821	<0.001	0.746
Daily body weight gain, g	0.86 ± 0.35 ^b^	1.06 ± 0.41 ^b^	1.46 ± 0.36 ^a^	1.69 ± 0.93 ^a^	0.294	0.05	0.928
Daily feed intake, g	20.3 ± 1.2 ^b^	20.8 ± 1.3 ^b^	23.4 ± 1.3 ^a^	25.0 ± 0.9 ^a^	0.103	<0.001	0.361
Feed:gain ratio, g/g	27.9 ± 9.7 ^a^	26.2 ± 13.3 ^a^	16.6 ± 3.7 ^b^	16.0 ± 3.4 ^b^	0.796	0.027	0.905
Organ weights, g							
Heart	1.40 ± 0.08 ^a,b^	1.39 ± 0.08 ^b^	1.52 ± 0.13 ^a^	1.51 ± 0.07 ^a,b^	0.657	0.001	0.948
Kidney right	1.80 ± 0.21 ^b^	1.74 ± 0.30 ^b^	1.97 ± 0.17 ^a^	2.22 ± 0.19 ^a^	0.242	<0.001	0.059
Kidney left	1.79 ± 0.20 ^b^	1.71 ± 0.31 ^b^	1.97 ± 0.14 ^a^	2.26 ± 0.24 ^a^	0.197	<0.001	0.028
Liver	17.9 ± 1.9 ^b^	17.7 ± 2.1 ^b^	33.0 ± 3.6 ^a^	32.5 ± 4.5 ^a^	0.802	<0.001	0.898
*M. soleus*	0.17 ± 0.02 ^a^	0.17 ± 0.01 ^a^	0.12 ± 0.02 ^b^	0.13 ± 0.01 ^b^	0.457	<0.001	0.327
*M. vastus medialis*	0.50 ± 0.09 ^a^	0.54 ± 0.13 ^a^	0.32 ± 0.11 ^b^	0.30 ± 0.09 ^b^	0.717	<0.001	0.424
*M. gastrocnemius*	2.18 ± 0.19 ^a^	2.28 ± 0.15 ^a^	1.58 ± 0.14 ^b^	1.56 ± 0.05 ^b^	0.362	<0.001	0.237
*M. rectus femoris*	1.48 ± 0.56 ^a^	1.39 ± 0.29 ^a^	0.85 ± 0.11 ^b^	0.88 ± 0.07 ^b^	0.802	<0.001	0.609
*M. vastus intermedius*	1.36 ± 0.12 ^a^	1.33 ± 0.17 ^a^	0.87 ± 0.07 ^b^	0.84 ± 0.09 ^b^	0.468	<0.001	0.979

Data are means ± SD; *n* = 8 rats/group (body weight, daily body weight gain, organ weights); *n* = 4 cages/group (daily feed intake and feed:gain ratio). Means not sharing the same letters (^a, b^) differ (*p* < 0.05). Abbreviations: E, ecdysterone; G, genotype.

**Table 2:** Fatty acid concentrations of total lipids in the liver of lean and obese Zucker rats fed a semisynthetic diet without or with 0.5 g ecdysterone per kg diet for four weeks.

Genotype	Lean		Obese		Two-Way ANOVA *p*-Value		
Ecdysterone (g/kg Diet)	0	0.5	0	0.5	E	G	E × G
	µmol/g liver						
14:0	0.66 ± 0.23 ^b^	0.52 ± 0.13 ^b^	3.30 ± 1.11 ^a^	4.08 ± 1.64 ^a^	0.666	<0.001	0.414
14:1 n-5	0.12 ± 0.04 ^b^	0.07 ± 0.03 ^b^	0.46 ± 0.13 ^a^	0.38 ± 0.17 ^a^	0.124	<0.001	0.616
16:0	18.0 ± 3.7 ^b^	14.7 ± 1.9 ^b^	62.2 ± 19.4 ^a^	70.8 ± 28.3 ^a^	0.662	<0.001	0.340
16:1 n-7	2.8 ± 1.4 ^b^	2.2 ± 0.7 ^b^	15.7 ± 5.5 ^a^	17.2 ± 8.3 ^a^	0.819	<0.001	0.552
17:0	0.23 ± 0.07	0.16 ± 0.04	0.52 ± 0.68	0.29 ± 0.11	0.206	0.088	0.482
18:0	11.6 ± 2.2 ^b^	9.9 ± 2.1 ^b^	16.1 ± 2.2 ^a^	17.9 ± 3.0 ^a^	0.604	<0.001	0.026
18:1 n-9	8.5 ± 2.1 ^b^	6.4 ± 1.4 ^b^	48.8 ± 18.6 ^a^	56.0 ± 25.3 ^a^	0.656	<0.001	0.415
18:2 n-6	12.5 ± 3.2	9.1 ± 2.3	10.7 ± 2.7	11.5 ± 3.5	0.752	0.357	0.030
18:3 n-3	0.37 ± 0.13 ^b^	0.25 ± 0.08 ^b^	0.51 ± 0.38 ^a^	0.52 ± 0.30 ^a^	0.531	0.028	0.470
18:3 n-6	0.15 ± 0.02 ^b^	0.11 ± 0.05 ^b^	0.28 ± 0.26 ^a^	0.30 ± 0.12 ^a^	0.908	0.003	0.528
20:3 n-6	0.49 ± 0.09 ^b^	0.41 ± 0.14 ^b^	0.68 ± 0.24 ^a^	0.83 ± 0.28 ^a^	0.659	<0.001	0.113
20:4 n-6	13.4 ± 2.4 ^a^	10.6 ± 1.8 ^b^	9.7 ± 1.9 ^b^	10.5 ± 2.7 ^b^	0.221	0.027	0.034
22:5 n-3	0.40 ± 0.08 ^a^	0.28 ± 0.06 ^b^	0.26 ± 0.09 ^b^	0.25 ± 0.05 ^b^	0.017	0.057	0.120
22:6 n-3	3.31 ± 0.68 ^a^	2.60 ± 0.54 ^a^	1.82 ± 0.46 ^b^	1.94 ± 0.52 ^b^	0.140	<0.001	0.042
∑ total fatty acids	72.4 ± 12.3 ^b^	57.3 ± 8.8 ^b^	170.7 ± 50.2 ^a^	194.5 ± 74.4 ^a^	0.790	<0.001	0.236

Data are means ± SD for *n* = 8 rats per group. Means not sharing the same letters (^a,b^) differ (*p* < 0.05). Abbreviations: E, ecdysterone; G, genotype.

**Table 3:** Regulation of genes with involvement in lipid synthetic pathways in the liver of groups OC vs. LC and groups OE vs. OC.

		OC vs. LC		OE vs. OC	
Gene Symbol	Lipid Synthetic Pathway	FC	*p*-Value	FC	*p*-Value
*Scd3-like*	Fatty acids	44.50	0.0007	not regulated	n.s.
*G6pd*	Fatty acids	21.80	<0.0001	not regulated	n.s.
*Scd2*	Fatty acids	10.50	0.0053	not regulated	n.s.
*Elovl6-like*	Fatty acids	10.14	0.0026	not regulated	n.s.
*Elovl6*	Fatty acids	9.49	0.0032	not regulated	n.s.
*Gpam*	Triglycerides	8.64	<0.0001	not regulated	n.s.
*Cd36*	Triglycerides	8.42	<0.000	not regulated	n.s.
*Fabp4*	Triglycerides	6.96	0.0001	not regulated	n.s.
*Me1*	Fatty acids	6.61	0.0006	not regulated	n.s.
*Fabp5*	Triglycerides	4.72	0.0001	not regulated	n.s.
*Fabp2*	Triglycerides	3.92	<0.0001	not regulated	n.s.
*Fasn*	Fatty acids	3.64	0.0051	−1.39	0.0381
*Dgat2*	Triglycerides	3.26	0.0043	not regulated	n.s.
*Acss2*	Fatty acids	2.62	0.0095	not regulated	n.s.
*Acsl5*	Fatty acids	2.57	0.0001	not regulated	n.s.
*Agpat3*	Phospholipids	2.54	<0.0001	not regulated	n.s.
*Degs1*	Fatty acids	2.38	<0.0001	not regulated	n.s.
*Fdps*	Cholesterol	2.37	0.0350	not regulated	n.s.
*Srebf1*	Fatty acids	2.36	0.0003	not regulated	n.s.
*Pctp*	Phospholipids	2.31	0.0008	not regulated	n.s.
*Lss*	Cholesterol	2.18	0.0037	not regulated	n.s.
*Acaca*	Fatty acids	2.08	0.0264	not regulated	n.s.
*Fads2*	Fatty acids	2.07	<0.0001	not regulated	n.s.
*Dhcr7*	Cholesterol	2.05	0.0447	not regulated	n.s.
*Acly*	Fatty acids	1.92	0.0042	not regulated	n.s.
*Acat2*	Fatty acids	1.87	0.0026	not regulated	n.s.
*Ldlr*	Cholesterol	1.63	0.0268	not regulated	n.s.
*Elovl2*	Fatty acids	1.62	0.0011	not regulated	n.s.
*Pmvk*	Cholesterol	1.62	0.0256	not regulated	n.s.
*Elovl5*	Fatty acids	1.62	0.0013	not regulated	n.s.
*Scd1*	Fatty acids	1.61	0.0055	not regulated	n.s.
*Aacs*	Cholesterol	1.56	0.0143	not regulated	n.s.

Abbreviations: FC, fold change; LC, lean rats fed without ecdysterone; LE, lean rats fed with ecdysterone; OC, obese rats fed without ecdysterone; OE, obese rats fed with ecdysterone; n.s., not significant (*p* > 0.05).

**Table 4:** Composition and nutrient and energy contents of the basal diet.

Components (g/kg)	Basal Diet
Cornstarch	555
Casein	200
Sucrose	100
Soybean oil	50
Cellulose	50
Mineral mix ^1^	35
Vitamin mix ^2^	10

^1^ The mineral mix provided the following per kg diet: calcium, 5 g; potassium, 3.6 g; chloride, 1.57 g; phosphorus, 1.56 g; sodium, 1.02 g; magnesium, 0.51 g; iron, 35 mg; zinc, 30 mg; manganese, 10 mg; copper, 6 mg; chromium, 1 mg; fluoride, 1 mg; iodate, 0.2 mg; molybdate, 0.15 mg; selenium; 0.15 mg; lithium, 0.10 mg. ^2^ The vitamin mix provided the following per kg diet: all-trans-retinol, 1.2 mg; cholecalciferol, 0.025 mg; menadione sodium bisulphate, 0.75 mg; all-rac-α tocopheryl acetate, 50 mg; thiamine HCl, 5 mg; riboflavin, 6 mg; pyridoxine HCl, 6 mg; cyanocobalamin, 0.025 mg; biotin, 0.2 mg; folic acid, 2 mg; nicotinic acid, 30 mg; pantothenic acid, 15 mg; choline, 1000 mg.

## Data Availability

The microarray data have been deposited in MIAME compliant format in the NCBI’s Gene Expression Omnibus public repository (GEO accession no. GSE168390). The other datasets used and analysed during the current study are available from the corresponding author on reasonable request.

## Supplementary Materials

The following are available online at https://www.mdpi.com/article/10.3390/ijms22105241/s1, Table S1: Fold change and *p*-value of all differentially expressed transcripts between groups OC vs. LC, Table S2: Fold change and *p*-value of all differentially expressed transcripts between groups LE vs. LC, Table S3: Fold change and *p*-value of all differentially expressed transcripts between groups OE vs. OC, Table S4: qPCR validation of microarray data for selected differentially expressed transcripts (FC > 1.3 or <−1.3, *p* < 0.05) hepatic transcripts between the groups OC vs. LC, Table S5: Characteristics of gene-specific primers used for qPCR analysis.
