# Supplementary material for: Effect of Ecdysterone on the Hepatic Transcriptome and Lipid Metabolism in Lean and Obese Zucker Rats
Source: Int J Mol Sci. 2021 May 15;22(10):5241. doi: 10.3390/ijms22105241 (PMC8156757; doi:10.3390/ijms22105241)
Supplement: Supplementary file 1 [file ijms-22-05241-s001.zip › ijms-1164561-SI.pdf]

Supplementary file 1

**Table S1	Fold change (FC) and *P*-value of all differentially expressed transcripts (FC > 1.3 or < -1.3, *P* < 0.05) between groups OC vs. LC**

Gene Symbol	Gene Description	FC	*P*-value
LOC681458	similar to stearoyl-coenzyme A desaturase 3	44.50	0.0007
Prlr	prolactin receptor	25.72	0.0003
G6pd	glucose-6-phosphate dehydrogenase	21.80	0.0000
Pnpla3	patatin-like phospholipase domain containing 3	11.36	0.0011
Scd2	stearoyl-Coenzyme A desaturase 2	10.50	0.0053
Cyp17a1	cytochrome P450, family 17, subfamily a, polypeptide 1	10.16	0.0000
LOC102549542	elongation of very long chain fatty acids protein 6-like	10.14	0.0026
Wfdc21	WAP four-disulfide core domain 21	9.58	0.0000
Elovl6	ELOVL fatty acid elongase 6 [Source:RGD Symbol;Acc:620585]	9.52	0.0036
Elovl6	ELOVL fatty acid elongase 6	9.49	0.0032
Gpam	glycerol-3-phosphate acyltransferase, mitochondrial	8.64	0.0000
Cd36	CD36 molecule (thrombospondin receptor)	8.42	0.0000
Rtn4	reticulon 4	7.96	0.0000
Ces2e	carboxylesterase 2E	7.54	0.0003
Edil3	EGF-like repeats and discoidin I-like domains 3	7.54	0.0000
Sult4a1	sulfotransferase family 4A, member 1	7.26	0.0003
Fabp4	fatty acid binding protein 4, adipocyte	6.96	0.0001
Me1	malic enzyme 1, NADP(+)-dependent, cytosolic	6.61	0.0006
Ubd	ubiquitin D	6.54	0.0005
Sult2a6	sulfotransferase family 2A, dehydroepiandrosterone (DHEA)-preferring, member 6	6.03	0.0000
Cyp2c24	cytochrome P450, family 2, subfamily c, polypeptide 24	5.94	0.0102
Tor3a	torsin family 3, member A	5.51	0.0006
Anxa5	annexin A5	5.41	0.0000
Enpp5	ectonucleotide pyrophosphatase/phosphodiesterase 5	5.13	0.0002
Apitd1	apoptosis-inducing, TAF9-like domain 1 (Apitd1), mRNA	4.93	0.0001
Fabp5	fatty acid binding protein 5, epidermal	4.72	0.0001
Trim24	transcription intermediary factor 1-alpha [Source:RefSeq peptide;Acc:NP_001037731]	4.72	0.0004
Efcab6	EF-hand calcium binding domain 6	4.58	0.0005
Aldh1a7	aldehyde dehydrogenase family 1, subfamily A7	4.53	0.0011
Cidea	cell death-inducing DFFA-like effector a	4.44	0.0010
Scpep1	serine carboxypeptidase 1	4.40	0.0000
Angptl8	angiopoietin-like 8	4.38	0.0034
Aldh1a1	aldehyde dehydrogenase 1 family, member A1	4.35	0.0028
Ccdc141	coiled-coil domain containing 141	4.19	0.0003
LOC100912485	alcohol sulfotransferase-like	4.13	0.0020
Fabp2	fatty acid binding protein 2, intestinal	3.92	0.0000
Sdr16c6	short chain dehydrogenase/reductase family 16C, member 6	3.81	0.0362
Otub2	OTU deubiquitinase, ubiquitin aldehyde binding 2	3.78	0.0002
Sult2a2	sulfotransferase family 2A, dehydroepiandrosterone (DHEA)-preferring, member 2	3.77	0.0014
Fasn	fatty acid synthase	3.64	0.0051
LOC100361492	cytochrome P450, family 2, subfamily c, polypeptide 55-like	3.60	0.0372
Mfsd2a	major facilitator superfamily domain containing 2A	3.48	0.0084
Gsta3	glutathione S-transferase alpha 3	3.47	0.0088
P2rx5	purinergic receptor P2X, ligand-gated ion channel, 5	3.29	0.0000
Reep6	receptor accessory protein 6	3.28	0.0004
Apitd1	apoptosis-inducing, TAF9-like domain 1	3.27	0.0016
Dgat2	diacylglycerol O-acyltransferase 2	3.26	0.0043

Inf2	inverted formin, FH2 and WH2 domain containing [Source:MGI Symbol;Acc:MGI:1917685]	3.19	0.0004
Hspa4l	heat shock protein 4-like	3.14	0.0034
Ralyl	RALY RNA binding protein-like	3.03	0.0012
---	Protein LOC367485	3.01	0.0308
Plin3	perilipin 3	2.99	0.0000
Asns	asparagine synthetase (glutamine-hydrolyzing)	2.94	0.0108
Esm1	endothelial cell-specific molecule 1	2.94	0.0044
Srd5a1	steroid-5-alpha-reductase, alpha polypeptide 1 (3-oxo-5 alpha-steroid delta 4-dehydrogenase alpha 1)	2.92	0.0042
---	Protein LOC689635	2.90	0.0368
---	Uncharacterized protein	2.90	0.0049
LOC100911413	deleted in malignant brain tumors 1 protein-like	2.88	0.0000
Hbb-b1	hemoglobin, beta adult major chain	2.84	0.0034
Il17rb	interleukin 17 receptor B	2.82	0.0153
Lgals3	lectin, galactoside-binding, soluble, 3	2.80	0.0000
Myadm	myeloid-associated differentiation marker	2.80	0.0047
Hbb	hemoglobin, beta	2.79	0.0036
Filip1l	filamin A interacting protein 1-like	2.79	0.0026
Kcnn2	potassium channel, calcium activated intermediate/small conductance subfamily N alpha, member 2	2.77	0.0024
Dcakd	dephospho-CoA kinase domain containing	2.74	0.0000
Ces2a	carboxylesterase 2A [Source:RGD Symbol;Acc:708353]	2.74	0.0021
Rgs5	regulator of G-protein signaling 5	2.74	0.0001
LOC100909409	disks large homolog 5-like [Source:RGD Symbol;Acc:6504293]	2.73	0.0168
Aqp11	aquaporin 11	2.72	0.0003
Psat1	phosphoserine aminotransferase 1	2.72	0.0003
Hebp2	heme binding protein 2	2.72	0.0010
LOC100365047	scavenger receptor class B member 2-like	2.71	0.0032
Sri	sorcin	2.69	0.0002
Adh4	alcohol dehydrogenase 4 (class II), pi polypeptide	2.68	0.0017
LOC100912165	rho guanine nucleotide exchange factor 9-like	2.65	0.0000
Casp4	caspase 4, apoptosis-related cysteine peptidase	2.64	0.0028
Acss2	acyl-CoA synthetase short-chain family member 2	2.62	0.0095
Anxa2	annexin A2	2.61	0.0011
RGD1565143	similar to serine/threonine kinase	2.61	0.0032
Ttc24	tetratricopeptide repeat domain 24 [Source:RGD Symbol;Acc:2319887]	2.59	0.0055
Nr0b2	nuclear receptor subfamily 0, group B, member 2	2.59	0.0021
Sall1	spalt-like transcription factor 1	2.59	0.0200
Acsl5	acyl-CoA synthetase long-chain family member 5	2.57	0.0001
Mmp12	matrix metallopeptidase 12	2.56	0.0316
Car2	carbonic anhydrase 2	2.55	0.0001
RGD1306739	similar to RIKEN cDNA 1700040L02	2.54	0.0017
Agpat3	1-acylglycerol-3-phosphate O-acyltransferase 3	2.54	0.0000
Abhd1	abhydrolase domain containing 1	2.50	0.0007
Abcc9	ATP-binding cassette, subfamily C (CFTR/MRP), member 9	2.49	0.0005
Ethe1	ethylmalonic encephalopathy 1	2.48	0.0092
Hba-a1	hemoglobin alpha, adult chain 1	2.47	0.0078
Kank2	KN motif and ankyrin repeat domains 2	2.47	0.0003
Ankrd39	ankyrin repeat domain 39	2.46	0.0000
Agtpbp1	ATP/GTP binding protein 1	2.45	0.0014
Ehhadh	enoyl-CoA, hydratase/3-hydroxyacyl CoA dehydrogenase	2.43	0.0024
Abca2	ATP-binding cassette, subfamily A (ABC1), member 2	2.40	0.0016
Smim1	small integral membrane protein 1	2.39	0.0001
Nudt7	nudix (nucleoside diphosphate linked moiety X)-type motif 7	2.39	0.0000
LOC685203	hypothetical protein LOC685203	2.39	0.0012
Mknk2	MAP kinase-interacting serine/threonine kinase 2	2.38	0.0004
LOC689065	hypothetical protein LOC689065	2.38	0.0163

Degs1	delta(4)-desaturase, sphingolipid 1	2.38	0.0000
Fdps	farnesyl diphosphate synthase	2.37	0.0350
Parvb	parvin, beta	2.36	0.0062
Srebf1	sterol regulatory element binding transcription factor 1	2.36	0.0003
Hbb-b1	hemoglobin, beta adult major chain [Source:RGD Symbol;Acc:1595848]	2.36	0.0176
Dlat	dihydrolipoamide S-acetyltransferase	2.36	0.0114
Adamdec1	ADAM-like, decysin 1	2.34	0.0299
LOC100911315	DNA-binding protein RFX6-like	2.33	0.0001
Ebpl	emopamil binding protein-like	2.31	0.0003
Pdlim1	PDZ and LIM domain 1	2.31	0.0000
Pnpla5	patatin-like phospholipase domain containing 5	2.31	0.0042
Orm1	orosomucoid 1	2.31	0.0024
Pctp	phosphatidylcholine transfer protein	2.31	0.0008
Crot	carnitine O-octanoyltransferase	2.29	0.0318
Cd209b	CD209b antigen	2.29	0.0000
Tagln	transgelin	2.28	0.0050
Atp8a1	ATPase, aminophospholipid transporter (APLT), class I, type 8A, member 1	2.28	0.0003
Gypa	glycophorin A	2.28	0.0009
Pex11a	peroxisomal biogenesis factor 11 alpha	2.27	0.0003
Topbp1	topoisomerase (DNA) II binding protein 1	2.27	0.0000
Rfx6	regulatory factor X, 6	2.27	0.0000
Bmp7	bone morphogenetic protein 7	2.27	0.0452
Chst9	carbohydrate (N-acetylgalactosamine 4-0) sulfotransferase 9	2.26	0.0002
Acer2	alkaline ceramidase 2	2.25	0.0000
Mfhas1	malignant fibrous histiocytoma amplified sequence 1	2.24	0.0021
Ptk2b	protein tyrosine kinase 2 beta	2.24	0.0033
Itpk1	inositol-tetrakisphosphate 1-kinase	2.24	0.0011
Fut8	fucosyltransferase 8 (alpha (1,6) fucosyltransferase)	2.24	0.0007
Tm7sf2	transmembrane 7 superfamily member 2	2.22	0.0200
Steap3	STEAP family member 3, metalloreductase	2.20	0.0048
Plin2	perilipin 2	2.20	0.0140
Gda	guanine deaminase	2.20	0.0109
Clybl	citrate lyase beta like	2.19	0.0000
Lss	lanosterol synthase (2,3-oxidosqualene-lanosterol cyclase)	2.18	0.0037
Aco1	aconitase 1, soluble	2.18	0.0003
Dmrta1	DMRT-like family A1	2.17	0.0001
Src	SRC proto-oncogene, non-receptor tyrosine kinase	2.17	0.0008
Cyp2c22	cytochrome P450, family 2, subfamily c, polypeptide 22	2.15	0.0064
Kbtbd12	Protein LOC502859	2.15	0.0442
Igfn1	immunoglobulin-like and fibronectin type III domain containing 1	2.15	0.0079
Copz2	coatomer protein complex, subunit zeta 2	2.14	0.0000
Cgref1	cell growth regulator with EF hand domain 1	2.14	0.0002
Ephx2	epoxide hydrolase 2, cytoplasmic	2.14	0.0015
Med12l	mediator complex subunit 12-like	2.14	0.0000
RGD1559459	similar to Expressed sequence AI788959	2.13	0.0160
Nrp2	neuropilin 2	2.13	0.0001
Apoa1	apolipoprotein A-I	2.12	0.0005
Gjb2	gap junction protein, beta 2	2.11	0.0359
Slc19a3	solute carrier family 19 (thiamine transporter), member 3	2.10	0.0169
Adora1	adenosine A1 receptor	2.10	0.0299
LOC100365112	carboxylesterase-like	2.09	0.0069
Ear11	eosinophil-associated, ribonuclease A family, member 11	2.09	0.0222
Acaca	acetyl-CoA carboxylase alpha	2.08	0.0264
Fads2	fatty acid desaturase 2	2.07	0.0000
Spsb4	splA/ryanodine receptor domain and SOCS box containing 4	2.07	0.0080
Plbd1	phospholipase B domain containing 1	2.07	0.0068
Vnn1	vanin 1	2.06	0.0176
Cabp2	calcium binding protein 2	2.06	0.0008

Car7	carbonic anhydrase 7	2.05	0.0033
LOC100134871	beta globin minor gene	2.05	0.0144
Dhcr7	7-dehydrocholesterol reductase	2.05	0.0447
Hadhb	hydroxyacyl-CoA dehydrogenase/3-ketoacyl-CoA thiolase/enoyl-CoA hydratase (trifunctional protein), beta subunit	2.04	0.0000
Lss	ENCODES a protein that exhibits lanosterol synthase activity AND INVOLVED IN cholesterol biosynthetic process	2.04	0.0227
Cxcl14	chemokine (C-X-C motif) ligand 14	2.04	0.0114
Slc39a4	solute carrier family 39 (zinc transporter), member 4	2.04	0.0034
Zc3h12d	zinc finger CCCH type containing 12D	2.03	0.0015
Desi1	desumoylating isopeptidase 1	2.03	0.0001
---	beta-globin (LOC689064), mRNA	2.03	0.0068
Lamb3	laminin, beta 3	2.02	0.0014
Nceh1	neutral cholesterol ester hydrolase 1	2.02	0.0026
Acsm2a	acyl-CoA synthetase medium-chain family member 2A	2.00	0.0320
Srpx	sushi-repeat-containing protein, X-linked	2.00	0.0000
Ppp1r3b	protein phosphatase 1, regulatory subunit 3B	2.00	0.0036
Alas2	5-aminolevulinate synthase 2	2.00	0.0009
Arhgef9	Cdc42 guanine nucleotide exchange factor (GEF) 9	1.99	0.0068
Ptprs	protein tyrosine phosphatase, receptor type, S	1.99	0.0025
Limk2	LIM domain kinase 2	1.99	0.0060
Irs3	insulin receptor substrate 3	1.98	0.0153
St3gal3	ST3 beta-galactoside alpha-2,3-sialyltransferase 3	1.98	0.0012
Dbi	diazepam binding inhibitor (GABA receptor modulator, acyl-CoA binding protein)	1.98	0.0047
Rarb	retinoic acid receptor, beta	1.97	0.0064
Commd6	COMM domain containing 6	1.97	0.0280
Mest	mesoderm specific transcript	1.97	0.0001
Aldh1l2	aldehyde dehydrogenase 1 family, member L2	1.97	0.0001
LOC691083	hypothetical protein LOC691083	1.97	0.0067
Aig1	androgen-induced 1	1.96	0.0030
Ugt2b1	UDP glucuronosyltransferase 2 family, polypeptide B1	1.96	0.0105
Trim24	tripartite motif-containing 24	1.96	0.0259
LOC102550654	disintegrin and metalloproteinase domain-containing protein 28-like	1.95	0.0320
Dcn	decorin	1.94	0.0179
Fbln5	fibulin 5	1.93	0.0015
Ncald	neurocalcin delta	1.93	0.0012
Acly	ATP citrate lyase	1.92	0.0042
Rab30	RAB30, member RAS oncogene family	1.92	0.0472
Nupr1	nuclear protein, transcriptional regulator, 1	1.91	0.0004
LOC681469	similar to stearoyl-Coenzyme A desaturase 2	1.90	0.0006
Ces4a	carboxylesterase 4A	1.90	0.0137
Jam2	junctional adhesion molecule 2	1.90	0.0004
Sult1b1	sulfotransferase family, cytosolic, 1B, member 1	1.89	0.0008
Angpt1	angiopoietin 1	1.89	0.0001
RGD1564958	PARTICIPATES IN Alzheimer disease pathway AND gluconeogenesis pathway	1.89	0.0012
Lhx2	LIM homeobox 2	1.88	0.0129
Laptm4b	lysosomal protein transmembrane 4 beta	1.88	0.0012
Pfkfb1	6-phosphofructo-2-kinase/fructose-2,6-biphosphatase 1	1.88	0.0403
Cd93	CD93 molecule	1.88	0.0006
Adssl1	Adenylosuccinate synthetase isozyme 1	1.87	0.0073
Slc4a1	solute carrier family 4 (anion exchanger), member 1	1.87	0.0043
Acat2	acetyl-CoA acetyltransferase 2	1.87	0.0026
Adgrg6	adhesion G protein-coupled receptor G6	1.87	0.0075
NUPR1	nuclear protein, transcriptional regulator, 1 (Nupr1), mRNA	1.87	0.0038
Tmem135	transmembrane protein 135	1.86	0.0017
Rassf9	Ras association (RalGDS/AF-6) domain family (N-terminal) member 9	1.86	0.0001

Sparc	secreted protein, acidic, cysteine-rich (osteonectin)	1.86	0.0002
Pak1	p21 protein (Cdc42/Rac)-activated kinase 1	1.86	0.0001
LOC102550773	rho GTPase-activating protein 20-like	1.85	0.0002
Aldh1b1	aldehyde dehydrogenase 1 family, member B1	1.85	0.0037
Paqr8	progestin and adipoQ receptor family member VIII	1.84	0.0028
Apmap	adipocyte plasma membrane associated protein	1.84	0.0089
Acot2	acyl-CoA thioesterase 2	1.84	0.0281
LOC686437	similar to Protein C14orf105	1.84	0.0043
Nt5c2	5-nucleotidase, cytosolic II	1.84	0.0004
Sdc1	syndecan 1	1.84	0.0133
Pcdhga4	protocadherin gamma subfamily A, 4	1.84	0.0055
Cemip	cell migration inducing protein, hyaluronan binding	1.84	0.0034
Zdhhc2	zinc finger, DHHC-type containing 2	1.84	0.0061
Arhgap28	Rho GTPase activating protein 28	1.83	0.0279
Sesn3	sestrin 3	1.83	0.0003
Tk1	thymidine kinase 1, soluble	1.83	0.0081
Hpse	heparanase	1.83	0.0057
Perp	PERP, TP53 apoptosis effector	1.83	0.0005
Tspo	translocator protein	1.82	0.0007
Tfrc	transferrin receptor	1.81	0.0105
Hfe2	hemochromatosis type 2 (juvenile)	1.81	0.0442
Lrrfip1	leucine rich repeat (in FLII) interacting protein 1	1.81	0.0252
Mybl1	myeloblastosis oncogene-like 1	1.81	0.0387
Pex3	peroxisomal biogenesis factor 3	1.81	0.0022
Ugt2b10	UDP glucuronosyltransferase 2 family, polypeptide B10	1.81	0.0067
Tmem97	transmembrane protein 97	1.81	0.0020
Adh1	alcohol dehydrogenase 1 (class I)	1.81	0.0076
Adh7	alcohol dehydrogenase 7 (class IV), mu or sigma polypeptide	1.81	0.0299
Dhdds	dehydrodolichyl diphosphate synthase subunit	1.80	0.0000
Klrg1	killer cell lectin-like receptor subfamily G, member 1	1.80	0.0073
Mki67	marker of proliferation Ki-67	1.80	0.0140
Bpgm	2,3-bisphosphoglycerate mutase	1.80	0.0013
Rnpepl1	arginyl aminopeptidase (aminopeptidase B)-like 1	1.80	0.0023
Tmem120a	transmembrane protein 120A	1.80	0.0071
Rpa1	replication protein A1	1.80	0.0010
Tspyl4	TSPY-like 4	1.80	0.0182
Olr43	olfactory receptor 43	1.80	0.0061
Tmem64	transmembrane protein 64	1.80	0.0029
Ccdc85c	coiled-coil domain containing 85C	1.80	0.0032
Acta2	actin, alpha 2, smooth muscle, aorta	1.80	0.0009
RGD1566091	INTERACTS WITH dibutyl phthalate	1.80	0.0003
Urad	ureidoimidazoline (2-oxo-4-hydroxy-4-carboxy-5-) decarboxylase [Source:RGD Symbol;Acc:1595191]	1.79	0.0025
LOC102553986	retinoic acid receptor RXR-gamma-like	1.79	0.0084
Abcd3	ATP-binding cassette, subfamily D (ALD), member 3	1.78	0.0007
Cyb5b	cytochrome b5 type B (outer mitochondrial membrane)	1.78	0.0275
Amd1	adenosylmethionine decarboxylase 1	1.78	0.0432
Acot9	acyl-CoA thioesterase 9	1.78	0.0139
Tmem252	transmembrane protein 252	1.78	0.0206
Olr1029	olfactory receptor 1029	1.78	0.0050
Apoa4	apolipoprotein A-IV	1.78	0.0001
Glt1d1	glycosyltransferase 1 domain containing 1	1.78	0.0009
Akr1b7	aldo-keto reductase family 1, member B7	1.77	0.0349
Mapk9	mitogen-activated protein kinase 9	1.77	0.0010
Ccdc79	Uncharacterized protein [Source:UniProtKB/TrEMBL;Acc:F1M8N4]	1.77	0.0032
Ucp2	uncoupling protein 2 (mitochondrial, proton carrier)	1.77	0.0028
Gls	glutaminase	1.77	0.0058
Unc5b	unc-5 netrin receptor B	1.77	0.0461

Tfpi	tissue factor pathway inhibitor (lipoprotein-associated coagulation inhibitor)	1.77	0.0007
RGD1560242	similar to RIKEN cDNA 1700028P14	1.76	0.0059
Edem1	Protein Edem1	1.76	0.0053
Pdk1	pyruvate dehydrogenase kinase, isozyme 1	1.76	0.0020
Anp32a	acidic (leucine-rich) nuclear phosphoprotein 32 family, member A	1.76	0.0003
Abhd6	abhydrolase domain containing 6	1.75	0.0453
Pla2g2a	phospholipase A2, group IIA (platelets, synovial fluid)	1.75	0.0096
Spink3	serine peptidase inhibitor, Kazal type 3	1.75	0.0273
Eif2ak4	eukaryotic translation initiation factor 2 alpha kinase 4	1.75	0.0014
Gtse1	G-2 and S-phase expressed 1	1.75	0.0032
LOC689064	beta-globin	1.75	0.0098
Psmb9	proteasome subunit beta 9	1.74	0.0122
Secisbp2l	SECIS binding protein 2-like	1.74	0.0088
Sytl2	synaptotagmin-like 2 [Source:RGD Symbol;Acc:1307647]	1.74	0.0170
LOC102552996	uncharacterized LOC102552996	1.74	0.0213
Spc24	SPC24, NDC80 kinetochore complex component	1.74	0.0008
Cyb5r3	cytochrome b5 reductase 3	1.74	0.0149
Pcdhb21	protocadherin beta 21	1.74	0.0379
RGD1311756	similar to hypothetical protein FLJ20950	1.74	0.0046
Mdh1	malate dehydrogenase 1, NAD (soluble)	1.73	0.0033
Smoc1	SPARC related modular calcium binding 1	1.73	0.0014
Cd99l2	CD99 molecule-like 2	1.73	0.0015
Npc2	Niemann-Pick disease, type C2	1.73	0.0001
Acot1	acyl-CoA thioesterase 1	1.73	0.0088
Aifm2	apoptosis-inducing factor, mitochondrion-associated 2	1.72	0.0078
Inhbe	inhibin beta E	1.72	0.0103
Itga9	integrin, alpha 9 [Source:RGD Symbol;Acc:1311191]	1.72	0.0159
Slc9a9	solute carrier family 9, subfamily A (NHE9, cation proton antiporter 9), member 9	1.72	0.0072
RGD1562758	similar to glyceraldehyde-3-phosphate dehydrogenase [Source:RGD Symbol;Acc:1562758]	1.72	0.0008
Thrsp	thyroid hormone responsive	1.72	0.0098
Ablim1	actin-binding LIM protein 1 [Source:MGI Symbol;Acc:MGI:1194500]	1.72	0.0227
Plk1	polo-like kinase 1	1.72	0.0085
LOC100362607	nidogen 2	1.72	0.0000
LOC500300	similar to hypothetical protein MGC6835	1.72	0.0336
LOC498300	similar to degenerative spermatocyte homolog	1.72	0.0038
Vsig10	V-set and immunoglobulin domain containing 10	1.72	0.0038
Agfg2	ArfGAP with FG repeats 2	1.71	0.0033
Gosr2	golgi SNAP receptor complex member 2	1.71	0.0001
LOC102552497	protocadherin gamma-B6-like	1.71	0.0153
Rdh11	retinol dehydrogenase 11 (all-trans/9-cis/11-cis)	1.71	0.0218
RGD1359508	similar to protein C33A12.3	1.70	0.0031
Krt79	Protein LOC683720	1.70	0.0114
Bche	butyrylcholinesterase	1.70	0.0061
LOC680538	similar to Glyceraldehyde-3-phosphate dehydrogenase (GAPDH)similar to glyceraldehyde-3-phosphate dehydrogenase (phosphorylating) (EC 1.2.1.12)	1.70	0.0004
Slc6a13	solute carrier family 6 (neurotransmitter transporter), member 13	1.70	0.0162
---	Uncharacterized protein	1.70	0.0001
Clcn6	chloride channel, voltage-sensitive 6	1.69	0.0038
RGD1564854	similar to divalent cation tolerant protein CUTA	1.69	0.0047
Wdr76	WD repeat domain 76	1.69	0.0018
Aig1	androgen-induced 1 (Aig1), mRNA. Chalmel, et. al. Testis-expressed Unannotated Transcripts (TUTs)	1.69	0.0149
Galc	galactosylceramidase	1.69	0.0052
Ube3d	ubiquitin protein ligase E3D	1.69	0.0148
Cyp2c7	cytochrome P450, family 2, subfamily c, polypeptide 7	1.69	0.0000
St6gal1	ST6 beta-galactosamide alpha-2,6-sialyltranferase 1	1.69	0.0364

Tlcd2	TLC domain containing 2	1.68	0.0128
Tpm1	tropomyosin 1, alpha	1.68	0.0043
Gale	UDP-galactose-4-epimerase	1.68	0.0148
LOC691807	hypothetical protein LOC691807	1.68	0.0041
Ppp2r2d	protein phosphatase 2, regulatory subunit B, delta	1.68	0.0011
Pcdhga5	protocadherin gamma subfamily A, 5 (Pcdhga5), mRNA. Chalmel, et. al. Testis-expressed Unannotated Transcripts (TUTs)	1.68	0.0134
Pgk1	phosphoglycerate kinase 1	1.68	0.0175
Ptgis	prostaglandin I2 (prostacyclin) synthase	1.68	0.0149
Pigu	phosphatidylinositol glycan anchor biosynthesis, class U	1.68	0.0087
LOC691988	similar to a disintegrin and metalloproteinase domain 28	1.68	0.0073
LOC102554096	guanylate-binding protein 6-like	1.68	0.0470
Cux2	cut-like homeobox 2	1.67	0.0084
Ceacam20	carcinoembryonic antigen-related cell adhesion molecule 20	1.67	0.0197
Col4a1	collagen, type IV, alpha 1	1.67	0.0004
Fbxo3	F-box protein 3	1.67	0.0017
Acadl	acyl-CoA dehydrogenase, long chain	1.67	0.0000
Tmsb10	thymosin, beta 10	1.67	0.0209
LOC100911848	uncharacterized LOC100911848	1.67	0.0004
Serpinb9	serpin peptidase inhibitor, clade B (ovalbumin), member 9	1.66	0.0000
Fgf1	fibroblast growth factor 1 (acidic)	1.66	0.0018
Adm	adrenomedullin	1.66	0.0002
Rangap1	RAN GTPase activating protein 1	1.66	0.0016
Prss8	protease, serine, 8	1.66	0.0442
Rbm46	RNA binding motif protein 46	1.66	0.0055
Scn8a	sodium channel, voltage gated, type VIII, alpha subunit	1.66	0.0023
Nsdhl	NAD(P) dependent steroid dehydrogenase-like	1.66	0.0199
Osbpl11	oxysterol binding protein-like 11	1.66	0.0285
LOC691565	hypothetical protein LOC691565	1.65	0.0445
Col3a1	collagen, type III, alpha 1	1.65	0.0009
Tdh	L-threonine dehydrogenase	1.65	0.0016
Ces2c	carboxylesterase 2C (Ces2c), mRNA	1.65	0.0456
Tmem55a	transmembrane protein 55A	1.65	0.0013
Tmsb10	thymosin, beta 10 [Source:RGD Symbol;Acc:62022]	1.65	0.0225
Allc	allantoicase	1.65	0.0044
Slc8a3	solute carrier family 8 (sodium/calcium exchanger), member 3	1.65	0.0007
Ccng1	cyclin G1	1.65	0.0069
Hemgn	hemogen	1.64	0.0227
Tm6sf2	transmembrane 6 superfamily member 2	1.64	0.0172
LOC102553649	disks large homolog 5-like	1.64	0.0241
Inpp1	inositol polyphosphate-1-phosphatase	1.64	0.0023
Clec4a2	C-type lectin domain family 4, member A2	1.64	0.0005
RGD1311739	similar to RIKEN cDNA 1700037H04	1.64	0.0142
Sacs	sacsin molecular chaperone	1.64	0.0023
Calm2	calmodulin 2 [Source:RGD Symbol;Acc:2258]	1.64	0.0009
Ugt2a3	UDP glucuronosyltransferase 2 family, polypeptide A3	1.64	0.0196
Apln	apelin	1.64	0.0297
Pcdhb20	protocadherin beta 20	1.64	0.0007
RGD1564865	similar to 20-alpha-hydroxysteroid dehydrogenase	1.63	0.0316
Ech1	enoyl CoA hydratase 1, peroxisomal	1.63	0.0440
Suox	sulfite oxidase	1.63	0.0046
Olr901	ENCODES a protein that exhibits olfactory receptor activity (inferred)	1.63	0.0063
Bdh2	3-hydroxybutyrate dehydrogenase, type 2	1.63	0.0083
RGD1309676	similar to RIKEN cDNA 5730469M10	1.63	0.0088
Fbn1	fibrillin 1	1.63	0.0098
Sc5d	sterol-C5-desaturase	1.63	0.0039
L3hypdh	L-3-hydroxyproline dehydratase (trans-)	1.63	0.0290
Prl8a3	INTERACTS WITH astemizole AND bisphenol A AND C60 fullerene	1.63	0.0138

Mphosph6	M phase phosphoprotein 6	1.63	0.0038
Ldlr	low density lipoprotein receptor	1.63	0.0268
Npnt	nephronectin	1.63	0.0038
Elovl2	ELOVL fatty acid elongase 2	1.62	0.0011
Pmvk	phosphomevalonate kinase	1.62	0.0256
Arsi	arylsulfatase family, member I	1.62	0.0200
Plcd3	phospholipase C, delta 3	1.62	0.0327
Atp6v1d	ATPase, H+ transporting, lysosomal V1 subunit D	1.62	0.0014
Il18	interleukin 18	1.62	0.0191
LOC102553032	ellis-van Creveld syndrome protein homolog	1.62	0.0038
Elovl5	ELOVL fatty acid elongase 5	1.62	0.0013
Lnpep	leucyl/cystinyl aminopeptidase	1.62	0.0013
RGD1309730	similar to RIKEN cDNA B230118H07	1.62	0.0014
LOC680097	similar to germinal histone H4 gene	1.62	0.0126
Tfcp2l1	transcription factor CP2-like 1	1.62	0.0171
Reln	reelin	1.61	0.0021
Ctnna3	catenin (cadherin associated protein), alpha 3	1.61	0.0161
Cyp2c6v1	cytochrome P450, family 2, subfamily C, polypeptide 6, variant 1	1.61	0.0003
Adam28	ADAM metallopeptidase domain 28	1.61	0.0032
Glb1l2	galactosidase, beta 1-like 2	1.61	0.0217
Pde8b	phosphodiesterase 8B	1.61	0.0352
Olr824	olfactory receptor 824	1.61	0.0253
Ak4	adenylate kinase 4	1.61	0.0006
Eepd1	endonuclease/exonuclease/phosphatase family domain containing 1	1.61	0.0081
Fstl1	follistatin-like 1	1.61	0.0139
LOC102553394	uncharacterized LOC102553394	1.61	0.0045
Tnfaip2	tumor necrosis factor, alpha-induced protein 2	1.61	0.0424
Rab32	RAB32, member RAS oncogene family	1.61	0.0003
Mttp	microsomal triglyceride transfer protein	1.61	0.0177
Scd1	stearoyl-Coenzyme A desaturase 1	1.61	0.0055
Arpp19	cAMP-regulated phosphoprotein 19	1.61	0.0010
Fzd4	frizzled class receptor 4	1.61	0.0348
F2r	coagulation factor II (thrombin) receptor	1.61	0.0001
Abcc1	ATP-binding cassette, subfamily C (CFTR/MRP), member 1	1.61	0.0210
Bmp1	bone morphogenetic protein 1	1.61	0.0101
Heg1	heart development protein with EGF-like domains 1	1.60	0.0024
Ppp1r14c	protein phosphatase 1, regulatory (inhibitor) subunit 14c	1.60	0.0082
Mrpl23	mitochondrial ribosomal protein L23	1.60	0.0005
LOC691962	hypothetical protein LOC691962	1.60	0.0024
Stx2	syntaxin 2	1.60	0.0475
Golt1a	golgi transport 1A	1.60	0.0145
Timp1	TIMP metallopeptidase inhibitor 1	1.60	0.0245
Plat	plasminogen activator, tissue	1.60	0.0053
Apool	apolipoprotein O-like	1.60	0.0000
Pklr	pyruvate kinase, liver and RBC	1.60	0.0296
Calm2	calmodulin 2	1.60	0.0009
Acaa1	acetyl-CoA acyltransferase 1A	1.59	0.0042
Alpl	alkaline phosphatase, liver/bone/kidney	1.59	0.0405
Agap3	ArfGAP with GTPase domain, ankyrin repeat and PH domain 3	1.59	0.0056
Gpr137b	G protein-coupled receptor 137B	1.59	0.0144
Zfp90	zinc finger protein 90	1.59	0.0052
Bst2	bone marrow stromal cell antigen 2	1.59	0.0431
Tlcd1	TLC domain containing 1	1.59	0.0400
Hltf	helicase-like transcription factor	1.59	0.0049
Ednrb	endothelin receptor type B	1.58	0.0017
Grhl2	grainyhead-like transcription factor 2	1.58	0.0076
Glrx3	glutaredoxin 3	1.58	0.0001
Ociad2	OCIA domain containing 2	1.58	0.0146

Galk1	galactokinase 1	1.58	0.0095
Rxrg	retinoid X receptor gamma	1.58	0.0277
Rbbp8	retinoblastoma binding protein 8	1.58	0.0031
G0s2	G0/G1switch 2	1.58	0.0051
Pkp2	plakophilin 2	1.57	0.0254
Glrx	glutaredoxin (thioltransferase)	1.57	0.0402
Frzb	frizzled-related protein	1.57	0.0181
Dlg3	discs, large homolog 3 (Drosophila)	1.57	0.0070
Mgam	maltase-glucoamylase [Source:MGI Symbol;Acc:MGI:1203495]	1.57	0.0197
Kri1	KRI1 homolog (S. cerevisiae)	1.57	0.0192
Anxa7	annexin A7	1.57	0.0031
Nmt2	N-myristoyltransferase 2	1.57	0.0163
Ddit4	DNA-damage-inducible transcript 4	1.57	0.0116
---	---	1.57	0.0116
Tkt	transketolase	1.57	0.0133
Timp3	TIMP metallopeptidase inhibitor 3	1.56	0.0322
Lactb2	lactamase, beta 2	1.56	0.0325
Ikbke	inhibitor of kappa light polypeptide gene enhancer in B-cells, kinase epsilon	1.56	0.0110
Dll4	delta-like 4 (Drosophila)	1.56	0.0139
Sdcbp	syndecan binding protein	1.56	0.0000
Aacs	acetoacetyl-CoA synthetase	1.56	0.0143
Igfbp7	insulin-like growth factor binding protein 7	1.56	0.0071
LOC100912507	INTERACTS WITH 17beta-estradiol (ortholog) AND 5-azacytidine (ortholog) AND all-trans-retinoic acid (ortholog)	1.56	0.0024
LOC100912223	INTERACTS WITH 17beta-estradiol (ortholog) AND 5-azacytidine (ortholog) AND all-trans-retinoic acid (ortholog)	1.56	0.0024
Olr1252	ENCODES a protein that exhibits olfactory receptor activity (inferred)	1.56	0.0024
Olr279	olfactory receptor 279	1.56	0.0142
Tnnc2	troponin C type 2 (fast)	1.56	0.0001
Cbx3	chromobox homolog 3	1.56	0.0434
Colec10	collectin sub-family member 10 (C-type lectin)	1.56	0.0119
Brk1	BRICK1, SCAR/WAVE actin-nucleating complex subunit	1.56	0.0249
Znf750	zinc finger protein 750	1.56	0.0164
Glrx5	glutaredoxin 5	1.56	0.0013
Vps36	vacuolar protein sorting 36 homolog (S. cerevisiae)	1.56	0.0024
Rbm4	Chalmel, et. al. AceView Annotation Rbm4.gSep08 Testis-expressed Unannotated Transcripts (TUTs)	1.56	0.0074
Enpp4	ectonucleotide pyrophosphatase/phosphodiesterase 4	1.56	0.0171
Pex14	peroxisomal biogenesis factor 14	1.55	0.0025
Dusp22	dual specificity phosphatase 22	1.55	0.0060
RGD1564133	INTERACTS WITH 8-anilinonaphthalene-1-sulfonic acid (ortholog) AND all-trans-retinoic acid (ortholog) AND copper(2+) sulfate (ortholog)	1.55	0.0433
Lrig3	leucine-rich repeats and immunoglobulin-like domains 3	1.55	0.0338
RGD1559683	similar to RIKEN cDNA 1700001C02	1.55	0.0076
Lrrcc1	leucine rich repeat and coiled-coil centrosomal protein 1	1.55	0.0299
Kifc3	kinesin family member C3	1.55	0.0271
Slc25a20	solute carrier family 25 (carnitine/acylcarnitine translocase), member 20	1.55	0.0196
Cln6	ceroid-lipofuscinosis, neuronal 6, late infantile, variant	1.55	0.0192
Ube2e2	ubiquitin-conjugating enzyme E2E 2	1.55	0.0019
Tmem14c	transmembrane protein 14C	1.55	0.0164
LOC102547000	rho GTPase-activating protein 20-like	1.55	0.0376
Exosc9	exosome component 9	1.55	0.0003
Rxfp1	relaxin/insulin-like family peptide receptor 1	1.55	0.0468
Tmem2	transmembrane protein 2	1.54	0.0031
Rcn1	reticulocalbin 1, EF-hand calcium binding domain	1.54	0.0112
Cdr2	cerebellar degeneration-related protein 2	1.54	0.0012
Arhgap11a	Rho GTPase activating protein 11A	1.54	0.0228
Sorcs2	sortilin-related VPS10 domain containing receptor 2	1.54	0.0294

LOC102555951	brain-specific angiogenesis inhibitor 1-associated protein 2-like	1.54	0.0492
Fam134b	family with sequence similarity 134, member B	1.54	0.0201
Ppt1	palmitoyl-protein thioesterase 1	1.54	0.0126
Ctnna3	catenin (cadherin associated protein), alpha 3	1.54	0.0169
LOC102547484	uncharacterized LOC102547484	1.54	0.0295
Acot3	acyl-CoA thioesterase 3	1.54	0.0447
Rtcb	RNA 2,3-cyclic phosphate and 5-OH ligase	1.54	0.0000
Ralgps2	Ral GEF with PH domain and SH3 binding motif 2	1.54	0.0016
Taldo1	transaldolase 1	1.54	0.0159
Mgll	monoglyceride lipase	1.54	0.0112
Hba1	hemoglobin, alpha 1	1.54	0.0081
Cidec	cell death-inducing DFFA-like effector c	1.54	0.0104
Acot4	acyl-CoA thioesterase 4	1.53	0.0247
Otulin	OTU deubiquitinase with linear linkage specificity	1.53	0.0026
Mical2	microtubule associated monooxygenase, calponin and LIM domain containing 2	1.53	0.0183
Cxcl13	chemokine (C-X-C motif) ligand 13	1.53	0.0242
LOC100359600	karyopherin alpha 2-like	1.53	0.0068
Zfp867	zinc finger protein 867	1.53	0.0411
Sepw1	selenoprotein W, 1 (Sepw1), mRNA	1.53	0.0089
Crh	corticotropin releasing hormone	1.53	0.0232
Zfp951	zinc finger protein 951	1.53	0.0018
Sult1a1	sulfotransferase family 1A member 1	1.53	0.0390
RGD1311946	similar to RIKEN cDNA 1810055G02	1.53	0.0120
Slc30a1	solute carrier family 30 (zinc transporter), member 1	1.53	0.0036
LOC102555406	zinc finger protein 120-like	1.53	0.0130
Hba2	hemoglobin, alpha 2	1.53	0.0054
Arl6ip5	ADP-ribosylation factor-like 6 interacting protein 5	1.53	0.0160
Ppm1j	protein phosphatase, Mg2+/Mn2+ dependent, 1J	1.53	0.0097
LOC501033	similar to UPF0258 protein KIAA1024	1.53	0.0028
Sytl2	synaptotagmin-like 2 [Source:RGD Symbol;Acc:1307647]	1.53	0.0050
Sptbn1	spectrin, beta, non-erythrocytic 1	1.53	0.0006
LOC100911874	solute carrier family 40 member 1-like [Source:RGD Symbol;Acc:6490105]	1.52	0.0039
Il1rn	interleukin 1 receptor antagonist	1.52	0.0170
Pck2	phosphoenolpyruvate carboxykinase 2 (mitochondrial)	1.52	0.0010
Smim12	small integral membrane protein 12	1.52	0.0041
Olr1262	olfactory receptor 1262	1.52	0.0334
Evc	Ellis van Creveld protein	1.52	0.0003
Cmpk1	cytidine monophosphate (UMP-CMP) kinase 1	1.52	0.0005
Capn2	calpain 2, (m/II) large subunit	1.52	0.0160
Ear11	eosinophil-associated, ribonuclease A family, member 11 (Ear11), mRNA	1.52	0.0382
Tmem101	transmembrane protein 101	1.52	0.0304
Iah1	isoamyl acetate-hydrolyzing esterase 1 homolog	1.52	0.0126
Slc40a1	solute carrier family 40 (iron-regulated transporter), member 1	1.52	0.0182
Iqsec2	IQ motif and Sec7 domain 2	1.51	0.0075
Vom2r75	vomeronasal 2 receptor, 75	1.51	0.0272
Ptgfr	prostaglandin F receptor	1.51	0.0081
Schip1	schwannomin interacting protein 1	1.51	0.0372
Bcs1l	BC1 (ubiquinol-cytochrome c reductase) synthesis-like	1.51	0.0036
Acad11	acyl-CoA dehydrogenase family, member 11	1.51	0.0181
Olr436	olfactory receptor 436	1.51	0.0451
LOC102552920	armadillo repeat-containing X-linked protein 5-like	1.51	0.0151
Cpt2	carnitine palmitoyltransferase 2	1.51	0.0055
S100g	S100 calcium binding protein G	1.51	0.0235
Ltbp4	latent transforming growth factor beta binding protein 4	1.51	0.0031
Ppox	protoporphyrinogen oxidase	1.51	0.0300
Myef2	myelin expression factor 2	1.51	0.0235
Csmd1	CUB and Sushi multiple domains 1	1.51	0.0408

Pf4	platelet factor 4	1.51	0.0420
Tmem167a	transmembrane protein 167A	1.50	0.0003
Coq7	coenzyme Q7 homolog, ubiquinone (yeast)	1.50	0.0050
Syt6	synaptotagmin VI	1.50	0.0000
Psmb8	ENCODES a protein that exhibits threonine-type endopeptidase activity (inferred)	1.50	0.0028
Tas2r140	taste receptor, type 2, member 140	1.50	0.0168
Tecr	trans-2,3-enoyl-CoA reductase	1.50	0.0008
Nckap5	NCK-associated protein 5	1.50	0.0256
Arf2	ADP-ribosylation factor 2	1.50	0.0211
Arhgap24	Rho GTPase activating protein 24	1.50	0.0060
Plgrkt	plasminogen receptor, C-terminal lysine transmembrane protein	1.50	0.0435
Tmem120b	transmembrane protein 120B	1.50	0.0032
Mras	muscle RAS oncogene homolog	1.50	0.0261
Thnsl2	threonine synthase-like 2 (S. cerevisiae)	1.50	0.0061
Tagln2	transgelin 2	1.50	0.0093
Pdgfd	platelet derived growth factor D	1.50	0.0337
Gadl1	glutamate decarboxylase-like 1	1.50	0.0146
Man2b2	mannosidase, alpha, class 2B, member 2	1.49	0.0082
Mapre2	microtubule-associated protein, RP/EB family, member 2	1.49	0.0410
Msl3l2	male-specific lethal 3-like 2 (Drosophila)	1.49	0.0090
LOC100911718	cytochrome P450 2C6-like	1.49	0.0006
Garnl3	ENCODES a protein that exhibits GTPase activator activity (inferred)	1.49	0.0132
Chuk	conserved helix-loop-helix ubiquitous kinase	1.49	0.0005
Vom2r44	vomeronasal 2 receptor 44	1.49	0.0394
Nexn	nexilin (F actin binding protein)	1.49	0.0289
RGD1566085	similar to pyridoxal (pyridoxine, vitamin B6) kinase [Source:RGD Symbol;Acc:1566085]	1.49	0.0002
2510049J12Rik	RIKEN cDNA 2510049J12 gene	1.49	0.0484
Tcn2	transcobalamin 2	1.49	0.0401
Sptb	spectrin, beta, erythrocytic	1.49	0.0235
Cdk17	cyclin-dependent kinase 17	1.49	0.0388
Vom2r1	vomeronasal 2 receptor, 1	1.49	0.0475
Ccdc171	coiled-coil domain containing 171	1.49	0.0049
Ctsd	cathepsin D	1.49	0.0108
Cryz	crystallin, zeta (quinone reductase)	1.49	0.0173
Tcf7l1	transcription factor 7-like 1 (T-cell specific, HMG-box)	1.49	0.0372
LOC100294508	dyslexia susceptibility 2-like	1.49	0.0006
Atf7ip	activating transcription factor 7 interacting protein	1.49	0.0219
Acvrl1	activin A receptor type II-like 1	1.48	0.0001
Agap3	arf-GAP with GTPase, ANK repeat and PH domain-containing protein 3 [Source:RefSeq peptide;Acc:NP_001102086]	1.48	0.0281
Hsdl2	hydroxysteroid dehydrogenase like 2	1.48	0.0172
Tpm4	tropomyosin 4	1.48	0.0253
Pigk	phosphatidylinositol glycan anchor biosynthesis, class K	1.48	0.0050
Sema3c	sema domain, immunoglobulin domain (Ig), short basic domain, secreted, (semaphorin) 3C	1.48	0.0081
---	Uncharacterized protein	1.48	0.0199
Vcl	vinculin	1.48	0.0028
LOC367597	similar to nidogen 2	1.48	0.0142
RGD1305587	similar to RIKEN cDNA 2010107G23	1.48	0.0118
Vsx1	visual system homeobox 1	1.48	0.0218
Entpd8	ectonucleoside triphosphate diphosphohydrolase 8	1.48	0.0151
Abcc3	ATP-binding cassette, subfamily C (CFTR/MRP), member 3	1.48	0.0344
Prkca	protein kinase C, alpha	1.48	0.0067
Chpf	chondroitin polymerizing factor	1.48	0.0005
Prelid1	PRELI domain containing 1	1.48	0.0003
Peg3	paternally expressed 3	1.48	0.0049

Hist2h3c2	histone cluster 2, H3c2	1.48	0.0475
Specc1	sperm antigen with calponin homology and coiled-coil domains 1	1.48	0.0052
Mogat3	monoacylglycerol O-acyltransferase 3	1.48	0.0240
RGD1560015	INTERACTS WITH ammonium chloride	1.48	0.0282
Slc9a3r2	solute carrier family 9, subfamily A (NHE3, cation proton antiporter 3), member 3 regulator 2	1.48	0.0039
Acat1	acetyl-CoA acetyltransferase 1	1.47	0.0032
Dnaja4	DnaJ (Hsp40) homolog, subfamily A, member 4	1.47	0.0206
Sorcs1	sortilin-related VPS10 domain containing receptor 1	1.47	0.0037
Ednra	endothelin receptor type A	1.47	0.0036
Sgcb	sarcoglycan, beta	1.47	0.0191
Cfl1	cofilin 1, non-muscle	1.47	0.0027
LOC681177	similar to GTPase activating protein testicular GAP1	1.47	0.0159
Olr1391	olfactory receptor 1391	1.47	0.0293
Bloc1s5	biogenesis of lysosomal organelles complex-1, subunit 5, muted	1.47	0.0034
LOC100911860	ENCODES a protein that exhibits cysteine-type peptidase activity (inferred)	1.47	0.0088
Hadha	hydroxyacyl-CoA dehydrogenase/3-ketoacyl-CoA thiolase/enoyl-CoA hydratase (trifunctional protein), alpha subunit	1.47	0.0002
Commd9	COMM domain containing 9	1.47	0.0292
Satb1	SATB homeobox 1	1.47	0.0111
Emc1	ER membrane protein complex subunit 1	1.47	0.0449
Lpin2	lipin 2	1.47	0.0498
Olr377	olfactory receptor 377	1.47	0.0049
Slco6d1	solute carrier organic anion transporter family, member 6d1 [Source:RGD Symbol;Acc:1359388]	1.47	0.0160
Herc3	HECT and RLD domain containing E3 ubiquitin protein ligase 3	1.47	0.0016
Npy	neuropeptide Y	1.47	0.0048
Gnat3	guanine nucleotide binding protein, alpha transducing 3	1.47	0.0171
Wfdc3	WAP four-disulfide core domain 3	1.47	0.0473
Gnptab	N-acetylglucosamine-1-phosphate transferase, alpha and beta subunits	1.47	0.0175
Ap3s2	adaptor-related protein complex 3, sigma 2 subunit	1.46	0.0007
Carf	calcium responsive transcription factor	1.46	0.0374
Bahd1	bromo adjacent homology domain containing 1	1.46	0.0171
Tmem126a	transmembrane protein 126A	1.46	0.0006
Kif21a	kinesin family member 21A	1.46	0.0009
Arpp19	cAMP-regulated phosphoprotein 19 (Arpp19), mRNA	1.46	0.0130
Zfp36l2	zinc finger protein 36, C3H type-like 2	1.46	0.0297
Clcn2	chloride channel, voltage-sensitive 2	1.46	0.0185
Osgepl1	O-sialoglycoprotein endopeptidase-like 1	1.46	0.0214
Tp53inp1	tumor protein p53 inducible nuclear protein 1	1.46	0.0183
Leap2	liver-expressed antimicrobial peptide 2	1.46	0.0322
Abcg3l1	ATP-binding cassette, subfamily G (WHITE), member 3-like 1	1.46	0.0048
Armcx6	armadillo repeat containing, X-linked 6	1.46	0.0088
Zfp36l2	zinc finger protein 36, C3H type-like 2	1.46	0.0175
Fam114a1	family with sequence similarity 114, member A1	1.46	0.0304
Pamr1	peptidase domain containing associated with muscle regeneration 1	1.46	0.0071
Gtf2h2	general transcription factor IIH, polypeptide 2	1.46	0.0198
Phactr2	phosphatase and actin regulator 2	1.46	0.0299
Slc13a2	solute carrier family 13 (sodium-dependent dicarboxylate transporter), member 2	1.46	0.0384
Tpm3	tropomyosin 3	1.46	0.0008
Nlrp14	NLR family, pyrin domain containing 14	1.46	0.0082
B4galt6	UDP-Gal:betaGlcNAc beta 1,4-galactosyltransferase, polypeptide 6	1.46	0.0133
Fhl1	four and a half LIM domains 1	1.45	0.0183
Naprt	nicotinate phosphoribosyltransferase	1.45	0.0099
Synj2	synaptojanin 2	1.45	0.0333
Vim	vimentin	1.45	0.0001
Cpb1	carboxypeptidase B1 (tissue)	1.45	0.0290

Pcdhgb4	protocadherin gamma subfamily B, 4	1.45	0.0147
Ppa1	pyrophosphatase (inorganic) 1	1.45	0.0097
Cd24	CD24 molecule	1.45	0.0258
Coq5	coenzyme Q5, methyltransferase	1.45	0.0125
Fads1	fatty acid desaturase 1	1.45	0.0243
Atp6v1e1	ATPase, H+ transporting, lysosomal V1 subunit E1	1.45	0.0025
Tlr3	toll-like receptor 3	1.45	0.0097
Lama2	laminin, alpha 2	1.45	0.0031
LOC102551184	INTERACTS WITH arsenite(3-) (ortholog) AND cyclosporin A (ortholog) AND diarsenic trioxide (ortholog)	1.45	0.0010
Tex13	testis expressed gene 13	1.45	0.0039
Cbr1	carbonyl reductase 1	1.45	0.0399
Laptm5	lysosomal protein transmembrane 5	1.45	0.0106
Lyar	Ly1 antibody reactive	1.45	0.0070
RGD1308195	similar to secreted Ly6/uPAR related protein 2	1.45	0.0062
Acp5	acid phosphatase 5, tartrate resistant	1.45	0.0118
Trip11	thyroid hormone receptor interactor 11	1.45	0.0135
Bckdhb	branched chain keto acid dehydrogenase E1, beta polypeptide	1.45	0.0004
LOC100909622	INTERACTS WITH (-)-epigallocatechin 3-gallate (ortholog) AND aflatoxin B1 (ortholog) AND cyclosporin A (ortholog)	1.45	0.0177
Stau2	staufen double-stranded RNA binding protein 2	1.45	0.0126
LOC102547118	retinitis pigmentosa 1-like 1 protein-like [Source:RGD Symbol;Acc:7681336]	1.45	0.0297
Gpx7	glutathione peroxidase 7	1.45	0.0375
Ces2g	carboxylesterase 2G	1.45	0.0418
Tprg1l	tumor protein p63 regulated 1-like	1.45	0.0054
Hrasls	HRAS-like suppressor	1.44	0.0115
Alox15	arachidonate 15-lipoxygenase	1.44	0.0209
LOC691670	similar to natural killer cell protease 7	1.44	0.0037
Ccdc152	coiled-coil domain containing 152	1.44	0.0307
Ccdc88c	coiled-coil domain containing 88C	1.44	0.0283
Cox10	COX10 heme A:farnesyltransferase cytochrome c oxidase assembly factor	1.44	0.0252
Jph4	junctophilin 4	1.44	0.0060
LOC100910833	histone-lysine N-methyltransferase setd3-like	1.44	0.0029
Scamp5	secretory carrier membrane protein 5	1.44	0.0325
Ccnb2	cyclin B2	1.44	0.0435
Faap24	Fanconi anemia core complex associated protein 24	1.44	0.0229
Olr372	olfactory receptor 372	1.44	0.0237
Oprm1	opioid receptor, mu 1	1.44	0.0011
LOC100909786	INTERACTS WITH 17beta-hydroxy-17-methylestra-4 9 11-trien-3-one (ortholog)	1.44	0.0081
RGD1560273	RGD1560273	1.44	0.0251
LOC100910708	INVOLVED IN oxidation-reduction process (inferred) AND INTERACTS WITH (R)-acetoin (ortholog)	1.44	0.0359
Tpgs2	tubulin polyglutamylase complex subunit 2	1.44	0.0027
Ndufa8	NADH dehydrogenase (ubiquinone) 1 alpha subcomplex, 8	1.44	0.0134
Miox	myo-inositol oxygenase	1.44	0.0022
Mageb7	melanoma antigen, family B, 7	1.44	0.0379
Ift122	intraflagellar transport 122	1.44	0.0156
Etl4	Protein Etl4	1.43	0.0299
LOC691272	similar to reproductive homeobox on X chromosome, 11	1.43	0.0454
Cttnbp2nl	CTTNBP2 N-terminal like	1.43	0.0170
Cygb	cytoglobin	1.43	0.0357
Cst3	cystatin C	1.43	0.0128
Wisp2	WNT1 inducible signaling pathway protein 2	1.43	0.0051
LOC100361993	transmembrane protein 14C [Source:RGD Symbol;Acc:2319087]	1.43	0.0185
Cetn2	Centrin 2, isoform CRA_a; Protein Cetn2	1.43	0.0322
Nt5dc2	5-nucleotidase domain containing 2	1.43	0.0064
GSTA1	Glutathione S-transferase	1.43	0.0275

Efemp2	EGF-containing fibulin-like extracellular matrix protein 2	1.43	0.0248
Gapdh-ps2	glyceraldehyde-3-phosphate dehydrogenase, pseudogene 2	1.43	0.0005
---	Uncharacterized protein	1.43	0.0324
Asrgl1	asparaginase like 1	1.43	0.0370
LOC100174910	glutaredoxin-like protein	1.43	0.0370
Commd8	COMM domain containing 8	1.43	0.0093
Syn3	synapsin III	1.43	0.0052
P4hb	prolyl 4-hydroxylase, beta polypeptide	1.43	0.0023
Ctgf	connective tissue growth factor	1.43	0.0442
Eppk1	ENCODES a protein that exhibits poly(A) RNA binding (ortholog)	1.43	0.0093
Hist1h4b	histone cluster 1, H4b	1.43	0.0223
Dtnb	dystrobrevin, beta	1.43	0.0013
Cdc25b	cell division cycle 25B	1.43	0.0290
Kif1c	kinesin family member 1C	1.43	0.0250
Cd151	CD151 molecule (Raph blood group)	1.43	0.0006
RT1-N2	RT1 class Ib, locus N2	1.43	0.0024
Decr1	2,4-dienoyl CoA reductase 1, mitochondrial	1.42	0.0167
Atp1b3	ATPase, Na+/K+ transporting, beta 3 polypeptide	1.42	0.0010
Fam20b	family with sequence similarity 20, member B	1.42	0.0222
Serpinb6	serpin peptidase inhibitor, clade B (ovalbumin), member 6	1.42	0.0098
Trpm2	transient receptor potential cation channel, subfamily M, member 2	1.42	0.0172
Ogdhl	oxoglutarate dehydrogenase-like	1.42	0.0012
Dlst	dihydrolipoamide S-succinyltransferase (E2 component of 2-oxo-glutarate complex)	1.42	0.0001
Pfkl	phosphofructokinase, liver	1.42	0.0088
Sec13	SEC13 homolog, nuclear pore and COPII coat complex component	1.42	0.0184
Xrcc6	X-ray repair complementing defective repair in Chinese hamster cells 6	1.42	0.0151
Rbm48	RNA binding motif protein 48	1.42	0.0012
Pbsn	probasin	1.42	0.0035
Rcl1	RNA terminal phosphate cyclase-like 1	1.42	0.0211
Abhd12	abhydrolase domain containing 12	1.42	0.0449
Arl6	ADP-ribosylation factor-like 6	1.42	0.0143
Ssbp3	single stranded DNA binding protein 3	1.42	0.0038
RGD1564138	similar to 60S ribosomal protein L29 (P23) [Source:RGD Symbol;Acc:1564138]	1.42	0.0129
Ccnb1ip1	cyclin B1 interacting protein 1, E3 ubiquitin protein ligase	1.42	0.0326
Pip4k2b	phosphatidylinositol-5-phosphate 4-kinase, type II, beta	1.42	0.0184
Mad2l2	MAD2 mitotic arrest deficient-like 2 (yeast)	1.42	0.0242
Nr1h5	nuclear receptor subfamily 1, group H, member 5	1.42	0.0015
Nmi	N-myc (and STAT) interactor	1.42	0.0089
Tmem70	transmembrane protein 70	1.42	0.0337
Skp1	S-phase kinase-associated protein 1	1.42	0.0045
Trafd1	TRAF type zinc finger domain containing 1	1.42	0.0105
Ngfrap1	nerve growth factor receptor (TNFRSF16) associated protein 1	1.42	0.0425
LOC100912836	leucine-rich repeat-containing protein PRAME-like [Source:RGD Symbol;Acc:6485636]	1.42	0.0163
Kng1	kininogen 1kininogen 2	1.42	0.0031
Carhsp1	calcium regulated heat stable protein 1	1.42	0.0166
Fbxo43	F-box protein 43	1.41	0.0188
Bsg	basigin (Ok blood group)	1.41	0.0134
Sec24d	SEC24 homolog D, COPII coat complex component	1.41	0.0057
LOC100360061	INTERACTS WITH 17beta-hydroxy-17-methylestra-4 9 11-trien-3-one (ortholog)	1.41	0.0438
Pik3r3	phosphoinositide-3-kinase, regulatory subunit 3 (gamma)	1.41	0.0194
Mycl	v-myc avian myelocytomatosis viral oncogene lung carcinoma derived homolog	1.41	0.0389
Pdc	phosducin	1.41	0.0329
Bmp10	bone morphogenetic protein 10	1.41	0.0081

Zfp541	zinc finger protein 541	1.41	0.0278
Zfp52	zinc finger protein 52	1.41	0.0082
Sult2b1	sulfotransferase family 2B member 1	1.41	0.0189
Syngap1	synaptic Ras GTPase activating protein 1	1.41	0.0056
Ppp2ca	protein phosphatase 2, catalytic subunit, alpha isozyme	1.41	0.0025
Bin3	bridging integrator 3	1.41	0.0020
LOC681736	similar to Glycolipid transfer protein (GLTP)	1.41	0.0171
Sec22b	SEC22 vesicle trafficking protein homolog B (S. cerevisiae)	1.41	0.0018
LOC501339	similar to spermatogenesis associated glutamate (E)-rich protein 4dsimilar to zinc finger CCCH type, antiviral 1	1.41	0.0281
Cbx2	chromobox homolog 2	1.41	0.0022
Tpi1	triosephosphate isomerase 1	1.41	0.0216
LOC100909867	epididymal-specific lipocalin-13-like [Source:RGD Symbol;Acc:6501236]	1.41	0.0322
Nlrx1	NLR family member X1	1.41	0.0242
Gm14569	predicted gene 14569 [Source:MGI Symbol;Acc:MGI:3705243]	1.41	0.0375
Olr1145	olfactory receptor 1145	1.41	0.0151
Pgm1	phosphoglucomutase 1	1.41	0.0006
Scd4	stearoyl-coenzyme A desaturase 4	1.40	0.0432
Hsd17b13	hydroxysteroid (17-beta) dehydrogenase 13	1.40	0.0097
Ehd4	EH-domain containing 4	1.40	0.0231
Lgmn	legumain	1.40	0.0243
Cfap126	cilia and flagella associated protein 126	1.40	0.0353
Col1a2	collagen, type I, alpha 2 (Col1a2), mRNA	1.40	0.0371
Tmem41a	transmembrane protein 41a	1.40	0.0202
Acad9	acyl-CoA dehydrogenase family, member 9	1.40	0.0007
Pot1	protection of telomeres 1	1.40	0.0037
Mtmr9	myotubularin related protein 9	1.40	0.0493
LOC100360238	rCG21419-like	1.40	0.0225
RGD1565367	similar to Solute carrier family 23, member 2 (Sodium-dependent vitamin C transporter 2)	1.40	0.0385
Eif2b2	eukaryotic translation initiation factor 2B, subunit 2 beta	1.40	0.0027
Cd82	Cd82 molecule	1.40	0.0095
Ndst4	N-deacetylase/N-sulfotransferase (heparan glucosaminyl) 4	1.40	0.0118
Mxd4	Max dimerization protein 4	1.40	0.0072
LOC679149	similar to carboxylesterase 2 (intestine, liver)	1.40	0.0225
Mesdc2	mesoderm development candidate 2	1.40	0.0027
Asah1	N-acylsphingosine amidohydrolase (acid ceramidase) 1	1.40	0.0042
Akr1c14	aldo-keto reductase family 1, member C14	1.40	0.0297
Gapdh	glyceraldehyde-3-phosphate dehydrogenase	1.40	0.0001
LOC100909548	INTERACTS WITH chloroquine AND diuron AND 17alpha-ethynylestradiol (ortholog)	1.40	0.0003
LOC102554321	uncharacterized LOC102554321	1.40	0.0126
RGD1564836	similar to HMGA1b	1.40	0.0187
Tmem14a	transmembrane protein 14A	1.40	0.0347
Bcr	breakpoint cluster region [Source:RGD Symbol;Acc:1307993]	1.40	0.0383
Rnase17	ribonuclease 17	1.39	0.0400
Tgoln2	trans-golgi network protein 2	1.39	0.0235
Mcam	melanoma cell adhesion molecule	1.39	0.0370
Slc39a10	solute carrier family 39 (zinc transporter), member 10	1.39	0.0298
Hist1h1b	histone cluster 1, H1b	1.39	0.0243
NONMMUG026947	Non-coding transcript identified by NONCODE: Exonic	1.39	0.0206
Adh5	alcohol dehydrogenase 5 (class III), chi polypeptide	1.39	0.0317
Pde5a	phosphodiesterase 5A, cGMP-specific	1.39	0.0342
Hist1h4b	histone cluster 1, H4b [Source:RGD Symbol;Acc:620814]	1.39	0.0228
Cd151	CD151 molecule (Raph blood group) (Cd151), mRNA	1.39	0.0028
Atg4a	autophagy related 4A, cysteine peptidase	1.39	0.0349
Pdxk	pyridoxal (pyridoxine, vitamin B6) kinase	1.39	0.0143

Ackr3	atypical chemokine receptor 3	1.39	0.0193
Comt	catechol-O-methyltransferase	1.39	0.0039
Galt	galactose-1-phosphate uridylyltransferase	1.39	0.0067
Ybx3	Y box binding protein 3	1.39	0.0156
Epha7	Eph receptor A7	1.39	0.0092
Trim65	tripartite motif-containing 65	1.39	0.0157
Hscb	HscB mitochondrial iron-sulfur cluster co-chaperone	1.39	0.0112
Ippk	inositol 1,3,4,5,6-pentakisphosphate 2-kinase	1.39	0.0340
Rnpep	arginyl aminopeptidase (aminopeptidase B)	1.39	0.0107
Icmt	isoprenylcysteine carboxyl methyltransferase	1.39	0.0033
Nudt13	nudix (nucleoside diphosphate linked moiety X)-type motif 13	1.39	0.0340
Olr1107	olfactory receptor 1107	1.39	0.0320
Acy1	aminoacylase 1	1.39	0.0364
NONMMUG023236	Non-coding transcript identified by NONCODE: Sense No Exonic	1.39	0.0192
Cybb	cytochrome b-245, beta polypeptide	1.39	0.0025
Dgkz	diacylglycerol kinase zeta	1.39	0.0394
Npy	neuropeptide Y (Npy), mRNA	1.39	0.0095
Tmem25	transmembrane protein 25	1.39	0.0120
Tmprss6	transmembrane protease, serine 6	1.39	0.0227
Mfrp	membrane frizzled-related protein	1.39	0.0493
Olr853	olfactory receptor 853	1.39	0.0380
Nkx2-4	NK2 homeobox 4	1.38	0.0005
Pacsin2	protein kinase C and casein kinase substrate in neurons 2	1.38	0.0204
Sparcl1	SPARC-like 1 (hevin)	1.38	0.0287
Hsd17b12	hydroxysteroid (17-beta) dehydrogenase 12	1.38	0.0042
Cdrt4	CMT1A duplicated region transcript 4	1.38	0.0499
Mrpl37	mitochondrial ribosomal protein L37	1.38	0.0043
Lilrb3	leukocyte immunoglobulin-like receptor, subfamily B (with TM and ITIM domains), member 3	1.38	0.0429
Aldoart2	aldolase 1 A retrogene 2	1.38	0.0179
Flna	filamin A, alpha	1.38	0.0252
LOC100911477	nidogen-2-like	1.38	0.0217
Ttc36	tetratricopeptide repeat domain 36	1.38	0.0235
Snx7	sorting nexin 7	1.38	0.0337
Cers5	ceramide synthase 5	1.38	0.0190
Kpna2	karyopherin alpha 2	1.38	0.0339
Mbd2	methyl-CpG binding domain protein 2	1.38	0.0007
LOC102555146	fatty acid-binding protein epidermal-like	1.38	0.0049
Slc25a1	solute carrier family 25 (mitochondrial carrier, citrate transporter), member 1	1.38	0.0024
LOC684841	ENCODES a protein that exhibits DNA binding (inferred)	1.38	0.0089
Fam159b	family with sequence similarity 159, member B [Source:RGD Symbol;Acc:1564672]	1.38	0.0465
Cnpy1	canopy FGF signaling regulator 1 [Source:RGD Symbol;Acc:1583296]	1.38	0.0021
Ccna2	cyclin A2	1.37	0.0234
Zfp511	zinc finger protein 511	1.37	0.0085
Gsta1	glutathione S-transferase alpha 1	1.37	0.0313
Fnbp1l	formin binding protein 1-like	1.37	0.0210
Lpgat1	lysophosphatidylglycerol acyltransferase 1	1.37	0.0044
Slitrk6	SLIT and NTRK-like family, member 6	1.37	0.0329
Mrpl13	mitochondrial ribosomal protein L13	1.37	0.0382
Plau	plasminogen activator, urokinase	1.37	0.0317
Tp53i3	tumor protein p53 inducible protein 3	1.37	0.0085
Odc1	ornithine decarboxylase 1	1.37	0.0020
Papss1	3-phosphoadenosine 5-phosphosulfate synthase 1	1.37	0.0369
Acaa1b	acetyl-Coenzyme A acyltransferase 1B	1.37	0.0088
Sucla2	succinate-CoA ligase, ADP-forming, beta subunit	1.37	0.0022

Sowahc	sosondowah ankyrin repeat domain family member C	1.37	0.0036
Mvd	mevalonate (diphospho) decarboxylase	1.37	0.0413
Mmp27	matrix metallopeptidase 27	1.37	0.0348
Macrod1	MACRO domain containing 1	1.37	0.0283
Zfp7	zinc finger protein 7	1.37	0.0250
Fam213b	family with sequence similarity 213, member B	1.37	0.0045
Fto	fat mass and obesity associated (Fto), mRNA.	1.37	0.0424
Lilra5	leukocyte immunoglobulin-like receptor, subfamily A (with TM domain), member 5	1.37	0.0022
Sgcg	sarcoglycan, gamma	1.37	0.0092
Map4k2	mitogen activated protein kinase kinase kinase kinase 2	1.37	0.0455
LOC303448	similar to glyceraldehyde-3-phosphate dehydrogenase	1.37	0.0085
Tes	testin LIM domain protein	1.37	0.0232
RGD1562690	PARTICIPATES IN cysteine and methionine metabolic pathway AND gluconeogenesis pathway AND glycolysis pathway	1.37	0.0004
Dnajc19	DnaJ (Hsp40) homolog, subfamily C, member 19	1.36	0.0062
Snx21	---	1.36	0.0130
LOC500684	hypothetical protein LOC500684	1.36	0.0019
Col14a1	collagen, type XIV, alpha 1	1.36	0.0375
LOC684475	similar to tumor protein p53 inducible protein 3	1.36	0.0322
Letmd1	LETM1 domain containing 1	1.36	0.0113
Sirpb2l1	INTERACTS WITH 17beta-estradiol (ortholog)	1.36	0.0138
Mafk	v-maf avian musculoaponeurotic fibrosarcoma oncogene homolog K	1.36	0.0412
Srp14	signal recognition particle 14	1.36	0.0197
Suclg1	succinate-CoA ligase, alpha subunit	1.36	0.0082
Mppe1	metallophosphoesterase 1	1.36	0.0327
04. Sep	septin 4	1.36	0.0325
---	Uncharacterized protein	1.36	0.0287
---	Uncharacterized protein	1.36	0.0446
P4ha3	prolyl 4-hydroxylase, alpha polypeptide III	1.36	0.0203
Sardh	sarcosine dehydrogenase	1.36	0.0366
Krt23	keratin 23, type I	1.36	0.0496
Otub1	OTU deubiquitinase, ubiquitin aldehyde binding 1	1.36	0.0164
Krt8	keratin 8, type II	1.36	0.0483
Olr453	olfactory receptor 453	1.36	0.0350
Aga	aspartylglucosaminidase	1.36	0.0053
Tas1r3	taste receptor, type 1, member 3	1.36	0.0259
Arl2	ADP-ribosylation factor-like 2	1.36	0.0374
Eps15l1	ENCODES a protein that exhibits calcium ion binding (inferred)	1.36	0.0079
Tpm3	tropomyosin 3	1.36	0.0128
Glg1	golgi glycoprotein 1	1.36	0.0248
Efnb2	ephrin B2	1.36	0.0479
Pde4a	phosphodiesterase 4A, cAMP-specific	1.36	0.0098
LOC501230	similar to nidogen 2	1.36	0.0050
Psph	phosphoserine phosphatase	1.36	0.0301
Pcdhgb5	protocadherin gamma subfamily B, 5	1.36	0.0373
Kifc1	kinesin family member C1	1.36	0.0122
Adam23	ADAM metallopeptidase domain 23	1.36	0.0248
Snx5	sorting nexin 5	1.35	0.0170
Aco2	aconitase 2, mitochondrial	1.35	0.0337
Veph1	ventricular zone expressed PH domain-containing 1	1.35	0.0239
Gpx1	glutathione peroxidase 1	1.35	0.0039
Tp53i11	tumor protein p53 inducible protein 11	1.35	0.0301
Ldha	lactate dehydrogenase A	1.35	0.0013
Sub1	SUB1 homolog, transcriptional regulator	1.35	0.0118
Etfa	electron-transfer-flavoprotein, alpha polypeptide	1.35	0.0171
Cd63	Cd63 molecule	1.35	0.0494
Cotl1	coactosin-like F-actin binding protein 1	1.35	0.0273

Lamp3	lysosomal-associated membrane protein 3	1.35	0.0039
Cd99l2	CD99 molecule-like 2 [Source:RGD Symbol;Acc:620896]	1.35	0.0182
LOC102553500	uncharacterized LOC102553500	1.35	0.0003
Acaa2	acetyl-CoA acyltransferase 2	1.35	0.0123
Flvcr2	feline leukemia virus subgroup C cellular receptor family, member 2	1.35	0.0261
Prdm16	PR domain containing 16	1.35	0.0181
Spata7	spermatogenesis associated 7	1.35	0.0254
Snx9	sorting nexin 9	1.35	0.0262
Atp5o	ATP synthase, H+ transporting, mitochondrial F1 complex, O subunit	1.35	0.0493
Bbox1	butyrobetaine (gamma), 2-oxoglutarate dioxygenase (gamma-butyrobetaine hydroxylase) 1	1.35	0.0356
Ank3	ankyrin 3, node of Ranvier (ankyrin G)	1.35	0.0085
Ano10	anoctamin 10	1.35	0.0413
Abracl	ABRA C-terminal like	1.35	0.0484
Ppp5c	protein phosphatase 5, catalytic subunit	1.35	0.0103
Polb	polymerase (DNA directed), beta	1.35	0.0109
Tstd3	thiosulfate sulfurtransferase (rhodanese)-like domain containing 3	1.35	0.0452
Cplx2	complexin 2	1.35	0.0498
Rfx5	regulatory factor X, 5 (influences HLA class II expression)	1.35	0.0384
Mien1	migration and invasion enhancer 1	1.35	0.0233
Atp6v1a	ATPase, H+ transporting, lysosomal V1 subunit A	1.35	0.0379
Tpsg1	tryptase gamma 1	1.35	0.0380
Psd2	pleckstrin and Sec7 domain containing 2	1.34	0.0355
Rgma	repulsive guidance molecule family member A	1.34	0.0336
Chchd6	coiled-coil-helix-coiled-coil-helix domain containing 6	1.34	0.0387
Zmat3	zinc finger, matrin type 3	1.34	0.0437
Ahnak	AHNAK nucleoprotein	1.34	0.0071
Rala	v-ral simian leukemia viral oncogene homolog A (ras related)	1.34	0.0009
Kif4a	kinesin family member 4A	1.34	0.0161
Ndufs5	NADH dehydrogenase (ubiquinone) Fe-S protein 5	1.34	0.0167
Strap	serine/threonine kinase receptor associated protein	1.34	0.0099
Prdm11	PR domain containing 11	1.34	0.0446
Mrpl1	mitochondrial ribosomal protein L1	1.34	0.0384
Rgs2	regulator of G-protein signaling 2	1.34	0.0317
Usp30	ubiquitin specific peptidase 30	1.34	0.0174
LOC688925	similar to Glutathione S-transferase alpha-4	1.34	0.0375
Olr1179	olfactory receptor 1179	1.34	0.0427
Cep57l1	centrosomal protein 57-like 1	1.34	0.0075
Rnf180	ring finger protein 180	1.34	0.0325
Actl9	actin-like 9	1.34	0.0315
Gyg1	glycogenin 1	1.34	0.0403
Hgf	hepatocyte growth factor	1.34	0.0188
LOC102549235	uncharacterized LOC102549235	1.34	0.0349
Gpr176	G protein-coupled receptor 176	1.34	0.0133
Pnmal2	paraneoplastic Ma antigen family-like 2	1.34	0.0408
Abi2	abl-interactor 2	1.34	0.0014
Kng1l1	kininogen 1-like 1	1.34	0.0148
Snap23	synaptosomal-associated protein 23	1.34	0.0217
Slc25a27	solute carrier family 25, member 27	1.34	0.0143
Atp5g1	ATP synthase, H+ transporting, mitochondrial Fo complex, subunit C1 (subunit 9)	1.34	0.0330
Sepw1	selenoprotein W, 1	1.34	0.0005
Cdc42se2	CDC42 small effector 2	1.34	0.0245
Tmem18	transmembrane protein 18	1.34	0.0144
Gcat	glycine C-acetyltransferase	1.34	0.0156
Ssty1	predicted gene, 21440	1.33	0.0077
RGD1559441	similar to MIC2L1 [Source:RGD Symbol;Acc:1559441]	1.33	0.0200
Irgq	immunity-related GTPase family, Q	1.33	0.0476

Aplp1	amyloid beta (A4) precursor-like protein 1	1.33	0.0465
Pgp	phosphoglycolate phosphatase	1.33	0.0109
Prdx6	peroxiredoxin 6	1.33	0.0334
Cdc42ep1	CDC42 effector protein (Rho GTPase binding) 1	1.33	0.0025
Grik1	glutamate receptor, ionotropic, kainate 1	1.33	0.0281
Fundc1	FUN14 domain containing 1	1.33	0.0413
Ubl3	ubiquitin-like 3	1.33	0.0021
Nol4l	nucleolar protein 4-like	1.33	0.0265
Lrrc75b	leucine rich repeat containing 75B	1.33	0.0499
LOC100910497	INTERACTS WITH (-)-demecolcine (ortholog)	1.33	0.0128
Aasdhppt	aminoadipate-semialdehyde dehydrogenase-phosphopantetheinyl transferase	1.33	0.0040
Arpp19	cAMP-regulated phosphoprotein 19 [Source:RGD Symbol;Acc:71054]	1.33	0.0364
Glipr2	GLI pathogenesis-related 2	1.33	0.0431
Olr1436	olfactory receptor 1436	1.33	0.0335
Olr1436	olfactory receptor 1436 (Olr1436), mRNA	1.33	0.0335
Blvra	biliverdin reductase A	1.33	0.0241
Lactb	lactamase, beta	1.33	0.0177
Arf3	ADP-ribosylation factor 3 (Arf3), mRNA	1.33	0.0413
Stoml1	stomatin (EPB72)-like 1	1.33	0.0112
Olr1311	olfactory receptor 1311	1.33	0.0305
Fam173b	family with sequence similarity 173, member B	1.33	0.0129
Slc34a1	solute carrier family 34 (type II sodium/phosphate cotransporter), member 1	1.33	0.0081
Pex19	peroxisomal biogenesis factor 19	1.33	0.0216
Fam96a	family with sequence similarity 96, member A	1.33	0.0363
Gfm2	G elongation factor, mitochondrial 2	1.33	0.0278
Plpp2	phospholipid phosphatase 2	1.33	0.0387
Akr1b10	aldo-keto reductase family 1, member B10 (aldose reductase)	1.33	0.0465
Fam91a1	family with sequence similarity 91, member A1	1.33	0.0387
Snf8	SNF8, ESCRT-II complex subunit	1.33	0.0102
RGD1563100	similar to KIAA0089	1.33	0.0349
Inpp4b	inositol polyphosphate-4-phosphatase, type II	1.33	0.0348
Ctxn2	cortexin 2	1.33	0.0374
Foxb1	forkhead box B1	1.33	0.0192
Nxnl1	nucleoredoxin-like 1	1.33	0.0243
Sugp2	SURP and G patch domain containing 2	1.33	0.0083
Rbm8a	RNA binding motif protein 8A	1.33	0.0315
LOC685700	hypothetical protein LOC685700	1.33	0.0355
Tdgf1	teratocarcinoma-derived growth factor 1	1.33	0.0474
LOC102552746	uncharacterized LOC102552746	1.33	0.0005
Psmd13	proteasome 26S subunit, non-ATPase 13	1.32	0.0079
Mtl5	metallothionein-like 5, testis-specific (tesmin)	1.32	0.0172
Itgb3bp	integrin beta 3 binding protein (beta3-endonexin)	1.32	0.0318
Timm50	translocase of inner mitochondrial membrane 50	1.32	0.0419
Gng12	guanine nucleotide binding protein (G protein), gamma 12	1.32	0.0073
Shroom3	shroom family member 3	1.32	0.0048
Smim19	small integral membrane protein 19	1.32	0.0335
Raet1l	retinoic acid early transcript 1L	1.32	0.0394
LOC316935	similar to Spetex-2D protein [Source:RGD Symbol;Acc:1586158]	1.32	0.0045
Tmem56	transmembrane protein 56	1.32	0.0038
Tsc22d1	TSC22 domain family, member 1	1.32	0.0392
LOC100363268	INTERACTS WITH 17alpha-ethynylestradiol (ortholog)	1.32	0.0266
Aurkb	aurora kinase B	1.32	0.0452
Cdc25c	cell division cycle 25C	1.32	0.0213
Ifitm2	interferon induced transmembrane protein 2	1.32	0.0443
Suv420h2	suppressor of variegation 4-20 homolog 2 (Drosophila)	1.32	0.0414
Cd209c	CD209c molecule [Source:RGD Symbol;Acc:1582956]	1.32	0.0137
Cyp2a1	cytochrome P450, family 2, subfamily a, polypeptide 1	1.32	0.0252

Rogdi	rogdi homolog	1.32	0.0070
Lypla1	lysophospholipase I	1.32	0.0015
Rwdd3	RWD domain containing 3	1.32	0.0062
Pilra	paired immunoglobin-like type 2 receptor alpha [Source:RGD Symbol;Acc:1562847]	1.32	0.0101
Mmp19	matrix metallopeptidase 19	1.32	0.0149
Chd9	chromodomain helicase DNA binding protein 9	1.32	0.0446
Entpd7	ectonucleoside triphosphate diphosphohydrolase 7	1.32	0.0468
Fgf6	fibroblast growth factor 6	1.32	0.0276
Ube2l3	ubiquitin-conjugating enzyme E2L 3	1.32	0.0161
Hmha1	histocompatibility (minor) HA-1	1.32	0.0416
Sox18	SRY (sex determining region Y)-box 18	1.32	0.0195
Gpm6a	glycoprotein m6a	1.32	0.0452
Mtdh	ENCODES a protein that exhibits double-stranded RNA binding (ortholog)	1.32	0.0045
---	Uncharacterized protein	1.32	0.0140
Bsph2	binder of sperm protein homolog 2	1.31	0.0041
Rgs20	regulator of G-protein signaling 20	1.31	0.0491
Mef2b	myocyte enhancer factor 2B	1.31	0.0139
Fam212a	family with sequence similarity 212, member A	1.31	0.0120
Upp1	uridine phosphorylase 1	1.31	0.0316
Cldn16	claudin 16	1.31	0.0163
Tfdp1	transcription factor Dp-1	1.31	0.0263
Samd9l	INVOLVED IN common myeloid progenitor cell proliferation (ortholog)	1.31	0.0335
Mnat1	MNAT CDK-activating kinase assembly factor 1	1.31	0.0238
Tspan7	tetraspanin 7	1.31	0.0045
Dgkd	diacylglycerol kinase, delta	1.31	0.0096
Ppp1r7	protein phosphatase 1, regulatory subunit 7	1.31	0.0188
Exo5	exonuclease 5	1.31	0.0092
Npap60	nuclear pore associated protein [Source:RGD Symbol;Acc:3191]	1.31	0.0042
LOC100362391	NADH dehydrogenase (ubiquinone) 1 beta subcomplex 3-like [Source:RGD Symbol;Acc:2318296]	1.31	0.0171
Ccdc137	coiled-coil domain containing 137	1.31	0.0149
Vom2r50	vomeronasal 2 receptor, 50	1.31	0.0324
Pgbd5	piggyBac transposable element derived 5	1.31	0.0079
Smtn	FOUND IN actin cytoskeleton (ortholog) AND nucleus (ortholog) AND INTERACTS WITH C60 fullerene AND cisplatin AND cocaine	1.31	0.0005
Msrb2	methionine sulfoxide reductase B2	1.31	0.0265
Col20a1	collagen, type XX, alpha 1	1.31	0.0047
Itih4	inter-alpha-trypsin inhibitor heavy chain family, member 4	1.31	0.0043
Ndufc1	NADH dehydrogenase (ubiquinone) 1, subcomplex unknown, 1 [Source:RGD Symbol;Acc:1586362]	1.31	0.0140
Dhfr	dihydrofolate reductase	1.31	0.0007
Mdh2	malate dehydrogenase 2, NAD (mitochondrial)	1.31	0.0331
Hist3h3	histone cluster 3, H3	1.31	0.0497
Tax1bp3	Tax1 (human T-cell leukemia virus type I) binding protein 3	1.31	0.0460
Adam4	a disintegrin and metalloprotease domain 4	1.31	0.0287
Trub1	TruB pseudouridine (psi) synthase family member 1	1.31	0.0409
Zfp14	ZFP14 zinc finger protein	1.31	0.0243
Slurp1	secreted Ly6/Plaur domain containing 1	1.31	0.0486
Wbscr22	Williams Beuren syndrome chromosome region 22	1.31	0.0427
Itgb5	integrin, beta 5	1.31	0.0209
Ralgps1	Ral GEF with PH domain and SH3 binding motif 1	1.31	0.0338
Tanc1	tetratricopeptide repeat, ankyrin repeat and coiled-coil containing 1	1.31	0.0106
Sipa1	signal-induced proliferation-associated 1	-1.31	0.0231
Ctbs	chitobiase, di-N-acetyl-	-1.31	0.0002
Aim1l	absent in melanoma 1-like	-1.31	0.0445
Prss54	protease, serine, 54	-1.31	0.0277
Rbl2	retinoblastoma-like 2	-1.31	0.0229

Usp36	ubiquitin specific peptidase 36	-1.31	0.0311
Ebna1bp2	EBNA1 binding protein 2	-1.31	0.0145
Cox11	cytochrome c oxidase assembly protein 11	-1.31	0.0432
Ralgapa1	Ral GTPase activating protein, alpha subunit 1 (catalytic)	-1.31	0.0075
Ptpn7	protein tyrosine phosphatase, non-receptor type 7	-1.31	0.0390
Cyp2d5	cytochrome P450, family 2, subfamily d, polypeptide 5	-1.31	0.0056
LOC100362690	rCG64164-like	-1.31	0.0430
Atrn	attractin	-1.31	0.0387
Arfip2	ADP-ribosylation factor interacting protein 2	-1.31	0.0054
LOC100360117	ribosomal protein L8-like	-1.31	0.0240
Stag1	stromal antigen 1	-1.31	0.0158
Pip5k1c	phosphatidylinositol-4-phosphate 5-kinase, type I, gamma	-1.31	0.0499
Olr1606	olfactory receptor 1606	-1.31	0.0336
Fn1	fibronectin 1	-1.31	0.0061
Smc1a	structural maintenance of chromosomes 1A	-1.31	0.0095
LOC100910475	uncharacterized LOC100910475	-1.31	0.0340
Rlim	ring finger protein, LIM domain interacting	-1.31	0.0386
Akap8	A kinase (PRKA) anchor protein 8	-1.31	0.0238
Olr1591	olfactory receptor 1591	-1.31	0.0089
Dnajc1	DnaJ (Hsp40) homolog, subfamily C, member 1	-1.31	0.0436
Clec10a	C-type lectin domain family 10, member A	-1.31	0.0146
Card6	caspase recruitment domain family, member 6	-1.31	0.0246
Rnf145	ring finger protein 145	-1.31	0.0477
Cyp2s1	cytochrome P450, family 2, subfamily s, polypeptide 1	-1.31	0.0196
Lysmd3	LysM, putative peptidoglycan-binding, domain containing 3	-1.31	0.0324
LOC100911887	zinc finger protein 569-like	-1.31	0.0446
Wipi2	WD repeat domain, phosphoinositide interacting 2	-1.31	0.0256
Tram1	translocation associated membrane protein 1	-1.31	0.0059
Lag3	lymphocyte-activation gene 3	-1.31	0.0453
Wdfy2	WD repeat and FYVE domain containing 2	-1.31	0.0328
Tnks2	tankyrase, TRF1-interacting ankyrin-related ADP-ribose polymerase 2 (Tnks2), mRNA	-1.31	0.0031
Ugdh	UDP-glucose 6-dehydrogenase	-1.31	0.0113
Zfp239	zinc finger protein 239 [Source:MGI Symbol;Acc:MGI:1306812]	-1.31	0.0176
Mpp6	membrane protein, palmitoylated 6 (MAGUK p55 subfamily member 6)	-1.32	0.0088
Tcp1	t-complex 1	-1.32	0.0000
Tas2r138	taste receptor, type 2, member 138	-1.32	0.0455
Plrg1	pleiotropic regulator 1	-1.32	0.0103
Proc	protein C	-1.32	0.0006
LOC102553533	DDB1- and CUL4-associated factor 5-like	-1.32	0.0113
Slamf6	SLAM family member 6	-1.32	0.0365
LOC685718	similar to tumor protein, translationally-controlled 1 [Source:RGD Symbol;Acc:1590250]	-1.32	0.0022
Hoxb3	homeo box B3	-1.32	0.0291
Hyal4	hyaluronoglucosaminidase 4	-1.32	0.0109
Vom2r23	vomeronasal 2 receptor, 23	-1.32	0.0439
LOC689217	similar to mRNA capping enzyme (HCE) (MCE1)	-1.32	0.0462
Efs	embryonal Fyn-associated substrate	-1.32	0.0190
Bst1	bone marrow stromal cell antigen 1	-1.32	0.0134
Mxra7	matrix-remodelling associated 7	-1.32	0.0030
Ccnd2	cyclin D2	-1.32	0.0160
Epx	eosinophil peroxidase	-1.32	0.0348
Eml1	echinoderm microtubule associated protein like 1	-1.32	0.0315
Polr2j	polymerase (RNA) II (DNA directed) polypeptide J	-1.32	0.0346
Dhx37	DEAH (Asp-Glu-Ala-His) box polypeptide 37	-1.32	0.0474
Rp9	retinitis pigmentosa 9	-1.32	0.0471
Map3k2	mitogen activated protein kinase kinase kinase 2	-1.32	0.0225
LOC102553866	zinc finger protein 764-like	-1.32	0.0034

Asb18	ankyrin repeat and SOCS box-containing 18	-1.32	0.0457
Crisp2	cysteine-rich secretory protein 2	-1.32	0.0172
Ccdc109b	coiled-coil domain containing 109B	-1.32	0.0461
Faap100	Fanconi anemia core complex associated protein 100	-1.32	0.0445
Kansl2	KAT8 regulatory NSL complex subunit 2	-1.32	0.0023
Klk4	kallikrein-related peptidase 4	-1.32	0.0079
RGD1564883	ENCODES a protein that exhibits structural constituent of ribosome (inferred)	-1.32	0.0440
Tgm4	transglutaminase 4	-1.32	0.0196
Nlrp1a	NLR family, pyrin domain containing 1A	-1.32	0.0329
Lcorl	ligand dependent nuclear receptor corepressor-like	-1.32	0.0027
Arhgef10l	Rho guanine nucleotide exchange factor (GEF) 10-like [Source:RGD Symbol;Acc:1585585]	-1.32	0.0028
Gtpbp4	GTP binding protein 4	-1.32	0.0270
Nrxn3	neurexin 3	-1.32	0.0012
Senp7	SUMO1/sentrin specific peptidase 7	-1.32	0.0342
Zfp318	zinc finger protein 318	-1.32	0.0354
Kdm1a	lysine (K)-specific demethylase 1A	-1.32	0.0237
Nup98	nucleoporin 98	-1.33	0.0001
Pigv	phosphatidylinositol glycan anchor biosynthesis, class V	-1.33	0.0369
Clic5	chloride intracellular channel 5	-1.33	0.0088
Olr1733	olfactory receptor 1733	-1.33	0.0298
Otud4	OTU deubiquitinase 4	-1.33	0.0100
Arhgef18	rho/rac guanine nucleotide exchange factor (GEF) 18	-1.33	0.0282
Dcaf5	DDB1 and CUL4 associated factor 5	-1.33	0.0102
Zfp84	zinc finger protein 84	-1.33	0.0134
Zmynd10	zinc finger, MYND-type containing 10	-1.33	0.0322
LOC100911260	host cell factor 1-like	-1.33	0.0264
LOC102550668	INTERACTS WITH cisplatin (ortholog) AND cobalt dichloride (ortholog) AND copper(2+) sulfate (ortholog)	-1.33	0.0122
LOC102546322	COPII coat assembly protein sec16-like	-1.33	0.0159
Cadps2	Ca++-dependent secretion activator 2	-1.33	0.0010
Tbpl1	TBP-like 1	-1.33	0.0012
Sox21	ENCODES a protein that exhibits DNA binding (ortholog)	-1.33	0.0108
Pou2f2	POU class 2 homeobox 2	-1.33	0.0384
Tmem38b	transmembrane protein 38B	-1.33	0.0285
Plxnb2	plexin B2	-1.33	0.0337
Olr1451	ENCODES a protein that exhibits olfactory receptor activity (inferred)	-1.33	0.0081
St6galnac6	ST6 (alpha-N-acetyl-neuraminyl-2,3-beta-galactosyl-1,3)-N-acetylgalactosaminide alpha-2,6-sialyltransferase 6	-1.33	0.0198
Cela1	chymotrypsin-like elastase family, member 1	-1.33	0.0435
Olr423	olfactory receptor 423	-1.33	0.0462
Apcdd1l	adenomatosis polyposis coli down-regulated 1-like	-1.33	0.0150
Hirip3	HIRA interacting protein 3	-1.33	0.0122
Prkce	protein kinase C, epsilon	-1.33	0.0208
Chac2	ChaC, cation transport regulator homolog 2 (E. coli)	-1.33	0.0280
Sec61a1	Sec61 translocon alpha 1 subunit	-1.33	0.0076
Dleu2	deleted in lymphocytic leukemia 2	-1.33	0.0447
RGD1305733	similar to RIKEN cDNA 2900011O08	-1.33	0.0101
Dock9	dedicator of cytokinesis 9	-1.33	0.0120
Gmfb	glia maturation factor, beta	-1.33	0.0400
Dnajc3	DnaJ (Hsp40) homolog, subfamily C, member 3	-1.33	0.0262
Atad2b	ATPase family, AAA domain containing 2B	-1.33	0.0205
Lrch1	leucine-rich repeats and calponin homology (CH) domain containing 1	-1.33	0.0186
Vps9d1	VPS9 domain containing 1	-1.33	0.0415
Prss29	protease, serine, 29 [Source:RGD Symbol;Acc:1305856]	-1.33	0.0479
Zfhx4	zinc finger homeobox 4	-1.33	0.0319
Porcn	porcupine homolog (Drosophila)	-1.33	0.0108

Myo9b	myosin IXb	-1.34	0.0479
Ddx56	DEAD (Asp-Glu-Ala-Asp) box helicase 56	-1.34	0.0323
Sox6	SRY box 6	-1.34	0.0292
Apcs	amyloid P component, serum	-1.34	0.0437
Igsf5	immunoglobulin superfamily, member 5	-1.34	0.0091
Znhit6	zinc finger, HIT-type containing 6	-1.34	0.0051
Mphosph8	M-phase phosphoprotein 8	-1.34	0.0344
Col18a1	collagen, type XVIII, alpha 1	-1.34	0.0059
Rdh7	retinol dehydrogenase 7	-1.34	0.0058
Ptprg	protein tyrosine phosphatase, receptor type, G	-1.34	0.0256
Snx10	sorting nexin 10	-1.34	0.0034
Olr1621	olfactory receptor 1621	-1.34	0.0248
Gmppa	GDP-mannose pyrophosphorylase A	-1.34	0.0401
Pls3	plastin 3	-1.34	0.0253
Camkk2	calcium/calmodulin-dependent protein kinase kinase 2, beta	-1.34	0.0332
Scn1b	sodium channel, voltage-gated, type I, beta subunit	-1.34	0.0408
RGD1307443	similar to mKIAA0319 protein	-1.34	0.0429
Slc44a5	solute carrier family 44, member 5	-1.34	0.0145
LOC100909934	60S acidic ribosomal protein P0-like	-1.34	0.0109
Sirt1	Protein Sirt1	-1.34	0.0026
LOC102555551	uncharacterized LOC102555551	-1.34	0.0206
Scn9a	sodium channel, voltage-gated, type IX, alpha subunit	-1.34	0.0175
Upb1	ureidopropionase, beta	-1.34	0.0044
Syvn1	synovial apoptosis inhibitor 1, synoviolin	-1.34	0.0113
Fam19a4	family with sequence similarity 19 (chemokine (C-C motif)-like), member A4	-1.34	0.0392
Tfap2c	transcription factor AP-2 gamma	-1.34	0.0377
Olr1247	ENCODES a protein that exhibits olfactory receptor activity (inferred)	-1.34	0.0150
Olr1247	olfactory receptor 1247	-1.34	0.0150
Sox13	SRY box 13	-1.34	0.0349
Phkg2	phosphorylase kinase, gamma 2 (testis)	-1.34	0.0024
Epdr1	ependymin related 1	-1.34	0.0134
Topors	topoisomerase I binding, arginine/serine-rich, E3 ubiquitin protein ligase	-1.34	0.0020
Pfkp	phosphofructokinase, platelet	-1.35	0.0240
Ccdc185	coiled-coil domain containing 185	-1.35	0.0283
Vom1r55	vomeronasal 1 receptor 55	-1.35	0.0438
Tspan12	tetraspanin 12	-1.35	0.0224
Spcs2	signal peptidase complex subunit 2	-1.35	0.0195
Olr312	olfactory receptor 312	-1.35	0.0224
LOC688657	similar to Olfactory receptor 5D13	-1.35	0.0194
Krtap31-1	keratin associated protein 31-1	-1.35	0.0405
Krt81	keratin 81, type II	-1.35	0.0290
Smpdl3a	sphingomyelin phosphodiesterase, acid-like 3A	-1.35	0.0301
Dlk1	delta-like 1 homolog (Drosophila)	-1.35	0.0134
Rplp2	ribosomal protein, large P2	-1.35	0.0070
Sdc2	syndecan 2	-1.35	0.0001
Polr1a	polymerase (RNA) I polypeptide A	-1.35	0.0204
Spen	spen family transcriptional repressor	-1.35	0.0334
LOC689412	similar to CG4025-PA	-1.35	0.0450
LOC100910021	phosphatidylinositol 45-bisphosphate 3-kinase catalytic subunit beta isoform-like	-1.35	0.0052
Kif24	kinesin family member 24	-1.35	0.0008
Cops8	COP9 signalosome subunit 8	-1.35	0.0017
LOC102553962	KRAB domain-containing protein ZNF747-like	-1.35	0.0181
LOC100911032	uncharacterized LOC100911032	-1.35	0.0375
Fam183b	family with sequence similarity 183, member B	-1.35	0.0323
Kpna7	karyopherin alpha 7	-1.35	0.0060
Cd83	CD83 molecule	-1.35	0.0357
Epc2	enhancer of polycomb homolog 2 (Drosophila)	-1.35	0.0010

Vegfa	vascular endothelial growth factor A	-1.35	0.0056
Cep120	centrosomal protein 120	-1.36	0.0165
Ttyh3	tweety family member 3	-1.36	0.0251
Nol8	nucleolar protein 8	-1.36	0.0005
Jmjd1c	jumonji domain containing 1C	-1.36	0.0117
Tango6	transport and golgi organization 6 homolog	-1.36	0.0079
Slc33a1	solute carrier family 33 (acetyl-CoA transporter), member 1	-1.36	0.0137
LOC102552483	uncharacterized LOC102552483	-1.36	0.0034
Hinfp	histone H4 transcription factor	-1.36	0.0202
Acadm	acyl-CoA dehydrogenase, C-4 to C-12 straight chain	-1.36	0.0180
LOC100912366	uncharacterized LOC100912366	-1.36	0.0443
Bbs9	INVOLVED IN cilium assembly (ortholog)	-1.36	0.0326
Slco1a1	solute carrier organic anion transporter family, member 1a1	-1.36	0.0074
Stim2	stromal interaction molecule 2	-1.36	0.0133
Mfsd8	major facilitator superfamily domain containing 8	-1.36	0.0246
LOC300308	similar to hypothetical protein 4930509O22	-1.36	0.0017
LOC100909486	putative SMEK homolog 3-like	-1.36	0.0019
Fmo6	flavin containing monooxygenase 6	-1.36	0.0123
LOC103694381	lymphotoxin-beta	-1.36	0.0250
RGD1559909	RGD1559909	-1.36	0.0253
AABR07054451.1	---	-1.36	0.0489
Ube2b	ubiquitin-conjugating enzyme E2B	-1.36	0.0082
Pcbp4	poly(rC) binding protein 4	-1.36	0.0472
Pdia6	protein disulfide isomerase family A, member 6	-1.36	0.0046
Pik3c3	phosphatidylinositol 3-kinase, catalytic subunit type 3	-1.36	0.0209
Dnaaf2	dynein, axonemal, assembly factor 2	-1.36	0.0447
Arhgap44	Rho GTPase activating protein 44	-1.36	0.0221
Zfp131	zinc finger protein 131	-1.36	0.0099
LOC680394	INTERACTS WITH 2 3 7 8-tetrachlorodibenzodioxine (ortholog)	-1.36	0.0364
Ccdc162	coiled-coil domain containing 162	-1.37	0.0315
Dcun1d1	DCN1, defective in cullin neddylation 1, domain containing 1 (S. cerevisiae) (Dcun1d1), mRNA.	-1.37	0.0156
Ivns1abp	influenza virus NS1A binding protein	-1.37	0.0117
Fam179b	family with sequence similarity 179, member B	-1.37	0.0272
Tnrc6c	trinucleotide repeat containing 6C	-1.37	0.0140
Olr1583	olfactory receptor 1583	-1.37	0.0124
Il6st	interleukin 6 signal transducer	-1.37	0.0033
Nlrc4	NLR family, CARD domain containing 4	-1.37	0.0047
Pik3cb	phosphatidylinositol-4,5-bisphosphate 3-kinase, catalytic subunit beta	-1.37	0.0007
Slc7a4	solute carrier family 7, member 4	-1.37	0.0085
Brd2	bromodomain containing 2	-1.37	0.0108
Nfe2l2	nuclear factor, erythroid 2-like 2	-1.37	0.0198
Nt5c3b	5-nucleotidase, cytosolic IIIB	-1.37	0.0227
Syce3	synaptonemal complex central element protein 3	-1.37	0.0080
LOC102547287	zinc finger protein 728-like	-1.37	0.0344
Tmem164	transmembrane protein 164	-1.37	0.0429
Six5	Protein Six5	-1.37	0.0083
RGD1564149	similar to Protein C21orf58	-1.37	0.0036
Eml4	echinoderm microtubule associated protein like 4	-1.37	0.0372
LOC100363537	ribosomal protein L10a-like [Source:RGD Symbol;Acc:2322811]	-1.37	0.0462
---	---	-1.37	0.0167
Olr505	olfactory receptor 505	-1.37	0.0110
LOC100363408	INTERACTS WITH all-trans-retinoic acid (ortholog) AND amiodarone (ortholog) AND hydrogen peroxide (ortholog)	-1.37	0.0244
Nlrp12	NLR family, pyrin domain containing 12	-1.37	0.0319
Kdm6a	lysine (K)-specific demethylase 6A	-1.37	0.0174
Rrnad1	ribosomal RNA adenine dimethylase domain containing 1	-1.37	0.0297

Slc7a1	solute carrier family 7 (cationic amino acid transporter, y+ system), member 1	-1.37	0.0293
Tank	TRAF family member-associated NFKB activator	-1.37	0.0168
Gja6	gap junction protein, alpha 6	-1.37	0.0378
Olr25	olfactory receptor 25	-1.38	0.0058
Nek3	NIMA-related kinase 3 [Source:RGD Symbol;Acc:1307419]	-1.38	0.0148
RGD1564614	similar to complement factor H-related protein	-1.38	0.0006
Srsf7	serine/arginine-rich splicing factor 7	-1.38	0.0115
Jmy	junction-mediating and regulatory protein	-1.38	0.0062
Dpyd	dihydropyrimidine dehydrogenase	-1.38	0.0084
Rlf	rearranged L-myc fusion	-1.38	0.0001
Gne	glucosamine (UDP-N-acetyl)-2-epimerase/N-acetylmannosamine kinase	-1.38	0.0430
Bbs2	Bardet-Biedl syndrome 2	-1.38	0.0295
RGD1565002	similar to Dehydrogenase/reductase SDR family member 7 precursor (Retinal short-chain dehydrogenase/reductase 4)	-1.38	0.0150
LOC103694398	keratin-associated protein 10-1-like	-1.38	0.0478
RGD1310257	similar to RIKEN cDNA 6330408A02 gene	-1.38	0.0136
Elavl3	ELAV like neuron-specific RNA binding protein 3	-1.38	0.0226
Olr1376	olfactory receptor 1376	-1.38	0.0435
Flcn	folliculin	-1.38	0.0239
Lpo	lactoperoxidase	-1.38	0.0093
Ldhd	lactate dehydrogenase D	-1.38	0.0347
Klk1c8	kallikrein 1-related peptidase C8	-1.38	0.0055
Trappc6b	trafficking protein particle complex 6B	-1.38	0.0079
Oser1	oxidative stress responsive serine-rich 1	-1.38	0.0267
Crygd	crystallin, gamma D	-1.38	0.0387
Zc2hc1c	zinc finger, C2HC-type containing 1C	-1.38	0.0474
Os9	osteosarcoma amplified 9, endoplasmic reticulum lectin	-1.38	0.0013
Slc38a3	solute carrier family 38, member 3	-1.38	0.0030
06. Mrz	membrane-associated ring finger (C3HC4) 6, E3 ubiquitin protein ligase	-1.38	0.0151
Erbb3	erb-b2 receptor tyrosine kinase 3	-1.38	0.0316
Arsb	arylsulfatase B	-1.39	0.0170
Ccnl1	cyclin L1	-1.39	0.0229
Sidt1	SID1 transmembrane family, member 1	-1.39	0.0325
Snapc1	small nuclear RNA activating complex, polypeptide 1	-1.39	0.0054
Rlim	ring finger protein, LIM domain interacting	-1.39	0.0127
Antxr2	anthrax toxin receptor 2	-1.39	0.0109
Ctdsp2	CTD (carboxy-terminal domain, RNA polymerase II, polypeptide A) small phosphatase 2 [Source:RGD Symbol;Acc:2319209]	-1.39	0.0489
Tspyl2	TSPY-like 2	-1.39	0.0358
Ly6h	lymphocyte antigen 6 complex, locus H	-1.39	0.0019
Iqcb1	IQ motif containing B1	-1.39	0.0050
Sphk1	sphingosine kinase 1	-1.39	0.0050
Whsc1l1	Wolf-Hirschhorn syndrome candidate 1-like 1	-1.39	0.0081
Itpr2	inositol 1,4,5-trisphosphate receptor, type 2	-1.39	0.0119
Snx20	sorting nexin 20	-1.39	0.0058
Pros1	protein S (alpha)	-1.39	0.0004
Tc2n	tandem C2 domains, nuclear	-1.39	0.0198
Tmem63b	transmembrane protein 63B	-1.39	0.0120
RGD1562265	similar to ribosomal protein S12 [Source:RGD Symbol;Acc:1562265]	-1.39	0.0004
Blcap	bladder cancer associated protein	-1.39	0.0250
Nhlrc2	NHL repeat containing 2	-1.39	0.0490
Dll1	delta-like 1 (Drosophila)	-1.39	0.0333
Dopey2	dopey family member 2	-1.39	0.0035
LOC686677	similar to olfactory receptor 1161	-1.39	0.0239
Ugt2b35	UDP glucuronosyltransferase 2 family, polypeptide B35	-1.39	0.0019
RT1-T18	RT1 class Ib, locus T18	-1.39	0.0031
Krtap9-1	keratin associated protein 9-1 [Source:RGD Symbol;Acc:1593000]	-1.39	0.0025

Zfp496	zinc finger protein 496	-1.40	0.0088
Sv2b	synaptic vesicle glycoprotein 2b	-1.40	0.0235
Pink1	PTEN induced putative kinase 1	-1.40	0.0119
Nfat5	ENCODES a protein that exhibits DNA binding (ortholog)	-1.40	0.0064
Ccnt1	cyclin T1	-1.40	0.0120
Olr1060	olfactory receptor 1060	-1.40	0.0354
Mef2a	myocyte enhancer factor 2a	-1.40	0.0046
Cps1	carbamoyl-phosphate synthetase 1	-1.40	0.0034
Brd2	bromodomain containing 2 [Source:RGD Symbol;Acc:1303324]	-1.40	0.0364
Gapvd1	GTPase activating protein and VPS9 domains 1	-1.40	0.0019
Tnks2	tankyrase, TRF1-interacting ankyrin-related ADP-ribose polymerase 2	-1.40	0.0013
Wdsub1	WD repeat, sterile alpha motif and U-box domain containing 1	-1.40	0.0142
RGD1560112	similar to Urinary protein 2 precursor (RUP-2)	-1.40	0.0131
St8sia3	ST8 alpha-N-acetyl-neuraminide alpha-2,8-sialyltransferase 3	-1.40	0.0003
Txndc5	thioredoxin domain containing 5 (endoplasmic reticulum)	-1.40	0.0108
Olr1668	olfactory receptor 1668	-1.40	0.0172
Cpeb4	cytoplasmic polyadenylation element binding protein 4	-1.40	0.0384
Gpr55	G protein-coupled receptor 55	-1.40	0.0157
Sec14l2	SEC14-like lipid binding 2	-1.40	0.0274
LOC102553248	uncharacterized LOC102553248	-1.40	0.0027
Sbp	spermine binding protein	-1.40	0.0406
Hip1r	huntingtin interacting protein 1 related	-1.40	0.0360
Dnajc25	DnaJ (Hsp40) homolog, subfamily C, member 25	-1.40	0.0048
RT1-A	RT1 class I, locus A	-1.40	0.0131
Ang	angiogenin, ribonuclease, RNase A family, 5	-1.40	0.0004
Baat	bile acid CoA:amino acid N-acyltransferase	-1.40	0.0003
Olr336	olfactory receptor 336	-1.41	0.0307
Rnf169	ring finger protein 169	-1.41	0.0469
Srrd	SRR1 domain containing	-1.41	0.0081
Ang2	angiogenin, ribonuclease A family, member 2	-1.41	0.0067
Lrrc69	leucine rich repeat containing 69	-1.41	0.0466
LOC500077	similar to RIKEN cDNA 3110062M04	-1.41	0.0200
Rel	Protein Rel	-1.41	0.0049
Slc2a8	solute carrier family 2, (facilitated glucose transporter) member 8	-1.41	0.0085
LOC100359876	ENCODES a protein that exhibits ATP binding (inferred)	-1.41	0.0468
Tmem43	transmembrane protein 43	-1.41	0.0468
Cd8a	CD8a molecule	-1.41	0.0112
Epb41l1	erythrocyte membrane protein band 4.1-like 1	-1.41	0.0127
Abo	ABO blood group (transferase A, alpha 1-3-N-acetylgalactosaminyltransferase; transferase B, alpha 1-3-galactosyltransferase)	-1.41	0.0354
Acox3	acyl-CoA oxidase 3, pristanoyl	-1.41	0.0056
Cyp2g1	cytochrome P450, family 2, subfamily g, polypeptide 1	-1.41	0.0047
Sorbs2	sorbin and SH3 domain containing 2	-1.41	0.0455
LOC102550789	RING finger protein C14orf164 homolog	-1.41	0.0322
LOC100360157	1110034B05Rik protein-like	-1.41	0.0419
Impdh2	IMP (inosine 5-monophosphate) dehydrogenase 2	-1.41	0.0164
Bin2	bridging integrator 2	-1.41	0.0126
Arhgap42	Rho GTPase activating protein 42	-1.41	0.0236
Isl2	ISL LIM homeobox 2	-1.41	0.0018
Capns1	ENCODES a protein that exhibits calcium-dependent cysteine-type endopeptidase activity	-1.41	0.0408
Tgm1	transglutaminase 1	-1.41	0.0043
Adamts7	ADAM metallopeptidase with thrombospondin type 1 motif, 7	-1.41	0.0109
Ogfrl1	opioid growth factor receptor-like 1	-1.41	0.0032
Clip4	CAP-GLY domain containing linker protein family, member 4	-1.41	0.0376
Vipr1	vasoactive intestinal peptide receptor 1	-1.41	0.0075
Samt4	spermatogenesis associated multipass transmembrane protein 4	-1.41	0.0460

Abca6	ATP-binding cassette, subfamily A (ABC1), member 6	-1.41	0.0112
Kat2b	K(lysine) acetyltransferase 2B	-1.41	0.0235
LOC100912024	uncharacterized LOC100912024 [Source:RGD Symbol;Acc:6489434]	-1.42	0.0347
LOC102546342	uncharacterized LOC102546342	-1.42	0.0397
Paics	phosphoribosylaminoimidazole carboxylase, phosphoribosylaminoimidazole succinocarboxamide synthetase	-1.42	0.0085
RGD1565806	similar to 60S ribosomal protein L23a	-1.42	0.0425
Eqtn	equatorin, sperm acrosome associated	-1.42	0.0068
Olr1737	olfactory receptor 1737	-1.42	0.0235
Dock4	dedicator of cytokinesis 4 [Source:RGD Symbol;Acc:1561724]	-1.42	0.0498
Slc2a12	solute carrier family 2 (facilitated glucose transporter), member 12	-1.42	0.0403
---	Uncharacterized protein	-1.42	0.0175
Srf	serum response factor	-1.42	0.0407
Naa25	N(alpha)-acetyltransferase 25, NatB auxiliary subunit	-1.42	0.0050
LOC686084	similar to CG32580-PA	-1.42	0.0135
LOC680700	similar to ribosomal protein L10a [Source:RGD Symbol;Acc:1588266]	-1.42	0.0346
Fndc3a	fibronectin type III domain containing 3a	-1.42	0.0007
Nr4a2	nuclear receptor subfamily 4, group A, member 2	-1.42	0.0148
Rbm15	RNA binding motif protein 15	-1.42	0.0235
Cdkl5	cyclin-dependent kinase-like 5	-1.42	0.0376
NONMMUG03 2780	Non-coding transcript identified by NONCODE: Linc	-1.42	0.0301
Tirap	toll-interleukin 1 receptor (TIR) domain-containing adaptor protein	-1.42	0.0353
Atp2c1	ATPase, Ca++ transporting, type 2C, member 1	-1.43	0.0159
Tas2r129	taste receptor, type 2, member 129	-1.43	0.0039
Dip2c	disco-interacting protein 2 homolog C	-1.43	0.0153
Klrc1	killer cell lectin-like receptor subfamily C, member 1	-1.43	0.0492
Dbndd2	dysbindin (dystrobrevin binding protein 1) domain containing 2	-1.43	0.0267
Gltscr1l	GLTSCR1-like	-1.43	0.0123
Agbl1	ATP/GTP binding protein-like 1 [Source:RGD Symbol;Acc:1560280]	-1.43	0.0043
Pogk	pogo transposable element with KRAB domain	-1.43	0.0096
LOC689574	hypothetical protein LOC689574	-1.43	0.0121
Ssr4	signal sequence receptor, delta	-1.43	0.0022
Chp1	calcineurin-like EF-hand protein 1	-1.43	0.0083
Pbld2	INTERACTS WITH 17beta-estradiol (ortholog) AND 2 3 7 8-tetrachlorodibenzodioxine (ortholog) AND aflatoxin B1 (ortholog)	-1.43	0.0254
Arhgap6	Rho GTPase activating protein 6	-1.43	0.0091
---	Uncharacterized protein	-1.43	0.0224
Arl4d	ADP-ribosylation factor-like 4D	-1.43	0.0053
Apol11a	apolipoprotein L 11a	-1.43	0.0241
Klrc3	killer cell lectin-like receptor subfamily C, member 3	-1.43	0.0489
Gramd1c	GRAM domain containing 1C	-1.43	0.0245
Dtx1	deltex 1, E3 ubiquitin ligase	-1.43	0.0127
Zcchc2	zinc finger, CCHC domain containing 2	-1.43	0.0444
Nr1h4	nuclear receptor subfamily 1, group H, member 4	-1.43	0.0158
Trip13	thyroid hormone receptor interactor 13	-1.44	0.0243
LOC100910308	INTERACTS WITH 1-chloro-2 4-dinitrobenzene (ortholog) AND 17beta-estradiol (ortholog) AND 2 3 7 8-tetrachlorodibenzodioxine (ortholog)	-1.44	0.0498
Olr1	oxidized low density lipoprotein (lectin-like) receptor 1	-1.44	0.0297
Alox5ap	arachidonate 5-lipoxygenase activating protein	-1.44	0.0108
Ppp2r3a	protein phosphatase 2, regulatory subunit B, alpha	-1.44	0.0079
Olr281	olfactory receptor 281 (Olr281), mRNA	-1.44	0.0069
Gstm6	glutathione S-transferase, mu 6	-1.44	0.0050
Rnf40	ring finger protein 40, E3 ubiquitin protein ligase	-1.44	0.0145
RGD1559979	similar to APH1B homolog (C.elegans)	-1.44	0.0303
Rnf148	ring finger protein 148	-1.44	0.0095
Gmeb1	glucocorticoid modulatory element binding protein 1 (Gmeb1), mRNA. Chalmel, et. al. Testis-expressed Unannotated Transcripts (TUTs)	-1.44	0.0404

Olr964	olfactory receptor 964	-1.44	0.0008
Ppm1l	protein phosphatase, Mg2+/Mn2+ dependent, 1L	-1.44	0.0167
Mafg	v-maf avian musculoaponeurotic fibrosarcoma oncogene homolog G	-1.44	0.0117
Gk	glycerol kinase	-1.44	0.0230
Arl5b	ADP-ribosylation factor-like 5B	-1.44	0.0140
LOC100361579	prolactin family 5 subfamily a member 2-like	-1.44	0.0117
Paxbp1	PAX3 and PAX7 binding protein 1	-1.44	0.0093
Sycp1	synaptonemal complex protein 1	-1.44	0.0255
Tmem246	transmembrane protein 246	-1.44	0.0060
Hs3st3b1	heparan sulfate (glucosamine) 3-O-sulfotransferase 3B1	-1.44	0.0381
Fzd3	frizzled class receptor 3	-1.45	0.0284
Ang2	angiogenin, ribonuclease A family, member 2	-1.45	0.0001
Nkg7	natural killer cell granule protein 7	-1.45	0.0200
Cept1	choline/ethanolamine phosphotransferase 1	-1.45	0.0006
Qtrtd1	Protein Qtrtd1	-1.45	0.0451
Wasf1	WAS protein family, member 1	-1.45	0.0054
Slc35e2b	solute carrier family 35, member E2B	-1.45	0.0166
Dock7	dedicator of cytokinesis 7	-1.45	0.0192
Gclm	glutamate cysteine ligase, modifier subunit	-1.45	0.0172
Lpar1	lysophosphatidic acid receptor 1	-1.45	0.0291
Ar	androgen receptor	-1.45	0.0317
LOC689499	similar to Y97E10AL.1	-1.45	0.0387
Dhrs3	dehydrogenase/reductase (SDR family) member 3	-1.45	0.0436
Rhbdl2	rhomboid, veinlet-like 2 (Drosophila)	-1.45	0.0008
Phf19	PHD finger protein 19	-1.46	0.0403
Pygo2	pygopus family PHD finger 2	-1.46	0.0302
Sgpl1	sphingosine-1-phosphate lyase 1	-1.46	0.0196
Ly49i5	Ly49 inhibitory receptor 5	-1.46	0.0231
Cep112	centrosomal protein 112kDa	-1.46	0.0041
Ctsc	cathepsin C	-1.46	0.0002
Triap1	TP53 regulated inhibitor of apoptosis 1	-1.46	0.0378
Ppp1r2	protein phosphatase 1, regulatory (inhibitor) subunit 2	-1.46	0.0019
Olr1682	olfactory receptor 1682	-1.46	0.0350
Neo1	Neogenin	-1.46	0.0126
Ddx39a	DEAD (Asp-Glu-Ala-Asp) box polypeptide 39A	-1.46	0.0259
Smc6	structural maintenance of chromosomes 6	-1.46	0.0049
Phyh	phytanoyl-CoA 2-hydroxylase	-1.46	0.0147
Rpl18a	ribosomal protein L18A	-1.46	0.0078
LOC102554977	INTERACTS WITH cisplatin (ortholog) AND progesterone (ortholog)	-1.46	0.0293
Olr329	olfactory receptor 329	-1.46	0.0015
Ssfa2	sperm specific antigen 2	-1.46	0.0356
Glod5	glyoxalase domain containing 5	-1.47	0.0442
Cyp4f6	cytochrome P450, family 4, subfamily f, polypeptide 6	-1.47	0.0002
Cd302	CD302 molecule	-1.47	0.0000
Cflar	CASP8 and FADD-like apoptosis regulator	-1.47	0.0086
Zfp84	zinc finger protein 84	-1.47	0.0034
Slc50a1	solute carrier family 50 (sugar efflux transporter), member 1	-1.47	0.0086
Mt4	metallothionein 4	-1.47	0.0278
Actn2	actinin alpha 2	-1.47	0.0202
Kmo	kynurenine 3-monooxygenase (kynurenine 3-hydroxylase)	-1.47	0.0021
Ripk1	receptor (TNFRSF)-interacting serine-threonine kinase 1	-1.47	0.0022
Usp1	ubiquitin specific peptidase 1	-1.47	0.0025
Ppp1r15a	protein phosphatase 1, regulatory subunit 15A	-1.47	0.0099
Sh2d1a	SH2 domain containing 1A	-1.47	0.0039
Unc79	INVOLVED IN adult behavior (ortholog)	-1.47	0.0246
Fcna	ficolin A	-1.47	0.0352
Zfp1	zinc finger protein 1	-1.47	0.0221
RGD1311447	LOC363276	-1.47	0.0262

RGD1565088	similar to UPF0197 protein C11orf10 homolog	-1.47	0.0221
Rngtt	RNA guanylyltransferase and 5-phosphatase	-1.47	0.0035
RT1-CE10	RT1 class I, locus CE10	-1.47	0.0249
Lpar2	lysophosphatidic acid receptor 2	-1.47	0.0112
LOC102556148	ENCODES a protein that exhibits N-acetyltransferase activity (inferred) AND INVOLVED IN metabolic process (inferred)	-1.48	0.0244
Cd300lf	Cd300 molecule-like family member F	-1.48	0.0078
Rpl22l2	ribosomal protein L22-like 2 [Source:RGD Symbol;Acc:1590585]	-1.48	0.0151
Atg13	autophagy related 13	-1.48	0.0059
Eps8l2	EPS8-like 2	-1.48	0.0413
N4bp2	NEDD4 binding protein 2	-1.48	0.0128
Ehd3	EH-domain containing 3	-1.48	0.0222
Ctsl	cathepsin L	-1.48	0.0249
Il1rapl1	interleukin 1 receptor accessory protein-like 1	-1.48	0.0126
Olr1399	olfactory receptor 1399	-1.48	0.0158
Txndc11	thioredoxin domain containing 11	-1.48	0.0003
Me3	malic enzyme 3, NADP(+)-dependent, mitochondrial	-1.48	0.0038
Poglut1	protein O-glucosyltransferase 1	-1.49	0.0008
Susd1	INTERACTS WITH indole-3-methanol AND thioacetamide AND 2 3 7 8-tetrachlorodibenzodioxine (ortholog)	-1.49	0.0421
RGD1560556	similar to C14orf25 protein	-1.49	0.0257
Nuggc	nuclear GTPase, germinal center associated	-1.49	0.0441
Zfp874b	zinc finger protein 874b	-1.49	0.0334
Abcb4	ATP-binding cassette, subfamily B (MDR/TAP), member 4	-1.49	0.0376
C1rl	complement component 1, r subcomponent-like	-1.49	0.0007
Dpy19l3	dpy-19-like 3 (C. elegans)	-1.49	0.0284
Taf1c	TATA box binding protein (Tbp)-associated factor, RNA polymerase I, C	-1.49	0.0061
Ints6	integrator complex subunit 6	-1.49	0.0109
Ipo4	importin 4	-1.49	0.0031
Slc25a13	solute carrier family 25 (aspartate/glutamate carrier), member 13 [Source:RGD Symbol;Acc:1565889]	-1.49	0.0001
C6	complement component 6	-1.50	0.0174
Dbr1	debranching RNA lariats 1	-1.50	0.0033
Ms4a5	membrane-spanning 4-domains, subfamily A, member 5	-1.50	0.0319
Lats2	large tumor suppressor kinase 2	-1.50	0.0001
LOC100363171	histone variant H2al2-like	-1.50	0.0027
Eif2ak2	eukaryotic translation initiation factor 2-alpha kinase 2	-1.50	0.0260
Xpnpep3	X-prolyl aminopeptidase 3, mitochondrial	-1.50	0.0242
Tcf7l2	transcription factor 7-like 2 (T-cell specific, HMG-box)	-1.50	0.0354
Olr1242	olfactory receptor 1242	-1.51	0.0150
Klf12	Kruppel-like factor 12	-1.51	0.0247
Olr483	olfactory receptor 483	-1.51	0.0051
Thumpd1	THUMP domain containing 1	-1.51	0.0201
Fam3a	family with sequence similarity 3, member A	-1.51	0.0011
Tmem47	transmembrane protein 47	-1.51	0.0023
Oas1i	2 -5 oligoadenylate synthetase 1I	-1.51	0.0435
Mllt3	myeloid/lymphoid or mixed-lineage leukemia; translocated to, 3	-1.51	0.0424
Birc2	baculoviral IAP repeat-containing 2	-1.51	0.0115
Ppp2r5e	protein phosphatase 2, regulatory subunit B, epsilon isoform	-1.51	0.0007
Adgrl2	adhesion G protein-coupled receptor L2	-1.51	0.0051
Nt5e	5 nucleotidase, ecto	-1.51	0.0497
Fads6	fatty acid desaturase 6	-1.51	0.0124
Dram2	DNA-damage regulated autophagy modulator 2	-1.51	0.0036
Prelid2	PRELI domain containing 2	-1.51	0.0448
Map3k14	mitogen-activated protein kinase kinase kinase 14	-1.51	0.0037
Ahctf1	AT hook containing transcription factor 1	-1.51	0.0153
Cyp4f39	cytochrome P450, family 4, subfamily f, polypeptide 39	-1.52	0.0278
Olr281	olfactory receptor 281	-1.52	0.0052

Ddost	dolichyl-diphosphooligosaccharide--protein glycosyltransferase subunit (non-catalytic)	-1.52	0.0006
LOC499136	LRRGT00021	-1.52	0.0022
Fshr	follicle stimulating hormone receptor	-1.52	0.0076
Map4k5	mitogen-activated protein kinase kinase kinase kinase 5	-1.52	0.0031
Alkbh1	alkB homolog 1, histone H2A dioxygenase	-1.52	0.0112
Usp12	ubiquitin specific peptidase 12	-1.52	0.0009
Arntl2	aryl hydrocarbon receptor nuclear translocator-like 2	-1.52	0.0368
Nup88	nucleoporin 88	-1.52	0.0027
RGD1565410	similar to Ly6-C antigen gene	-1.52	0.0020
Whamm	WAS protein homolog associated with actin, golgi membranes and microtubules	-1.52	0.0021
Gstm6l	glutathione S-transferase, mu 6-like	-1.52	0.0040
Ehbp1	EH domain binding protein 1	-1.52	0.0133
Ackr2	atypical chemokine receptor 2	-1.53	0.0203
Nxf2	nuclear RNA export factor 2	-1.53	0.0007
St3gal1	ST3 beta-galactoside alpha-2,3-sialyltransferase 1	-1.53	0.0353
Krt82	keratin 82, type II	-1.53	0.0073
Mapk14	mitogen activated protein kinase 14	-1.53	0.0007
LOC100909605	serine protease inhibitor A3F-like [Source:RGD Symbol;Acc:6502633]	-1.53	0.0013
Olr718	olfactory receptor 718	-1.53	0.0310
Irf9	interferon regulatory factor 9	-1.53	0.0094
LOC100909605	serine protease inhibitor A3F-like [Source:RGD Symbol;Acc:6502633]	-1.53	0.0052
Trak1	trafficking protein, kinesin binding 1	-1.53	0.0156
Gstm3	glutathione S-transferase mu 3	-1.53	0.0169
Klkb1	kallikrein B, plasma 1	-1.53	0.0004
Fgfrl1	fibroblast growth factor receptor-like 1	-1.53	0.0470
Nmnat1	nicotinamide nucleotide adenylyltransferase 1	-1.53	0.0137
Osbpl8	oxysterol binding protein-like 8	-1.53	0.0009
Aim2	absent in melanoma 2	-1.53	0.0151
Ccdc117	coiled-coil domain containing 117	-1.54	0.0190
Lrg1	leucine-rich alpha-2-glycoprotein 1	-1.54	0.0248
Dmxl2	Dmx-like 2	-1.54	0.0231
Zbtb43	zinc finger and BTB domain containing 43	-1.54	0.0060
Crtc3	CREB regulated transcription coactivator 3	-1.54	0.0053
Cyyr1	cysteine/tyrosine-rich 1	-1.54	0.0480
LOC680200	similar to zinc finger protein 455	-1.54	0.0135
Hlcs	holocarboxylase synthetase (biotin-(proprionyl-CoA-carboxylase (ATP-hydrolysing)) ligase)	-1.54	0.0157
Tmx4	thioredoxin-related transmembrane protein 4	-1.54	0.0176
Mov10l1	Mov10 RISC complex RNA helicase like 1	-1.54	0.0013
Ctdspl	CTD (carboxy-terminal domain, RNA polymerase II, polypeptide A) small phosphatase-like	-1.55	0.0316
Tymp	thymidine phosphorylase	-1.55	0.0026
Mad2l1bp	MAD2L1 binding protein	-1.55	0.0064
Slc10a2	solute carrier family 10 (sodium/bile acid cotransporter), member 2	-1.55	0.0432
Nrxn1	neurexin 1	-1.55	0.0471
RGD1560203	Protein RGD1560203	-1.55	0.0304
Mid1	midline 1	-1.55	0.0019
Dlc1	DLC1 Rho GTPase activating protein	-1.55	0.0012
Hsd3b5	hydroxy-delta-5-steroid dehydrogenase, 3 beta- and steroid delta-isomerase 5	-1.55	0.0127
Prg4	proteoglycan 4	-1.55	0.0053
Cks1b	CDC28 protein kinase regulatory subunit 1B	-1.55	0.0471
Pkmyt1	protein kinase, membrane associated tyrosine/threonine 1	-1.55	0.0032
Yipf2	Yip1 domain family, member 2	-1.55	0.0077
Rbms1	RNA binding motif, single stranded interacting protein 1	-1.55	0.0024
Cyb5a	cytochrome b5 type A (microsomal)	-1.56	0.0169

Hsp90b1	heat shock protein 90, beta, member 1	-1.56	0.0066
Rbpj	recombination signal binding protein for immunoglobulin kappa J region	-1.56	0.0033
Mrpl14	mitochondrial ribosomal protein L14	-1.56	0.0138
Atxn1l	ataxin 1-like	-1.56	0.0069
Mboat1	membrane bound O-acyltransferase domain containing 1	-1.56	0.0227
Olr1450	olfactory receptor 1450	-1.56	0.0136
Ly6c	Ly6-C antigen	-1.56	0.0367
Hsd3b1	hydroxy-delta-5-steroid dehydrogenase, 3 beta- and steroid delta-isomerase 6 (Hsd3b6), mRNA	-1.56	0.0110
Sod3	superoxide dismutase 3, extracellular	-1.57	0.0176
Lyzl1	lysozyme-like 1	-1.57	0.0162
LOC100910689	cytochrome c oxidase assembly protein COX11, mitochondrial-like [Source:RGD Symbol;Acc:6497536]	-1.57	0.0031
RGD1308706	similar to RIKEN cDNA 4921524J17	-1.57	0.0151
Nedd4l	neural precursor cell expressed, developmentally down-regulated 4-like, E3 ubiquitin protein ligase	-1.57	0.0031
Agmat	agmatine ureohydrolase (agmatinase)	-1.57	0.0082
Cysltr1	cysteinyl leukotriene receptor 1	-1.57	0.0116
Pon3	paraoxonase 3	-1.57	0.0030
Stard4	StAR-related lipid transfer (START) domain containing 4	-1.58	0.0237
Il18r1	interleukin 18 receptor 1	-1.58	0.0235
Slc9a7	solute carrier family 9, subfamily A (NHE7, cation proton antiporter 7), member 7	-1.58	0.0115
Gnl3	guanine nucleotide binding protein-like 3 (nucleolar)	-1.58	0.0080
Tmem150c	transmembrane protein 150C	-1.59	0.0344
LOC102550998	sperm motility kinase 3-like	-1.59	0.0133
Rsrp1	arginine/serine-rich protein 1	-1.59	0.0010
Runx1t1	runt-related transcription factor 1; translocated to, 1 (cyclin D-related)	-1.59	0.0277
Trpm6	transient receptor potential cation channel, subfamily M, member 6	-1.59	0.0267
Hsd3b7	hydroxy-delta-5-steroid dehydrogenase, 3 beta- and steroid delta-isomerase 7	-1.59	0.0154
Nkrf	Protein LOC100910054	-1.59	0.0007
Maob	monoamine oxidase B	-1.59	0.0005
LOC102551852	keratin-associated protein 20-2-like	-1.60	0.0044
Nfyb	nuclear transcription factor-Y beta	-1.60	0.0263
Mef2d	myocyte enhancer factor 2D	-1.60	0.0165
Cyp4f1	cytochrome P450, family 4, subfamily f, polypeptide 1	-1.60	0.0001
LOC102548360	MLV-related proviral Env polyprotein-like	-1.60	0.0184
Hao1	hydroxyacid oxidase (glycolate oxidase) 1	-1.60	0.0010
LOC690478	similar to keratin associated protein 10-7	-1.60	0.0225
Ces1e	ENCODES a protein that exhibits palmitoyl-CoA hydrolase activity	-1.60	0.0070
Ier2	immediate early response 2	-1.60	0.0392
Stard13	StAR-related lipid transfer (START) domain containing 13	-1.61	0.0183
Cers3	ceramide synthase 3	-1.61	0.0201
Eif4b	eukaryotic translation initiation factor 4B	-1.61	0.0063
RGD1309821	similar to KIAA1161 protein	-1.61	0.0010
LOC360919	similar to alpha-fetoprotein	-1.61	0.0110
Ddc	dopa decarboxylase (aromatic L-amino acid decarboxylase)	-1.61	0.0171
LOC102551659	MLV-related proviral Env polyprotein-like	-1.61	0.0030
Med13	mediator complex subunit 13	-1.61	0.0114
Ets2	v-ets avian erythroblastosis virus E26 oncogene homolog 2	-1.62	0.0368
Hbegf	heparin-binding EGF-like growth factor	-1.62	0.0155
Prf1	perforin 1 (pore forming protein)	-1.62	0.0012
Mettl7a	methyltransferase like 7A	-1.62	0.0157
LOC100361702	keratin associated protein 12-1-like	-1.62	0.0106
Dusp26	dual specificity phosphatase 26 (putative)	-1.62	0.0228
Enpp1	ectonucleotide pyrophosphatase/phosphodiesterase 1	-1.62	0.0058
Ppp1r3g	protein phosphatase 1, regulatory subunit 3G	-1.62	0.0072

Igf1	insulin-like growth factor 1	-1.63	0.0001
Ccl5	chemokine (C-C motif) ligand 5	-1.63	0.0277
Igfbp4	insulin-like growth factor binding protein 4	-1.63	0.0488
Pcdh1	Protein Pcdh1	-1.64	0.0027
LOC691280	similar to Zinc finger protein 180 (HHZ168)	-1.64	0.0072
Cgrrf1	cell growth regulator with ring finger domain 1	-1.64	0.0007
Aph1b	APH1B gamma secretase subunit	-1.64	0.0023
RGD1565566	similar to 60S ribosomal protein L18a [Source:RGD Symbol;Acc:1565566]	-1.65	0.0217
Mcm7	minichromosome maintenance complex component 7	-1.65	0.0037
Glyatl1	glycine-N-acyltransferase-like 1	-1.65	0.0029
Olr181	olfactory receptor 181	-1.65	0.0077
Lpar3	lysophosphatidic acid receptor 3	-1.65	0.0176
LOC102552619	INTERACTS WITH cobalt dichloride (ortholog) AND crocidolite asbestos (ortholog) AND methyl methanesulfonate (ortholog)	-1.66	0.0026
Pax3	paired box 3	-1.66	0.0026
Hpd	4-hydroxyphenylpyruvate dioxygenase	-1.66	0.0040
Apoa5	apolipoprotein A-V	-1.66	0.0048
Ankrd27	ankyrin repeat domain 27 (VPS9 domain)	-1.67	0.0025
Hoxa2	homeo box A2	-1.67	0.0420
Cyp2j3	cytochrome P450, family 2, subfamily j, polypeptide 3	-1.67	0.0169
Igfals	insulin-like growth factor binding protein, acid labile subunit	-1.68	0.0244
RGD1306091	PREDICTED: similar to Mixed lineage kinase 4 (RGD1306091), mRNA. Chalmel, et. al. Testis-expressed Unannotated Transcripts (TUTs)	-1.68	0.0161
LOC102551832	MLV-related proviral Env polyprotein-like	-1.68	0.0078
Omd	osteomodulin (Omd), mRNA	-1.68	0.0102
Pik3c2g	phosphatidylinositol-4-phosphate 3-kinase, catalytic subunit type 2 gamma	-1.68	0.0072
Tfcp2	transcription factor CP2	-1.68	0.0002
RGD1561986	INTERACTS WITH 17beta-hydroxy-17-methylestra-4 9 11-trien-3-one (ortholog)	-1.69	0.0276
Banp	Btg3 associated nuclear protein	-1.69	0.0026
Slc7a2	solute carrier family 7 (cationic amino acid transporter, y+ system), member 2	-1.69	0.0250
Cyp3a62	cytochrome P450, family 3, subfamily a, polypeptide 62	-1.69	0.0100
Cldn2	claudin 2	-1.69	0.0435
Irs1	insulin receptor substrate 1	-1.69	0.0049
Slc21a4	kidney specific organic anion transporter	-1.69	0.0111
A2m	alpha-2-macroglobulin	-1.70	0.0024
Lect2	leukocyte cell-derived chemotaxin 2	-1.70	0.0017
RGD1564074	similar to novel protein [Source:RGD Symbol;Acc:1564074]	-1.70	0.0295
Hectd2	HECT domain containing E3 ubiquitin protein ligase 2	-1.71	0.0078
Foxp1	forkhead box P1	-1.71	0.0021
Setd1b	SET domain containing 1B [Source:MGI Symbol;Acc:MGI:2652820]	-1.71	0.0073
Lrp6	low density lipoprotein receptor-related protein 6	-1.71	0.0010
Sez6	seizure related 6 homolog (mouse)	-1.71	0.0345
Als2	amyotrophic lateral sclerosis 2 (juvenile)	-1.71	0.0011
Tmem163	transmembrane protein 163	-1.71	0.0134
Zswim1	zinc finger, SWIM-type containing 1	-1.71	0.0005
Ankrd33b	ankyrin repeat domain 33B	-1.71	0.0004
Acss3	acyl-CoA synthetase short-chain family member 3	-1.71	0.0015
Hdac2	histone deacetylase 2	-1.72	0.0060
LOC102549637	filaggrin-2-like	-1.72	0.0213
Omd	osteomodulin	-1.72	0.0057
Rdh16	ENCODES a protein that exhibits retinol dehydrogenase activity	-1.73	0.0013
Kdelc2	KDEL (Lys-Asp-Glu-Leu) containing 2	-1.73	0.0046
Gjb1	gap junction protein, beta 1	-1.73	0.0000
Pla2g12a	phospholipase A2, group XIIA	-1.73	0.0234
LOC102552117	MLV-related proviral Env polyprotein-like	-1.73	0.0179
Mcts2	malignant T cell amplified sequence 2	-1.74	0.0142

Ifitm7	interferon induced transmembrane protein 7 [Source:RGD Symbol;Acc:1306466]	-1.74	0.0010
Clstn1	calsyntenin 1	-1.75	0.0031
Grb7	growth factor receptor bound protein 7	-1.75	0.0330
Xk	X-linked Kx blood group	-1.76	0.0022
Gpd2	glycerol-3-phosphate dehydrogenase 2	-1.76	0.0326
S1pr2	sphingosine-1-phosphate receptor 2	-1.76	0.0001
Rnd2	Rho family GTPase 2	-1.76	0.0057
Dnajc18	DnaJ (Hsp40) homolog, subfamily C, member 18	-1.77	0.0114
Atp11a	ATPase, class VI, type 11A	-1.78	0.0000
Zfp69	zinc finger protein 69 [Source:RGD Symbol;Acc:2322990]	-1.78	0.0131
Ddb2	damage specific DNA binding protein 2	-1.79	0.0020
NEWGENE_1585613	ADP-ribosylation factor-like 14	-1.80	0.0087
Fv1	Friend virus susceptibility 1	-1.80	0.0000
Prrg1	proline rich Gla (G-carboxyglutamic acid) 1	-1.80	0.0196
Gstm2	glutathione S-transferase mu 2	-1.80	0.0057
Ablim3	actin binding LIM protein family, member 3	-1.80	0.0307
Loxl4	lysyl oxidase-like 4	-1.81	0.0017
Cyp3a23/3a1	cytochrome P450, family 3, subfamily a, polypeptide 23/polypeptide 1 (Cyp3a23/3a1), mRNA	-1.81	0.0004
Cyp3a23/3a1	cytochrome P450, family 3, subfamily a, polypeptide 23/polypeptide 1	-1.81	0.0004
Aph1b	APH1B gamma secretase subunit	-1.81	0.0028
Ptger3	prostaglandin E receptor 3 (subtype EP3)	-1.81	0.0163
F11	coagulation factor XI	-1.81	0.0001
Fam117b	family with sequence similarity 117, member B	-1.81	0.0036
Spice1	spindle and centriole associated protein 1	-1.82	0.0053
Cyp7b1	cytochrome P450, family 7, subfamily b, polypeptide 1	-1.82	0.0434
Prkaa2	protein kinase, AMP-activated, alpha 2 catalytic subunit	-1.82	0.0006
Zfp57	zinc finger protein 57	-1.83	0.0266
Agt	angiotensinogen (serpin peptidase inhibitor, clade A, member 8)	-1.83	0.0003
Mreg	melanoregulin	-1.83	0.0001
Tbc1d14	TBC1 domain family, member 14	-1.84	0.0002
Siglech	sialic acid binding Ig-like lectin H [Source:RGD Symbol;Acc:1310401]	-1.85	0.0022
Dab1	Dab, reelin signal transducer, homolog 1 (Drosophila)	-1.85	0.0169
Gsap	gamma-secretase activating protein	-1.85	0.0010
Phtf1	putative homeodomain transcription factor 1	-1.86	0.0001
LOC100910501	ENCODES a protein that exhibits aminopeptidase activity (ortholog)	-1.86	0.0114
Tstd1	thiosulfate sulfurtransferase (rhodanese)-like domain containing 1	-1.86	0.0014
LOC299282	Serine protease inhibitor	-1.87	0.0018
RGD1566035	similar to protein tyrosine phosphatase 4a1	-1.87	0.0040
LOC102552246	uncharacterized LOC102552246	-1.88	0.0046
LOC681366	ENCODES a protein that exhibits oxidoreductase activity (inferred)	-1.88	0.0040
Aox4	aldehyde oxidase 4	-1.89	0.0001
Car8	carbonic anhydrase 8	-1.90	0.0001
Slc25a15	solute carrier family 25 (mitochondrial carrier; ornithine transporter) member 15	-1.90	0.0033
Dnajb9	DnaJ (Hsp40) homolog, subfamily B, member 9	-1.90	0.0179
Hacl1	2-hydroxyacyl-CoA lyase 1	-1.90	0.0303
Itga6	integrin, alpha 6	-1.90	0.0071
LOC500124	similar to RIKEN cDNA 4921507P07	-1.92	0.0170
Tob1	transducer of ErbB-2.1	-1.92	0.0135
Meiob	meiosis specific with OB domains	-1.92	0.0228
Sat2	spermidine/spermine N1-acetyltransferase family member 2	-1.92	0.0073
Foxp2	forkhead box P2	-1.93	0.0342
Casp6	caspase 6 (Casp6), transcript variant 2, mRNA.	-1.93	0.0057
RGD1309362	similar to interferon-inducible GTPase	-1.94	0.0233
Pdgfc	platelet derived growth factor C	-1.94	0.0010

Rup2	urinary protein 2	-1.94	0.0000
Dio1	deiodinase, iodothyronine, type I	-1.94	0.0058
Efna5	ephrin A5	-1.96	0.0096
Sox5	SRY (sex determining region Y)-box 5	-1.96	0.0002
Plekhb1	pleckstrin homology domain containing, family B (evectins) member 1	-1.97	0.0129
LOC102549198	interferon-inducible GTPase 1-like	-1.97	0.0406
LOC102549203	uncharacterized LOC102549203	-1.98	0.0018
RGD1565590	INTERACTS WITH thioacetamide	-1.99	0.0450
Sh3bp5	SH3-domain binding protein 5 (BTK-associated)	-1.99	0.0014
LOC102549464	TBC1 domain family member 14-like	-2.00	0.0005
ENSMUSG00000028542	solute carrier family 6 (neurotransmitter transporter, glycine), member 9, Non-coding transcript identified by NONCODE: Antisense	-2.01	0.0001
Cdh2	cadherin 2	-2.01	0.0005
Cyp2e1	cytochrome P450, family 2, subfamily e, polypeptide 1	-2.01	0.0083
Pdzd4	PDZ domain containing 4	-2.01	0.0019
Hyls1	hydrolethalus syndrome 1	-2.01	0.0190
Apon	apolipoprotein N	-2.02	0.0005
Acsl4	acyl-CoA synthetase long-chain family member 4	-2.03	0.0031
Enpep	glutamyl aminopeptidase	-2.03	0.0025
Dtx3l	deltex 3 like, E3 ubiquitin ligase	-2.03	0.0000
Pid1	phosphotyrosine interaction domain containing 1	-2.03	0.0071
Aph1b	APH1B gamma secretase subunit (Aph1b), mRNA	-2.04	0.0037
Zfp37	zinc finger protein 37	-2.05	0.0008
Crym	crystallin, mu	-2.05	0.0075
Napepld	N-acyl phosphatidylethanolamine phospholipase D	-2.05	0.0201
Sdr9c7	short chain dehydrogenase/reductase family 9C, member 7	-2.06	0.0004
Asgr1	asialoglycoprotein receptor 1	-2.07	0.0000
Ncam2	neural cell adhesion molecule 2	-2.07	0.0005
Pvrl3	poliovirus receptor-related 3	-2.08	0.0000
Atxn7l1	ataxin 7-like 1	-2.09	0.0280
Cers6	ceramide synthase 6	-2.10	0.0002
Afm	afamin	-2.10	0.0000
C1r	complement component 1, r subcomponent	-2.11	0.0002
Inmt	indolethylamine N-methyltransferase	-2.12	0.0151
RGD1566035	Protein tyrosine phosphatase type IVA 1	-2.12	0.0011
Ficd	FIC domain containing	-2.13	0.0265
Serpina3c	serine (or cysteine) proteinase inhibitor, clade A, member 3C	-2.13	0.0061
Papss2	3-phosphoadenosine 5-phosphosulfate synthase 2	-2.14	0.0008
Nr3c1	nuclear receptor subfamily 3, group C, member 1	-2.15	0.0000
Irak3	interleukin-1 receptor-associated kinase 3	-2.19	0.0051
Rnf144b	ring finger protein 144B	-2.20	0.0007
Sts	steroid sulfatase (microsomal), isozyme S	-2.20	0.0000
Esrrg	estrogen-related receptor gamma	-2.20	0.0003
Casp6	caspase 6	-2.21	0.0011
RGD1564074	INTERACTS WITH lead diacetate AND aflatoxin B1 (ortholog) AND coumestrol (ortholog)	-2.22	0.0312
Gpld1	glycosylphosphatidylinositol specific phospholipase D1	-2.23	0.0003
Slc16a4	solute carrier family 16, member 4	-2.23	0.0284
Rad54l2	RAD54-like 2 (S. cerevisiae)	-2.23	0.0176
Crem	cAMP responsive element modulator	-2.25	0.0015
Mup5	major urinary protein 5	-2.26	0.0196
RGD1309362	similar to interferon-inducible GTPase	-2.27	0.0116
Ugp2	UDP-glucose pyrophosphorylase 2	-2.29	0.0403
Ptp4a1	protein tyrosine phosphatase type IVA, member 1	-2.29	0.0006
Pvrl2	poliovirus receptor-related 2	-2.29	0.0035
Amacr	alpha-methylacyl-CoA racemase	-2.30	0.0005
Mapkapk2	mitogen-activated protein kinase-activated protein kinase 2	-2.31	0.0013
Rad54l2	helicase ARIP4	-2.31	0.0007

Slc38a2	solute carrier family 38, member 2	-2.31	0.0004
LOC102549500	uncharacterized LOC102549500	-2.32	0.0020
Nnmt	nicotinamide N-methyltransferase	-2.33	0.0047
Pbld1	phenazine biosynthesis-like protein domain containing 1	-2.34	0.0033
Cyp2c12	cytochrome P450, family 2, subfamily c, polypeptide 12	-2.35	0.0221
Gys2	glycogen synthase 2	-2.35	0.0436
Tpst1	tyrosylprotein sulfotransferase 1	-2.36	0.0005
Parp9	poly (ADP-ribose) polymerase family, member 9	-2.37	0.0003
Csrp1	cysteine and glycine-rich protein 1	-2.37	0.0009
Xbp1	X-box binding protein 1	-2.37	0.0000
Acsm3	acyl-CoA synthetase medium-chain family member 3	-2.38	0.0026
Pck1	phosphoenolpyruvate carboxykinase 1 (soluble)	-2.39	0.0289
Rnf39	ring finger protein 39	-2.39	0.0010
Slc25a25	solute carrier family 25 (mitochondrial carrier, phosphate carrier), member 25	-2.40	0.0140
Agmo	alkylglycerol monooxygenase	-2.41	0.0000
Oaf	out at first homolog	-2.42	0.0094
Pdia5	protein disulfide isomerase family A, member 5	-2.42	0.0001
LOC100910057	sulfotransferase 1C2-like [Source:RGD Symbol;Acc:6500449]	-2.44	0.0104
Ces1c	carboxylesterase 1C	-2.44	0.0003
---	Protein LOC100911928	-2.46	0.0001
Net1	neuroepithelial cell transforming 1	-2.47	0.0044
Fmo3	flavin containing monooxygenase 3	-2.48	0.0000
Rcan1	regulator of calcineurin 1	-2.51	0.0000
Foxa1	forkhead box A1	-2.52	0.0001
Hamp	hepcidin antimicrobial peptide	-2.52	0.0022
Prr16	proline rich 16	-2.54	0.0000
Aox1	aldehyde oxidase 1	-2.54	0.0004
C1s	complement component 1, s subcomponent	-2.55	0.0062
Cml2	camello-like 2	-2.56	0.0059
Cml2	camello-like 2 (Cml2), mRNA	-2.56	0.0059
Oas1a	2-5 oligoadenylate synthetase 1A	-2.58	0.0307
Olr1353	olfactory receptor 1353	-2.58	0.0079
Wsb1	WD repeat and SOCS box-containing 1	-2.59	0.0058
Rtp3	receptor (chemosensory) transporter protein 3	-2.61	0.0039
Slc17a2	solute carrier family 17, member 2	-2.61	0.0000
LOC298111	alpha2u globulin	-2.62	0.0012
Tenm2	teneurin transmembrane protein 2	-2.67	0.0002
Hhex	hematopoietically expressed homeobox	-2.67	0.0069
RGD1307603	similar to hypothetical protein MGC37914	-2.69	0.0325
Asl	argininosuccinate lyase	-2.71	0.0094
Adamts5	ADAM metallopeptidase with thrombospondin type 1 motif, 5	-2.74	0.0137
Dpp6	dipeptidylpeptidase 6	-2.74	0.0064
LOC100362572	Mpv17 transgene, kidney disease mutant-like (predicted)-like	-2.75	0.0003
Chac1	ChaC glutathione-specific gamma-glutamylcyclotransferase 1	-2.78	0.0041
Cesl1	carboxylesterase-like 1	-2.79	0.0001
Slc46a1	solute carrier family 46 (folate transporter), member 1	-2.80	0.0001
Ror1	receptor tyrosine kinase-like orphan receptor 1	-2.81	0.0000
Tmem144	transmembrane protein 144	-2.87	0.0019
Abcc2	ATP-binding cassette, subfamily C (CFTR/MRP), member 2	-2.88	0.0002
Cyp3a9	cytochrome P450, family 3, subfamily a, polypeptide 9	-2.93	0.0025
Cpq	carboxypeptidase Q	-2.96	0.0000
---	Uncharacterized protein	-3.00	0.0020
Atp11c	ATPase, class VI, type 11C	-3.01	0.0001
RGD1562392	similar to Sulfotransferase K1 (rSULT1C2)	-3.05	0.0033
Hal	histidine ammonia lyase	-3.05	0.0011
LOC100363405	Zfp37 pseudogene	-3.07	0.0000
Irs2	insulin receptor substrate 2	-3.09	0.0032

Habp2	hyaluronan binding protein 2	-3.11	0.0000
Helz2	helicase with zinc finger 2, transcriptional coactivator	-3.11	0.0052
Pde4b	phosphodiesterase 4B, cAMP specific	-3.13	0.0015
Dusp1	dual specificity phosphatase 1	-3.17	0.0027
Lgsn	lengsin, lens protein with glutamine synthetase domain	-3.25	0.0014
Ifitm3	interferon induced transmembrane protein 3	-3.28	0.0003
Fam169b	family with sequence similarity 169, member B	-3.29	0.0029
LOC685067	similar to guanylate binding protein family, member 6	-3.35	0.0005
Smagp	small cell adhesion glycoprotein	-3.41	0.0005
Egfr	epidermal growth factor receptor	-3.47	0.0001
Ccnd1	cyclin D1	-3.48	0.0332
Eml5	echinoderm microtubule associated protein like 5	-3.48	0.0000
Gimd1	GIMAP family P-loop NTPase domain containing 1	-3.51	0.0119
Ces1d	carboxylesterase 1D	-3.52	0.0000
LOC366130	similar to Down syndrome critical region protein 3 homolog (Down syndrome critical region protein A homolog)	-3.72	0.0000
LOC100910526	ENCODES a protein that exhibits sulfotransferase activity (inferred) AND INVOLVED IN metabolic process (inferred)	-3.73	0.0005
Got1	glutamic-oxaloacetic transaminase 1, soluble	-3.85	0.0045
Lin7a	lin-7 homolog a (C. elegans)	-4.01	0.0001
RGD1565421	similar to Sulfotransferase K2 (rSULT1C2A) [Source:RGD Symbol;Acc:1565421]	-4.24	0.0024
RGD1565682	similar to lipase-like, ab-hydrolase domain containing 2	-4.43	0.0009
---	similar to Spleen protein 1 precursor (RSP-1) (LOC688335), mRNA	-4.62	0.0000
Rgn	regucalcin (senescence marker protein-30)	-4.64	0.0009
---	similar to Spleen protein 1 precursor (RSP-1) (LOC688335), mRNA	-4.80	0.0001
LOC688335	similar to Spleen protein 1 precursor (RSP-1)	-4.81	0.0000
Lin7a	ENCODES a protein that exhibits PDZ domain binding AND L27 domain binding (ortholog)	-4.85	0.0001
Cyp2c11	cytochrome P450, subfamily 2, polypeptide 11	-4.86	0.0000
UST4r	integral membrane transport protein UST4r	-4.94	0.0000
Cish	cytokine inducible SH2-containing protein	-5.04	0.0054
Zdhhc23	zinc finger, DHHC-type containing 23	-5.05	0.0003
A2ml1	alpha-2-macroglobulin-like 1	-5.07	0.0026
Marveld1	MARVEL domain containing 1	-5.10	0.0002
Sult1c2	sulfotransferase family 1C member 2	-5.54	0.0007
---	Uncharacterized protein	-6.12	0.0023
Fmo1	flavin containing monooxygenase 1	-6.14	0.0036
Amdhd1	amidohydrolase domain containing 1	-6.36	0.0003
Rdh16	retinol dehydrogenase 16 (all-trans)	-6.38	0.0000
Fabp7	fatty acid binding protein 7, brain	-6.44	0.0000
Gadd45g	growth arrest and DNA-damage-inducible, gamma	-7.26	0.0171
LOC298111	alpha2u globulin [Source:RGD Symbol;Acc:1593272]	-7.45	0.0010
Slc22a8	solute carrier family 22 (organic anion transporter), member 8	-7.81	0.0003
Onecut1	one cut homeobox 1	-7.88	0.0019
LOC100912610	major urinary protein-like [Source:RGD Symbol;Acc:6486015]	-9.31	0.0005
Nox4	NADPH oxidase 4	-10.82	0.0000
Hao2	hydroxyacid oxidase 2 (long chain)	-11.05	0.0000
Gfra1	GDNF family receptor alpha 1	-11.29	0.0000
Car3	carbonic anhydrase 3	-13.50	0.0001
Dhrs7	dehydrogenase/reductase (SDR family) member 7	-14.98	0.0000
LOC100910235	sulfotransferase 1C1-like [Source:RGD Symbol;Acc:6499500]	-15.60	0.0001
Sult1c3	sulfotransferase family, cytosolic, 1C, member 3	-16.33	0.0001
Socs2	suppressor of cytokine signaling 2	-16.56	0.0001
Nrep	neuronal regeneration related protein	-16.95	0.0000
Cyp3a18	cytochrome P450, family 3, subfamily a, polypeptide 18	-20.66	0.0000
Sds	serine dehydratase	-22.07	0.0108
LOC100360049	rCG64165-like	-46.12	0.0000

LOC100360095	urinary protein 1-like	-51.36	0.0000
LOC100912405	urinary protein 3-like	-57.62	0.0000
Cdh17	cadherin 17	-59.12	0.0000
Stac3	SH3 and cysteine rich domain 3	-173.24	0.0000
Acnat2	acyl-coenzyme A amino acid N-acyltransferase 2	-190.34	0.0000

Table S2 Fold change (FC) and *P*-value of all differentially expressed transcripts (FC > 1.3 or < -1.3, *P* < 0.05) between groups LE vs. LC

Gene Symbol	Gene Description	FC	*P*-value
Inmt	indolethylamine N-methyltransferase	2.47	0.0482
Olr886	ENCODES a protein that exhibits G-protein coupled receptor activity (inferred)	1.87	0.0056
Tcp11x2	t-complex 11 family, X-linked 2	1.79	0.0298
Tas2r116	taste receptor, type 2, member 116	1.70	0.0022
Ccl21	chemokine (C-C motif) ligand 21	1.69	0.0009
Id2	inhibitor of DNA binding 2	1.67	0.0201
Zfp69	zinc finger protein 69 [Source:RGD Symbol;Acc:2322990]	1.63	0.0384
Olr377	olfactory receptor 377	1.62	0.0162
MGC114246	similar to cathepsin R	1.62	0.0068
Lpcat2	lysophosphatidylcholine acyltransferase 2	1.60	0.0018
LOC689396	similar to RNA polymerase II elongation factor ELL2 [Source:RGD Symbol;Acc:1593468]	1.60	0.0069
Olr1084	olfactory receptor 1084	1.60	0.0164
RGD1308195	similar to secreted Ly6/uPAR related protein 2	1.59	0.0011
Zfp606	zinc finger protein 606	1.58	0.0189
Vom2r6	vomeronasal 2 receptor, 6	1.58	0.0055
Akap4	A kinase (PRKA) anchor protein 4	1.58	0.0130
Slc22a4	solute carrier family 22 (organic cation/zwitterion transporter), member 4	1.56	0.0276
Abca9	ATP-binding cassette, sub-family A (ABC1), member 9	1.56	0.0466
Jade2	jade family PHD finger 2	1.55	0.0192
Ppfibp2	PTPRF interacting protein, binding protein 2 (liprin beta 2)	1.55	0.0113
Vom2r49	vomeronasal 2 receptor, 49	1.55	0.0421
Grifin	galectin-related inter-fiber protein	1.55	0.0038
Lrrc39	leucine rich repeat containing 39	1.54	0.0013
Olr1653	olfactory receptor 1653	1.54	0.0321
1700016H13Rik	RIKEN cDNA 1700016H13 gene	1.53	0.0084
Olr821	olfactory receptor 821	1.53	0.0037
Ccdc152	coiled-coil domain containing 152	1.53	0.0100
Serpinb5	serpin peptidase inhibitor, clade B (ovalbumin), member 5	1.53	0.0060
Bmp1	bone morphogenetic protein 1	1.53	0.0010
LOC102549391	zinc finger protein 879-like	1.52	0.0004
Cabp2	calcium binding protein 2	1.51	0.0425
Olr824	olfactory receptor 824	1.50	0.0417
Akap14	A kinase (PRKA) anchor protein 14	1.50	0.0096
Zfp276	zinc finger protein (C2H2 type) 276	1.49	0.0364
LOC102555587	uncharacterized LOC102555587	1.49	0.0163
Olr468	olfactory receptor 468	1.49	0.0037
Afap1	actin filament associated protein 1	1.49	0.0121
Vom1r72	vomeronasal 1 receptor 72	1.48	0.0131
Pira2	Protein LOC100912456	1.48	0.0242
Scn8a	sodium channel, voltage gated, type VIII, alpha subunit	1.48	0.0235
Klra2	killer cell lectin-like receptor, subfamily A, member 2	1.48	0.0144
Prph	peripherin	1.47	0.0042
Ift88	intraflagellar transport 88	1.47	0.0368
Tex19.2	testis expressed gene 19.2	1.47	0.0126
RGD1305298	uncharacterized protein LOC299330	1.46	0.0143
Cdr2l	cerebellar degeneration-related protein 2-like	1.46	0.0337
Syn3	synapsin III	1.46	0.0076
Clcn6	chloride channel, voltage-sensitive 6	1.45	0.0221
Gucy1b2	guanylate cyclase 1, soluble, beta 2	1.45	0.0188
Olr453	olfactory receptor 453	1.45	0.0058
Olr305	olfactory receptor 305	1.44	0.0471

LOC102555616	uncharacterized LOC102555616	1.44	0.0002
Mrpl13	mitochondrial ribosomal protein L13	1.44	0.0321
Lrp2	low density lipoprotein receptor-related protein 2	1.44	0.0275
Apln	apelin	1.44	0.0441
Ddx60	DEAD (Asp-Glu-Ala-Asp) box polypeptide 60	1.44	0.0472
Ces2e	carboxylesterase 2E	1.44	0.0321
Zfp14	ZFP14 zinc finger protein	1.44	0.0203
Plk1	polo-like kinase 1	1.44	0.0078
Itgb3bp	Chalmel, et. al. AceView Annotation Itgb3bp.aSep08 Testis-expressed Unannotated Transcripts (TUTs)	1.43	0.0095
RGD1564865	similar to 20-alpha-hydroxysteroid dehydrogenase	1.43	0.0404
Ctxn2	cortexin 2	1.43	0.0153
Tec	tec protein tyrosine kinase	1.43	0.0047
Spz1	spermatogenic leucine zipper 1	1.43	0.0021
Tcta	T-cell leukemia translocation altered	1.43	0.0087
Mgam	PARTICIPATES IN galactose metabolic pathway AND starch and sucrose metabolic pathway	1.43	0.0304
Stard9	StAR-related lipid transfer domain containing 9	1.43	0.0386
Pira2	Protein LOC100912456	1.42	0.0233
Apcdd1	adenomatosis polyposis coli down-regulated 1	1.42	0.0260
Has2	hyaluronan synthase 2	1.42	0.0039
Coq5	coenzyme Q5, methyltransferase	1.41	0.0233
Bpgm	2,3-bisphosphoglycerate mutase	1.41	0.0423
Gpr82	G protein-coupled receptor 82	1.41	0.0458
RGD1566120	similar to novel protein [Source:RGD Symbol;Acc:1566120]	1.41	0.0492
Pdp2	pyruvate dehyrogenase phosphatase catalytic subunit 2	1.41	0.0045
RGD1564599	RGD1564599 (RGD1564599), mRNA	1.41	0.0488
Hk2	hexokinase 2	1.41	0.0290
Adat2	adenosine deaminase, tRNA-specific 2	1.41	0.0040
LOC102551365	stress response protein nst-1-like	1.41	0.0380
Olr224	olfactory receptor 224	1.41	0.0305
LOC102553026	INTERACTS WITH 17beta-estradiol (ortholog)	1.41	0.0417
NONMMUG026978	Non-coding transcript identified by NONCODE: Sense No Exonic	1.40	0.0261
Bambi	BMP and activin membrane-bound inhibitor	1.40	0.0253
Tceal7	transcription elongation factor A (SII)-like 7	1.40	0.0421
Olr1174	olfactory receptor 1174	1.40	0.0124
RGD1563104	similar to Vigilin (High density lipoprotein-binding protein)	1.40	0.0354
RGD1565252	Protein RGD1565252	1.40	0.0186
Ihh	Indian hedgehog	1.40	0.0229
LOC100910240	tyrosine-protein phosphatase non-receptor type 13-like	1.40	0.0426
Nabp1	nucleic acid binding protein 1	1.40	0.0231
Nuf2	NUF2, NDC80 kinetochore complex component	1.40	0.0265
Tmem114	transmembrane protein 114	1.40	0.0014
Adam4	a disintegrin and metalloprotease domain 4	1.39	0.0368
Eppk1	ENCODES a protein that exhibits poly(A) RNA binding (ortholog)	1.39	0.0150
Pacsin1	protein kinase C and casein kinase substrate in neurons 1	1.39	0.0109
Lck	LCK proto-oncogene, Src family tyrosine kinase	1.38	0.0021
Serpinb13	serpin peptidase inhibitor, clade B (ovalbumin), member 13	1.38	0.0128
Panx1	Pannexin 1	1.38	0.0437
Depdc1	DEP domain containing 1	1.38	0.0314
Rffl	ring finger and FYVE-like domain containing E3 ubiquitin protein ligase	1.38	0.0371
Tmem164	transmembrane protein 164	1.38	0.0148
Olr922	olfactory receptor 922	1.38	0.0328
Aif1	allograft inflammatory factor 1	1.38	0.0224
Nutm1	NUT midline carcinoma, family member 1	1.38	0.0321
Mrps28	mitochondrial ribosomal protein S28	1.38	0.0140

Slc34a1	solute carrier family 34 (type II sodium/phosphate cotransporter), member 1	1.38	0.0000
Vom2r21	INTERACTS WITH ammonium chloride	1.38	0.0022
Tmem125	transmembrane protein 125	1.38	0.0089
RGD1562885	similar to RIKEN cDNA 2300002M23	1.37	0.0275
RGD1304770	similar to Na+ dependent glucose transporter 1	1.37	0.0276
Rps15al1	ribosomal protein S15A-like 1 [Source:RGD Symbol;Acc:1596017]	1.37	0.0005
Olr707	olfactory receptor 707	1.37	0.0220
LOC691670	similar to natural killer cell protease 7	1.37	0.0079
Nostrin	nitric oxide synthase trafficking	1.37	0.0010
Scn10a	sodium channel, voltage-gated, type X, alpha subunit	1.37	0.0379
Zfp52	zinc finger protein 52	1.36	0.0196
G0s2	G0/G1switch 2	1.36	0.0398
Pag1	phosphoprotein membrane anchor with glycosphingolipid microdomains 1	1.36	0.0077
Sh3rf1	SH3 domain containing ring finger 1	1.36	0.0270
Serpina6	serpin peptidase inhibitor, clade A (alpha-1 antiproteinase, antitrypsin), member 6	1.36	0.0289
Cnksr3	Cnksr family member 3	1.36	0.0146
Ssc4d	scavenger receptor cysteine rich family, 4 domains	1.36	0.0149
Tfpt	TCF3 (E2A) fusion partner	1.36	0.0204
Scp2d1	SCP2 sterol-binding domain containing 1	1.36	0.0236
Drc3	dynein regulatory complex subunit 3	1.36	0.0249
Ptpn12	protein tyrosine phosphatase, non-receptor type 12	1.36	0.0360
Ugt2a1	UDP glucuronosyltransferase 2 family, polypeptide A1 [Source:RGD Symbol;Acc:69432]	1.36	0.0294
Itih5	inter-alpha (globulin) inhibitor H5	1.36	0.0078
Slc25a29	solute carrier family 25 (mitochondrial carnitine/acylcarnitine carrier), member 29	1.36	0.0140
Lrig2	leucine-rich repeats and immunoglobulin-like domains 2	1.36	0.0456
RGD1564854	similar to divalent cation tolerant protein CUTA	1.35	0.0034
Rara	retinoic acid receptor, alpha	1.35	0.0473
Mylk	myosin light chain kinase (Mylk), mRNA. Chalmel, et. al. Testis-expressed Unannotated Transcripts (TUTs)	1.35	0.0413
Pcnxl4	pecanex-like 4 (Drosophila) [Source:RGD Symbol;Acc:1582892]	1.35	0.0119
Fuk	fucokinase	1.35	0.0051
Cd300lf	Cd300 molecule-like family member F	1.35	0.0103
Mllt1	myeloid/lymphoid or mixed-lineage leukemia; translocated to, 1	1.35	0.0174
Oxgr1	oxoglutarate (alpha-ketoglutarate) receptor 1	1.35	0.0315
Nab2	Ngfi-A binding protein 2	1.35	0.0056
Ankrd66	ankyrin repeat domain 66 [Source:RGD Symbol;Acc:1560151]	1.35	0.0128
Ogdhl	oxoglutarate dehydrogenase-like	1.35	0.0067
Cd69	Cd69 molecule	1.35	0.0105
Fyb	FYN binding protein	1.35	0.0092
Rnf11	ENCODES a protein that exhibits ubiquitin-protein transferase activity (ortholog)	1.35	0.0138
Rnf11l2	INTERACTS WITH 2-methoxyethanol	1.35	0.0138
Slc6a12	solute carrier family 6 (neurotransmitter transporter), member 12	1.35	0.0210
Hist1h2ah	histone cluster 1, H2ah [Source:RGD Symbol;Acc:1594367]	1.34	0.0219
Hspa1a	heat shock 70kD protein 1A	1.34	0.0111
Sh3kbp1	SH3-domain kinase binding protein 1	1.34	0.0020
Bpifa1	BPI fold containing family A, member 1	1.34	0.0135
RGD1305422	similar to mKIAA0226 protein	1.34	0.0286
RGD1566355	similar to cell division cycle 2-like 1	1.34	0.0463
Soga1	Protein Soga1	1.34	0.0119
Taf1b	TATA box binding protein (Tbp)-associated factor, RNA polymerase I, B	1.34	0.0048
Chchd6	coiled-coil-helix-coiled-coil-helix domain containing 6	1.34	0.0388

Fhit	Chalmel, et. al. AceView Annotation Fhit.bSep08 Testis-expressed Unannotated Transcripts (TUTs)	1.34	0.0297
Kctd2	potassium channel tetramerization domain containing 2 [Source:RGD Symbol;Acc:1566063]	1.34	0.0430
Syndig1	synapse differentiation inducing 1	1.33	0.0170
Mtif2	mitochondrial translational initiation factor 2	1.33	0.0335
Kif2a	kinesin heavy chain member 2A	1.33	0.0358
Tyrobp	Tyro protein tyrosine kinase binding protein	1.33	0.0443
Rbm8a	RNA binding motif protein 8A	1.33	0.0173
Best4	bestrophin 4	1.33	0.0220
Uroc1	urocanate hydratase 1	1.33	0.0139
Dner	delta/notch-like EGF repeat containing	1.33	0.0296
Cers5	ceramide synthase 5	1.33	0.0378
LOC102555347	uncharacterized LOC102555347	1.33	0.0079
Pcdhgc5	protocadherin gamma subfamily C, 5	1.33	0.0291
Hmha1	histocompatibility (minor) HA-1	1.33	0.0358
S100a4	S100 calcium-binding protein A4	1.33	0.0168
Tap2	transporter 2, ATP-binding cassette, sub-family B (MDR/TAP)	1.33	0.0190
Dll1	delta-like 1 (Drosophila)	1.33	0.0447
Syngap1	synaptic Ras GTPase activating protein 1	1.33	0.0248
Card11	caspase recruitment domain family, member 11	1.32	0.0131
Cbx2	chromobox homolog 2	1.32	0.0015
LOC100909631	ENCODES a protein that exhibits structural molecule activity (inferred)	1.32	0.0096
Olr1562	ENCODES a protein that exhibits G-protein coupled receptor activity (inferred)	1.32	0.0273
Klhl8	kelch-like family member 8	1.32	0.0148
Kif4a	kinesin family member 4A	1.32	0.0276
Npap60	nuclear pore associated protein [Source:RGD Symbol;Acc:3191]	1.32	0.0496
Tmem25	transmembrane protein 25	1.32	0.0153
Sirpb2l1	INTERACTS WITH 17beta-estradiol (ortholog)	1.32	0.0314
Tmem253	transmembrane protein 253	1.32	0.0235
Grpr	gastrin releasing peptide receptor	1.32	0.0040
LOC100360700	hypothetical LOC100360700	1.32	0.0472
Cacna1b	calcium channel, voltage-dependent, N type, alpha 1B subunit	1.32	0.0141
RGD1560927	RGD1560927	1.32	0.0092
RGD1564657	similar to cathepsin 1 precursor	1.32	0.0472
Wnt2b	wingless-type MMTV integration site family, member 2B	1.32	0.0345
Dapp1	dual adaptor of phosphotyrosine and 3-phosphoinositides	1.32	0.0465
Ccdc66	coiled-coil domain containing 66	1.32	0.0226
Slc12a6	solute carrier family 12, member 6	1.32	0.0258
Rbm41	RNA binding motif protein 41	1.32	0.0314
Cabp1	calcium binding protein 1	1.32	0.0256
Morc1	MORC family CW-type zinc finger 1	1.32	0.0193
Tmem35	transmembrane protein 35	1.32	0.0218
Kcnn3	potassium channel, calcium activated intermediate/small conductance subfamily N alpha, member 3	1.32	0.0151
Atp8b5p	ATPase, class I, type 8B, member 5, pseudogene	1.32	0.0236
Fcgr2a	Fc fragment of IgG, low affinity IIa, receptor	1.31	0.0484
Nr1h5	nuclear receptor subfamily 1, group H, member 5	1.31	0.0305
Gpcpd1	glycerophosphocholine phosphodiesterase 1	1.31	0.0236
Atp1a2	ATPase, Na+/K+ transporting, alpha 2 polypeptide	1.31	0.0009
Krt8	keratin 8, type II	1.31	0.0184
Trex2	three prime repair exonuclease 2	1.31	0.0283
Tbca	tubulin folding cofactor A [Source:RGD Symbol;Acc:1311538]	1.31	0.0488
Prima1	proline rich membrane anchor 1	1.31	0.0088
Anp32a	acidic (leucine-rich) nuclear phosphoprotein 32 family, member A	1.31	0.0266
Dlx4	distal-less homeobox 4	1.31	0.0366

RGD1562844	INTERACTS WITH cisplatin	-1.31	0.0302
Zswim8	zinc finger, SWIM-type containing 8	-1.31	0.0242
---	Uncharacterized protein	-1.31	0.0100
Pik3ip1	phosphoinositide-3-kinase interacting protein 1	-1.31	0.0344
Zfp84	zinc finger protein 84	-1.31	0.0202
Poglut1	protein O-glucosyltransferase 1	-1.31	0.0421
LOC102546793	uncharacterized LOC102546793	-1.31	0.0314
Foxr1	forkhead box R1 [Source:RGD Symbol;Acc:1309739]	-1.31	0.0357
Zbtb48	zinc finger and BTB domain containing 48	-1.31	0.0149
Mcoln2	mucolipin 2	-1.31	0.0381
Rab3b	RAB3B, member RAS oncogene family	-1.32	0.0384
Zfp773	zinc finger protein 773 [Source:MGI Symbol;Acc:MGI:1923623]	-1.32	0.0038
Clec2l	C-type lectin domain family 2, member L	-1.32	0.0061
Hoxc4	homeo box C4	-1.32	0.0216
Olr1768	olfactory receptor 1768	-1.32	0.0482
Bcl11b	B-cell CLL/lymphoma 11B (zinc finger protein) (Bcl11b), mRNA. Chalmel, et. al. Testis-expressed Unannotated Transcripts (TUTs)	-1.32	0.0319
Ttc8	tetratricopeptide repeat domain 8	-1.32	0.0487
Tut1	terminal uridylyl transferase 1, U6 snRNA-specific	-1.32	0.0426
LOC690035	similar to Protein KIAA0586	-1.32	0.0062
Cel	carboxyl ester lipase	-1.32	0.0324
Olr1451	ENCODES a protein that exhibits olfactory receptor activity (inferred)	-1.32	0.0106
LOC103691893	putative ERV-F(c)1 provirus ancestral Env polyprotein	-1.32	0.0425
Gpatch8	G patch domain containing 8	-1.32	0.0452
Cxcr3	chemokine (C-X-C motif) receptor 3	-1.32	0.0426
Wdsub1	WD repeat, sterile alpha motif and U-box domain containing 1	-1.32	0.0160
Cdkl4	cyclin-dependent kinase-like 4 [Source:RGD Symbol;Acc:1583704]	-1.32	0.0227
Popdc3	popeye domain containing 3	-1.32	0.0431
Smn1	survival of motor neuron 1, telomeric	-1.32	0.0126
Tgfbr3	transforming growth factor, beta receptor III	-1.32	0.0396
Slc24a1	solute carrier family 24 (sodium/potassium/calcium exchanger), member 1	-1.33	0.0402
Blk	BLK proto-oncogene, Src family tyrosine kinase	-1.33	0.0448
Ston1	stonin 1	-1.33	0.0085
Ndufa4l2	NADH dehydrogenase (ubiquinone) 1 alpha subcomplex, 4-like 2	-1.33	0.0086
Slc50a1	solute carrier family 50 (sugar efflux transporter), member 1	-1.33	0.0227
Tspan33	tetraspanin 33	-1.33	0.0351
Lrrc14	leucine rich repeat containing 14	-1.33	0.0061
Cdx4	caudal type homeo box 4	-1.33	0.0012
Sapcd1	suppressor APC domain containing 1	-1.33	0.0457
S100a11	S100 calcium binding protein A11	-1.33	0.0425
LOC685152	ATPase, class I, type 8B, member 2	-1.33	0.0042
Pold2	polymerase (DNA directed), delta 2, accessory subunit	-1.33	0.0316
Tmem39a	transmembrane protein 39a	-1.33	0.0336
Upk1b	uroplakin 1B	-1.33	0.0361
Ankmy1	ankyrin repeat and MYND domain containing 1	-1.33	0.0372
Cml1	camello-like 1 (Cml1), mRNA	-1.34	0.0186
Dnah2	Protein Dnah2	-1.34	0.0324
RGD1564651	similar to oocyte specific homeobox 3 [Source:RGD Symbol;Acc:1564651]	-1.34	0.0297
Miip	migration and invasion inhibitory protein	-1.34	0.0421
Tfb2m	ENCODES a protein that exhibits poly(A) RNA binding (ortholog)	-1.34	0.0214
Tas2r134	taste receptor, type 2, member 134	-1.34	0.0123
Sfmbt1	Scm-like with four mbt domains 1	-1.34	0.0379
RGD1559482	similar to immunoglobulin superfamily, member 7	-1.34	0.0143
Olr312	olfactory receptor 312	-1.34	0.0357
Rbbp9	retinoblastoma binding protein 9	-1.34	0.0440

Wasf2	WAS protein family, member 2	-1.34	0.0298
---	---	-1.34	0.0092
Prrt3	proline-rich transmembrane protein 3	-1.34	0.0180
Sh3d21	SH3 domain containing 21	-1.34	0.0413
Tubb4a	tubulin, beta 4A class IVa	-1.34	0.0276
Vwf	von Willebrand factor	-1.34	0.0365
Ly6h	lymphocyte antigen 6 complex, locus H	-1.35	0.0133
Akap3	A kinase (PRKA) anchor protein 3	-1.35	0.0072
LOC498084	Ab2-093	-1.35	0.0106
Plekhh2	pleckstrin homology domain containing, family H (with MyTH4 domain) member 2	-1.35	0.0044
Rnase10	ribonuclease, RNase A family, 10 (non-active)	-1.35	0.0427
Olr129	olfactory receptor 129	-1.35	0.0381
Cnga2	cyclic nucleotide gated channel alpha 2	-1.35	0.0250
Tspan1	tetraspanin 1	-1.35	0.0097
Ighmbp2	immunoglobulin mu binding protein 2	-1.35	0.0181
Ccdc185	coiled-coil domain containing 185	-1.35	0.0102
Galns	galactosamine (N-acetyl)-6-sulfatase	-1.35	0.0401
Bcl2	B-cell CLL/lymphoma 2	-1.35	0.0010
Entpd1	ENCODES a protein that exhibits nucleoside-diphosphatase activity (ortholog)	-1.35	0.0079
Snai1	snail family zinc finger 1	-1.35	0.0484
Gpr65	G-protein coupled receptor 65	-1.36	0.0236
Tox	thymocyte selection-associated high mobility group box	-1.36	0.0246
LOC102555390	uncharacterized LOC102555390	-1.36	0.0256
Banp	Btg3 associated nuclear protein	-1.36	0.0268
Mboat7	membrane bound O-acyltransferase domain containing 7 (Mboat7), mRNA	-1.36	0.0480
Mtx3	metaxin 3	-1.36	0.0336
Tktl2	transketolase-like 2	-1.36	0.0251
Zfp367	zinc finger protein 367	-1.36	0.0140
RGD1308878	similar to arylacetamide deacetylase	-1.36	0.0076
Cysltr1	cysteinyl leukotriene receptor 1	-1.36	0.0103
Fuz	fuzzy planar cell polarity protein	-1.36	0.0048
Mecom	MDS1 and EVI1 complex locus	-1.36	0.0245
Il2ra	interleukin 2 receptor, alpha	-1.37	0.0443
Jag2	jagged 2 [Source:RGD Symbol;Acc:2938]	-1.37	0.0160
Tbata	thymus, brain and testes associated	-1.37	0.0238
Ehd3	EH-domain containing 3	-1.37	0.0269
Gpr55	G protein-coupled receptor 55	-1.37	0.0198
LOC100362774	rCG64303-like [Source:RGD Symbol;Acc:2324084]	-1.37	0.0173
Pkmyt1	protein kinase, membrane associated tyrosine/threonine 1	-1.37	0.0297
Iqcf1	IQ motif containing F1	-1.37	0.0038
Ankrd63	ankyrin repeat domain 63	-1.37	0.0151
Rabif	RAB interacting factor [Source:RGD Symbol;Acc:1359331]	-1.37	0.0234
RGD1564941	similar to ankyrin repeat domain 26	-1.37	0.0353
Bgn	biglycan	-1.38	0.0343
Olr1314	olfactory receptor 1314	-1.38	0.0471
Olr1314	olfactory receptor 1314 (Olr1314), mRNA	-1.38	0.0471
Olr358	olfactory receptor 358	-1.38	0.0210
Dpysl2	dihydropyrimidinase-like 2	-1.38	0.0062
Olr1399	olfactory receptor 1399	-1.38	0.0452
LOC691254	hypothetical protein LOC691254	-1.38	0.0162
LOC100361265	rCG64283-like	-1.38	0.0367
Vom2r24	vomeronasal 2 receptor, 24	-1.38	0.0175
Entpd1	ectonucleoside triphosphate diphosphohydrolase 1	-1.38	0.0039
Fsd2	fibronectin type III and SPRY domain containing 2	-1.38	0.0143
LOC102553221	MLV-related proviral Env polyprotein-like	-1.38	0.0200

Olr1247	ENCODES a protein that exhibits olfactory receptor activity (inferred)	-1.39	0.0033
Olr1247	olfactory receptor 1247	-1.39	0.0033
Rgr	retinal G protein coupled receptor	-1.39	0.0017
Mmp3	matrix metallopeptidase 3	-1.39	0.0400
Strn3	Striatin-3 [Source:UniProtKB/Swiss-Prot;Acc:P58405] Chalmel, et. al. Testis-expressed Unannotated Transcripts (TUTs)	-1.39	0.0455
Dock7	dedicator of cytokinesis 7	-1.39	0.0174
Nkain2	Na+/K+ transporting ATPase interacting 2 [Source:RGD Symbol;Acc:2323702]	-1.39	0.0229
LOC100361579	prolactin family 5 subfamily a member 2-like	-1.39	0.0268
Phf19	PHD finger protein 19	-1.40	0.0276
Myo5a	myosin VA	-1.40	0.0132
Vom2r63	vomeronasal 2 receptor, 63	-1.40	0.0316
Rad54l	RAD54 like (S. cerevisiae)	-1.40	0.0134
Stk11ip	serine/threonine kinase 11 interacting protein	-1.40	0.0328
Fzd3	frizzled class receptor 3	-1.40	0.0258
Ankrd33b	ankyrin repeat domain 33B	-1.40	0.0094
Hsf1	heat shock transcription factor 1	-1.41	0.0079
Maged2	melanoma antigen, family D, 2	-1.41	0.0023
Ric3	RIC3 acetylcholine receptor chaperone	-1.41	0.0424
Olr330	olfactory receptor 330	-1.41	0.0236
Tle6	transducin-like enhancer of split 6 [Source:RGD Symbol;Acc:1561530]	-1.41	0.0015
Vom1r100	vomeronasal 1 receptor 100	-1.41	0.0193
Rabif	RAB interacting factor	-1.41	0.0261
U2af1l4	U2 small nuclear RNA auxiliary factor 1-like 4	-1.42	0.0062
Barx1	BARX homeobox 1	-1.42	0.0274
Ctnnal1	catenin (cadherin associated protein), alpha-like 1	-1.42	0.0213
Olr639	olfactory receptor 639	-1.42	0.0254
Olr639	olfactory receptor 639 (Olr639), mRNA	-1.42	0.0254
Olr639	olfactory receptor 639 (Olr639), mRNA	-1.42	0.0254
Tmtc2	transmembrane and tetratricopeptide repeat containing 2	-1.42	0.0334
Arsb	arylsulfatase B	-1.42	0.0111
LOC100361702	keratin associated protein 12-1-like	-1.42	0.0459
Nrxn3	neurexin 3	-1.42	0.0000
Olr606	olfactory receptor 606	-1.43	0.0298
RGD1311343	similar to RIKEN cDNA 4930524B15	-1.43	0.0036
Zfp329	zinc finger protein 329	-1.43	0.0224
Olr657	olfactory receptor 657	-1.43	0.0445
Smim15	small integral membrane protein 15	-1.43	0.0028
LOC102547422	zinc finger protein 420-like	-1.43	0.0206
Rsl24d1	ribosomal L24 domain containing 1	-1.43	0.0058
Tmeff2	transmembrane protein with EGF-like and two follistatin-like domains 2	-1.43	0.0165
Lipe	lipase, hormone sensitive	-1.43	0.0054
Olfml2a	olfactomedin-like 2A	-1.43	0.0103
---	---	-1.43	0.0408
Grm3	glutamate receptor, metabotropic 3	-1.43	0.0132
---	---	-1.44	0.0078
LOC100360157	1110034B05Rik protein-like	-1.44	0.0124
Dynlt1	dynein light chain Tctex-type 1	-1.44	0.0052
Ns5atp9	NS5A (hepatitis C virus) transactivated protein 9	-1.45	0.0233
Wdr44	WD repeat domain 44	-1.45	0.0314
Nlrp12	NLR family, pyrin domain containing 12	-1.45	0.0238
Lifr	leukemia inhibitory factor receptor alpha	-1.45	0.0051
NONMMUG023495	Non-coding transcript identified by NONCODE: Sense No Exonic	-1.45	0.0321
Antxr1	anthrax toxin receptor 1	-1.45	0.0088

Ntng1	netrin G1	-1.45	0.0333
Orai2	ORAI calcium release-activated calcium modulator 2	-1.45	0.0361
LOC102553180	protocadherin alpha-1-like	-1.46	0.0330
Krtap31-1	keratin associated protein 31-1	-1.46	0.0216
Cdcp2	CUB domain containing protein 2 [Source:RGD Symbol;Acc:1563467]	-1.46	0.0222
Olr510	olfactory receptor 510	-1.46	0.0055
ENSMUSG00000028542	solute carrier family 6 (neurotransmitter transporter, glycine), member 9, Non-coding transcript identified by NONCODE: Antisense	-1.46	0.0118
Cd96	CD96 molecule	-1.46	0.0045
Fv1	Friend virus susceptibility 1	-1.46	0.0004
LOC100362122	selection and upkeep of intraepithelial T cells 3-like	-1.47	0.0191
Fam46a	family with sequence similarity 46, member A	-1.47	0.0147
LOC103694398	keratin-associated protein 10-1-like	-1.49	0.0009
LOC100912937	melanoma antigen preferentially expressed in tumors-like [Source:RGD Symbol;Acc:6485206]	-1.49	0.0008
Pcdhb9	protocadherin beta 9	-1.49	0.0196
Ogn	osteoglycin	-1.49	0.0131
Cdc14a	cell division cycle 14A	-1.49	0.0385
Frem3	FRAS1 related extracellular matrix 3	-1.50	0.0173
Olr718	olfactory receptor 718	-1.50	0.0465
RGD1561444	similar to RIKEN cDNA 9530077C05 [Source:RGD Symbol;Acc:1561444]	-1.50	0.0169
Dnah5	Protein Dnah5	-1.50	0.0393
Zscan21	zinc finger and SCAN domain containing 21	-1.50	0.0024
Sbp	spermine binding protein	-1.50	0.0255
LOC102554689	uncharacterized LOC102554689	-1.50	0.0216
Mcm7	minichromosome maintenance complex component 7	-1.51	0.0062
LOC100910689	cytochrome c oxidase assembly protein COX11, mitochondrial-like [Source:RGD Symbol;Acc:6497536]	-1.51	0.0034
RGD1584023	similar to zinc finger protein 11B	-1.51	0.0018
Olr1437	olfactory receptor 1437	-1.52	0.0500
Ceacam15	carcinoembryonic antigen-related cell adhesion molecule 15 [Source:MGI Symbol;Acc:MGI:2141810]	-1.53	0.0393
LOC688657	similar to Olfactory receptor 5D13	-1.53	0.0103
Olr1697	olfactory receptor 1697	-1.54	0.0141
Il2rg	interleukin 2 receptor, gamma	-1.55	0.0225
Pde6d	phosphodiesterase 6D, cGMP-specific, rod, delta	-1.57	0.0104
LOC102553768	uncharacterized LOC102553768	-1.57	0.0091
Olr74	olfactory receptor 74	-1.60	0.0323
Rnf212	INVOLVED IN chiasma assembly (ortholog)	-1.61	0.0036
Mgat4e	MGAT4 family, member E	-1.62	0.0008
RGD1561778	similar to dendritic cell-derived immunoglobulin(Ig)-like receptor 1, DIgR1 - mouse	-1.63	0.0063
Nuggc	nuclear GTPase, germinal center associated	-1.64	0.0013
LOC102551428	uncharacterized LOC102551428	-1.69	0.0027
Pdcl2	phosducin-like 2	-1.69	0.0229
Mansc1	MANSC domain containing 1	-1.71	0.0069
LOC102546754	beclin 1-associated autophagy-related key regulator-like	-1.72	0.0074
Per1	period circadian clock 1	-1.98	0.0144
Tsku	tsukushi, small leucine rich proteoglycan	-2.95	0.0045

Table S3 Fold change (FC) and *P*-value of all differentially expressed transcripts (FC > 1.3 or < -1.3, *P* < 0.05) between groups OE vs. OC

Gene Symbol	Gene Description	FC	*P*-value
Gadd45a	growth arrest and DNA-damage-inducible, alpha	2.57	0.0208
Gimd1	GIMAP family P-loop NTPase domain containing 1	2.32	0.0240
Irs2	insulin receptor substrate 2	2.27	0.0043
Pdk4	pyruvate dehydrogenase kinase, isozyme 4	2.19	0.0225
Slc22a8	solute carrier family 22 (organic anion transporter), member 8	2.17	0.0450
LOC100911413	deleted in malignant brain tumors 1 protein-like	2.01	0.0070
Igkc	immunoglobulin kappa constant [Source:MGI Symbol;Acc:MGI:96495]	1.97	0.0109
Zfp467	zinc finger protein 467	1.88	0.0166
LOC102549464	TBC1 domain family member 14-like	1.86	0.0101
Junb	jun B proto-oncogene	1.83	0.0160
Eml5	echinoderm microtubule associated protein like 5	1.76	0.0074
Pid1	phosphotyrosine interaction domain containing 1	1.75	0.0014
Siglech	sialic acid binding Ig-like lectin H [Source:RGD Symbol;Acc:1310401]	1.74	0.0086
RGD1565806	similar to 60S ribosomal protein L23a	1.74	0.0047
Slc22a25	solute carrier family 22, member 25	1.72	0.0267
LOC100912707	Ig kappa chain V19-17-like [Source:RGD Symbol;Acc:6485939]	1.69	0.0334
Aim2	absent in melanoma 2	1.67	0.0067
NONMMUG023325	Non-coding transcript identified by NONCODE: Sense No Exonic	1.66	0.0100
Anxa1	annexin A1	1.66	0.0049
Spry2	sprouty RTK signaling antagonist 2	1.66	0.0118
Edaradd	EDAR-associated death domain	1.64	0.0196
LOC689303	similar to vitamin A-deficient testicular protein 11-like	1.64	0.0457
LOC100910120	olfactory receptor 8D1-like	1.63	0.0248
Wdr90	WD repeat domain 90	1.63	0.0037
LOC24906	RoBo-1	1.62	0.0348
Spag8	sperm associated antigen 8	1.62	0.0049
Fam57a	family with sequence similarity 57, member A	1.61	0.0256
Cyld	cylindromatosis (turban tumor syndrome) (Cyld), mRNA.	1.60	0.0115
---	Protein LOC502908	1.60	0.0230
LOC499136	LRRGT00021	1.59	0.0037
LOC102549637	filaggrin-2-like	1.59	0.0317
Sgol1	shugoshin-like 1 (S. pombe)	1.59	0.0108
RGD1565088	similar to UPF0197 protein C11orf10 homolog	1.58	0.0390
Anxa8	annexin A8	1.57	0.0498
Atad2	ATPase family, AAA domain containing 2	1.57	0.0125
Tmem159	transmembrane protein 159	1.57	0.0330
LOC100911032	uncharacterized LOC100911032	1.57	0.0168
Pkn3	protein kinase N3	1.57	0.0366
Isg20	interferon stimulated exonuclease gene 20	1.56	0.0089
Mc4r	melanocortin 4 receptor	1.56	0.0094
Gzmc	granzyme C	1.56	0.0014
RGD1563645	granzyme C (Gzmc), mRNA	1.56	0.0014
NONMMUG022196	Non-coding transcript identified by NONCODE: Exonic	1.56	0.0149
Gsap	gamma-secretase activating protein	1.55	0.0128
Nedd9	neural precursor cell expressed, developmentally down-regulated 9	1.55	0.0402
Pcdh15	protocadherin 15	1.55	0.0108
Net1	neuroepithelial cell transforming 1	1.55	0.0042
Clstn1	calsyntenin 1	1.55	0.0156
Unc79	INVOLVED IN adult behavior (ortholog)	1.55	0.0133

Tas2r120	taste receptor, type 2, member 120	1.54	0.0447
Polr2l	polymerase (RNA) II (DNA directed) polypeptide L (Polr2l), mRNA	1.54	0.0131
Csn1s1	casein alpha s1	1.54	0.0007
LOC681193	ENCODES a protein that exhibits DNA binding (inferred)	1.54	0.0036
Bcl2l11	BCL2-like 11 (apoptosis facilitator)	1.54	0.0224
Grk4	G protein-coupled receptor kinase 4	1.54	0.0052
Atf7	activating transcription factor 7	1.54	0.0003
Map1lc3a	microtubule-associated protein 1 light chain 3 alpha	1.54	0.0035
LOC102548427	uncharacterized LOC102548427	1.53	0.0476
Amigo2	adhesion molecule with Ig like domain 2	1.53	0.0341
Rcan1	regulator of calcineurin 1	1.53	0.0325
Leng8	leukocyte receptor cluster (LRC) member 8	1.53	0.0108
Olr281	olfactory receptor 281	1.53	0.0009
Esrrg	estrogen-related receptor gamma	1.52	0.0121
Tas2r143	ENCODES a protein that exhibits G-protein coupled receptor activity (inferred)	1.52	0.0161
Cers3	ceramide synthase 3	1.51	0.0285
Dock2	dedicator of cytokinesis 2 [Source:RGD Symbol;Acc:1564189]	1.51	0.0351
Nav1	neuron navigator 1	1.51	0.0446
LOC102552246	uncharacterized LOC102552246	1.51	0.0181
Irak3	interleukin-1 receptor-associated kinase 3	1.51	0.0384
Olr696	olfactory receptor 696	1.51	0.0198
Hmgb2l1	high mobility group box 2-like 1	1.50	0.0084
RGD1565590	INTERACTS WITH thioacetamide	1.50	0.0163
A2m	alpha-2-macroglobulin (A2m), mRNA	1.50	0.0301
LOC102551659	MLV-related proviral Env polyprotein-like	1.49	0.0274
LOC102554723	uncharacterized LOC102554723	1.49	0.0096
Zfp37	zinc finger protein 37	1.49	0.0452
Prr16	proline rich 16	1.49	0.0126
Nisch	nischarin [Source:RGD Symbol;Acc:1306950]	1.49	0.0055
Eda2r	ectodysplasin A2 receptor	1.48	0.0340
Zbtb42	zinc finger and BTB domain containing 42	1.48	0.0359
Fam168b	family with sequence similarity 168, member B	1.48	0.0161
Fndc3b	fibronectin type III domain containing 3B	1.48	0.0009
Abo	ABO blood group (transferase A, alpha 1-3-N-acetylgalactosaminyltransferase; transferase B, alpha 1-3-galactosyltransferase)	1.48	0.0231
LOC680097	similar to germinal histone H4 gene	1.48	0.0077
Rps27l	ribosomal protein S27-like	1.48	0.0224
Lst1	leukocyte specific transcript 1	1.47	0.0021
Nox4	NADPH oxidase 4	1.47	0.0481
Uvssa	UV-stimulated scaffold protein A	1.47	0.0253
Mib2	mindbomb E3 ubiquitin protein ligase 2	1.47	0.0079
LOC102556253	tudor domain-containing protein 15-like	1.47	0.0116
Scgb1c1	secretoglobin, family 1C, member 1	1.47	0.0403
Olr1505	olfactory receptor 1505	1.47	0.0028
Foxo3	forkhead box O3	1.47	0.0081
Vars2	valyl-tRNA synthetase 2, mitochondrial	1.46	0.0032
RGD1563104	similar to Vigilin (High density lipoprotein-binding protein)	1.46	0.0486
Fam81b	family with sequence similarity 81, member B	1.46	0.0270
Ddb2	damage specific DNA binding protein 2	1.46	0.0403
Tesb	testis specific basic protein	1.46	0.0125
LOC102546342	uncharacterized LOC102546342	1.46	0.0032
Xkrx	XK, Kell blood group complex subunit-related, X-linked	1.46	0.0209

Olr1653	olfactory receptor 1653	1.46	0.0267
Slc17a2	solute carrier family 17, member 2	1.46	0.0141
RGD1562035	INTERACTS WITH mercaptopurine AND purine-6-thiol AND benzo[a]pyrene (ortholog)	1.46	0.0210
Zfp692	zinc finger protein 692	1.45	0.0336
Cep112	centrosomal protein 112kDa	1.45	0.0037
Usp40	ubiquitin specific peptidase 40	1.45	0.0078
---	---	1.45	0.0375
Tpbpa	trophoblast specific protein alpha	1.45	0.0467
Olr67	olfactory receptor 67	1.45	0.0349
Arhgap15	Rho GTPase activating protein 15	1.45	0.0201
---	Protein LOC100362092	1.45	0.0256
LOC102548194	TBC1 domain family member 30-like	1.45	0.0226
LOC102548194	TBC1 domain family member 30-like	1.45	0.0226
Ddx17	DEAD (Asp-Glu-Ala-Asp) box helicase 17	1.45	0.0183
LOC102546322	COPII coat assembly protein sec16-like	1.45	0.0056
LOC310926	hypothetical protein LOC310926	1.44	0.0432
Uqcr11	ubiquinol-cytochrome c reductase, complex III subunit XI	1.44	0.0369
Mllt6	myeloid/lymphoid or mixed-lineage leukemia (trithorax homolog, Drosophila); translocated to, 6	1.44	0.0119
Clec2l	C-type lectin domain family 2, member L	1.44	0.0154
Lrrc23	leucine rich repeat containing 23	1.44	0.0245
Ccnl1	cyclin L1	1.44	0.0004
---	Protein Dnhd1-ps1	1.44	0.0158
Olr375	olfactory receptor 375	1.44	0.0225
Lyzl1	lysozyme-like 1	1.43	0.0169
Fgl1	fibrinogen-like 1	1.43	0.0112
Fam117b	family with sequence similarity 117, member B	1.43	0.0185
LOC102554977	INTERACTS WITH cisplatin (ortholog) AND progesterone (ortholog)	1.43	0.0382
LOC102549203	uncharacterized LOC102549203	1.43	0.0217
B3galt4	UDP-Gal:betaGlcNAc beta 1,3-galactosyltransferase, polypeptide 4	1.43	0.0488
Dgkh	diacylglycerol kinase, eta	1.43	0.0168
RGD1560556	similar to C14orf25 protein	1.42	0.0121
LOC688442	similar to limkain b1	1.42	0.0028
Vom2r52	vomeronasal 2 receptor, 52	1.42	0.0137
Vom2r27	vomeronasal 2 receptor, 27	1.42	0.0214
Mllt4	myeloid/lymphoid or mixed-lineage leukemia; translocated to, 4	1.42	0.0309
Klf12	Kruppel-like factor 12	1.42	0.0032
Anks1a	ankyrin repeat and sterile alpha motif domain containing 1A	1.42	0.0033
A2m	alpha-2-macroglobulin	1.42	0.0423
Stxbp5l	syntaxin binding protein 5-like	1.42	0.0375
Olr281	olfactory receptor 281 (Olr281), mRNA	1.42	0.0021
Cebpd	CCAAT/enhancer binding protein (C/EBP), delta	1.42	0.0401
Pafah1b3	platelet-activating factor acetylhydrolase 1b, catalytic subunit 3	1.42	0.0032
Polr2l	polymerase (RNA) II (DNA directed) polypeptide L	1.42	0.0345
LOC497940	similar to RIKEN cDNA 2810408A11	1.42	0.0048
RGD1560883	similar to KIAA0825 protein [Source:RGD Symbol;Acc:1560883]	1.42	0.0416
Olr300	olfactory receptor 300	1.42	0.0413
Fam219a	family with sequence similarity 219, member A	1.42	0.0492
RGD1310081	similar to hypothetical protein FLJ13231 [Source:RGD Symbol;Acc:1310081]	1.41	0.0237
Fos	FBJ osteosarcoma oncogene	1.41	0.0073
Olr1867	olfactory receptor 1867	1.41	0.0193
LOC100911112	uncharacterized LOC100911112	1.41	0.0009

LOC102550824	uncharacterized LOC102550824	1.41	0.0351
Dtwd1	DTW domain containing 1	1.41	0.0086
Adad2	adenosine deaminase domain containing 2	1.41	0.0011
Zp2	zona pellucida glycoprotein 2 (sperm receptor)	1.41	0.0480
Mxd1	max dimerization protein 1	1.41	0.0464
---	---	1.41	0.0010
Snrpc	small nuclear ribonucleoprotein polypeptide C	1.41	0.0145
Igfl3	IGF-like family member 3	1.41	0.0331
Olr1591	olfactory receptor 1591	1.41	0.0010
Rnf43	ring finger protein 43	1.41	0.0229
Olr70	olfactory receptor 70	1.41	0.0327
Traf3ip3	TRAF3 interacting protein 3	1.41	0.0301
Rab24	RAB24, member RAS oncogene family	1.41	0.0177
Olr1620	olfactory receptor 1620	1.41	0.0285
C5	complement component 5	1.41	0.0445
NONMMUG032780	Non-coding transcript identified by NONCODE: Linc	1.40	0.0265
LOC100363405	Zfp37 pseudogene	1.40	0.0137
Mcm4	minichromosome maintenance complex component 4	1.40	0.0271
Ndufa1	NADH dehydrogenase (ubiquinone) 1 alpha subcomplex, 1	1.40	0.0084
LOC681182	similar to paired immunoglobin-like type 2 receptor beta [Source:RGD Symbol;Acc:1583509]	1.40	0.0486
Syne4	spectrin repeat containing, nuclear envelope family member 4 [Source:RGD Symbol;Acc:1304580]	1.40	0.0446
Anlnl1	anillin, actin binding protein-like 1	1.40	0.0112
Tmem92	INTERACTS WITH 17beta-estradiol (ortholog) AND benzo[a]pyrene (ortholog) AND copper(2+) sulfate (ortholog)	1.40	0.0008
Olr763	ENCODES a protein that exhibits G-protein coupled receptor activity (inferred)	1.40	0.0171
Tmem258	INTERACTS WITH toluene AND cobalt dichloride (ortholog) AND copper(2+) sulfate (ortholog)	1.40	0.0050
Spi1	Spi-1 proto-oncogene	1.40	0.0261
Irf2bp2	interferon regulatory factor 2 binding protein 2	1.40	0.0135
Tcf7l2	transcription factor 7-like 2 (T-cell specific, HMG-box)	1.40	0.0085
Olr964	olfactory receptor 964	1.40	0.0004
LOC102548360	MLV-related proviral Env polyprotein-like	1.40	0.0335
Trak1	trafficking protein, kinesin binding 1	1.39	0.0442
Bdnf	brain-derived neurotrophic factor	1.39	0.0266
Dnah1	dynein, axonemal, heavy chain 1	1.39	0.0030
Prrt4	proline-rich transmembrane protein 4	1.39	0.0316
Zkscan4	Protein Zfp307	1.39	0.0477
Lrch4	leucine-rich repeats and calponin homology (CH) domain containing 4	1.39	0.0335
Ppp1r15a	protein phosphatase 1, regulatory subunit 15A	1.39	0.0167
Olr1500	olfactory receptor 1500	1.39	0.0071
Gpatch3	G patch domain containing 3	1.39	0.0450
Mef2d	myocyte enhancer factor 2D	1.39	0.0065
Fnbp4	formin binding protein 4	1.39	0.0479
Srsf5	serine/arginine-rich splicing factor 5	1.39	0.0374
LOC102548076	zinc finger protein 120-like	1.39	0.0284
Defb44	defensin beta 44	1.39	0.0082
Cep170b	centrosomal protein 170B	1.39	0.0177
Zfp28	zinc finger protein 28	1.39	0.0476
Cacna1a	calcium channel, voltage-dependent, P/Q type, alpha 1A subunit	1.39	0.0100
Olr1126	olfactory receptor 1126	1.39	0.0265

Arid5b	AT rich interactive domain 5B (Mrf1 like)	1.39	0.0360
Tmem110	transmembrane protein 110	1.39	0.0096
Tgm1	transglutaminase 1	1.39	0.0145
Ubp1	upstream binding protein 1 (LBP-1a)	1.39	0.0110
RGD1306091	PREDICTED: similar to Mixed lineage kinase 4 (RGD1306091), mRNA.	1.38	0.0017
Poldip3	polymerase (DNA-directed), delta interacting protein 3	1.38	0.0084
Fam168a	family with sequence similarity 168, member A	1.38	0.0204
Hoxa1	homeo box A1	1.38	0.0469
Rrp9	ribosomal RNA processing 9, small subunit (SSU) processome component, homolog (yeast)	1.38	0.0115
Zmat1	zinc finger, matrin-type 1	1.38	0.0428
LOC102552616	h-2 class I histocompatibility antigen TLA(B) alpha chain-like	1.38	0.0185
Phf13	PHD finger protein 13	1.38	0.0119
LOC100366231	X-linked lymphocyte-regulated 5C-like [Source:RGD Symbol;Acc:2324027]	1.38	0.0084
Eif2b4	eukaryotic translation initiation factor 2B, subunit 4 delta	1.38	0.0104
LOC102550998	sperm motility kinase 3-like	1.38	0.0248
RGD1309110	similar to Hypothetical protein MGC58999	1.38	0.0245
Cbx3	chromobox homolog 3	1.38	0.0202
Vegfa	vascular endothelial growth factor A	1.38	0.0352
Otp	orthopedia homeobox	1.38	0.0225
Ajap1	adherens junction associated protein 1	1.38	0.0304
Klre1	killer cell lectin-like receptor, family E, member 1	1.38	0.0307
LOC100909879	tyrosine-protein phosphatase non-receptor type substrate 1-like	1.38	0.0139
Ppfia2	protein tyrosine phosphatase, receptor type, f polypeptide (PTPRF), interacting protein (liprin), alpha 2	1.38	0.0076
Tslp	ENCODES a protein that exhibits cytokine activity (ortholog)	1.38	0.0205
LOC102555587	uncharacterized LOC102555587	1.38	0.0285
Kctd18	potassium channel tetramerization domain containing 18	1.38	0.0041
LOC684819	ENCODES a protein that exhibits DNA binding (inferred)	1.37	0.0022
Fastk	Fas-activated serine/threonine kinase	1.37	0.0056
Smc2	structural maintenance of chromosomes 2	1.37	0.0121
Prickle3	prickle homolog 3	1.37	0.0447
Zfp706	zinc finger protein 706	1.37	0.0247
Amz1	archaelysin family metallopeptidase 1	1.37	0.0117
Epx	eosinophil peroxidase	1.37	0.0036
Olr302	olfactory receptor 302	1.37	0.0011
Ell	elongation factor RNA polymerase II	1.37	0.0440
Adamts9	ADAM metallopeptidase with thrombospondin type 1 motif, 9	1.37	0.0150
Rad9b	RAD9 checkpoint clamp component B	1.37	0.0496
LOC684932	similar to Zinc finger protein 75	1.37	0.0484
Ggt5	ENCODES a protein that exhibits gamma-glutamyltransferase activity (ortholog)	1.37	0.0299
Jmy	junction-mediating and regulatory protein	1.37	0.0039
Csf1	colony stimulating factor 1 (macrophage)	1.37	0.0019
Fv1	Friend virus susceptibility 1	1.37	0.0225
Ampd1	adenosine monophosphate deaminase 1	1.37	0.0181
Ufm1	ubiquitin-fold modifier 1	1.37	0.0363
Cetn3	centrin, EF-hand protein, 3	1.36	0.0163
Rims3	regulating synaptic membrane exocytosis 3	1.36	0.0416
Gng4	guanine nucleotide binding protein (G protein), gamma 4	1.36	0.0126
Ndufa1	NADH dehydrogenase (ubiquinone) 1 alpha subcomplex, 1	1.36	0.0054
LOC300308	similar to hypothetical protein 4930509O22	1.36	0.0265

Il1rapl1	interleukin 1 receptor accessory protein-like 1	1.36	0.0410
Fbxo25	F-box protein 25	1.36	0.0466
Zmym6	zinc finger, MYM-type 6	1.36	0.0175
---	Uncharacterized protein	1.36	0.0187
Chac1	ChaC glutathione-specific gamma-glutamylcyclotransferase 1	1.35	0.0471
Defb21	defensin beta 21	1.35	0.0474
Ankrd33b	ankyrin repeat domain 33B	1.35	0.0135
Tmem147	transmembrane protein 147	1.35	0.0084
Ncor2	nuclear receptor co-repressor 2	1.35	0.0399
Myof	myoferlin	1.35	0.0143
Rpgr	retinitis pigmentosa GTPase regulator	1.35	0.0226
Slc38a2	solute carrier family 38, member 2	1.35	0.0451
Olr1358	olfactory receptor 1358	1.35	0.0486
Klhl33	kelch-like family member 33 [Source:RGD Symbol;Acc:2324282]	1.35	0.0256
RGD1565002	similar to Dehydrogenase/reductase SDR family member 7 precursor (Retinal short-chain dehydrogenase/reductase 4)	1.35	0.0223
Elof1	ELF1 homolog, elongation factor 1	1.35	0.0196
Tmem60	transmembrane protein 60	1.35	0.0251
Fosl2	fos-like antigen 2	1.35	0.0354
Hacd1	3-hydroxyacyl-CoA dehydratase 1	1.35	0.0068
Mboat2	membrane bound O-acyltransferase domain containing 2	1.35	0.0420
Ssh3	slingshot protein phosphatase 3	1.35	0.0255
LOC102551451	zinc finger protein 665-like	1.35	0.0049
Cd244	Cd244 molecule, natural killer cell receptor 2B4	1.35	0.0481
---	---	1.35	0.0402
LOC100909597	WW domain-containing adapter protein with coiled-coil-like	1.35	0.0037
Olr365	olfactory receptor 365	1.35	0.0260
Rin3	Ras and Rab interactor 3	1.35	0.0183
Xpnpep3	X-prolyl aminopeptidase 3, mitochondrial	1.34	0.0190
Ppfibp1	PTPRF interacting protein, binding protein 1 (liprin beta 1)	1.34	0.0489
RGD1561114	similar to hypothetical protein 4930474N05	1.34	0.0175
Skint10	selection and upkeep of intraepithelial T cells 10 [Source:RGD Symbol;Acc:1587504]	1.34	0.0379
LOC689757	similar to osteoclast inhibitory lectin	1.34	0.0444
Pkia	protein kinase (cAMP-dependent, catalytic) inhibitor alpha	1.34	0.0230
Olr1691	olfactory receptor 1691	1.34	0.0378
Lpar1	lysophosphatidic acid receptor 1	1.34	0.0327
Acot8	acyl-CoA thioesterase 8	1.34	0.0201
Adrm1	adhesion regulating molecule 1	1.34	0.0431
MGC112715	hypothetical protein LOC690899	1.34	0.0283
Pnisr	PNN-interacting serine/arginine-rich protein	1.34	0.0390
Il6st	interleukin 6 signal transducer	1.34	0.0138
Il7	interleukin 7	1.34	0.0122
Olr844	olfactory receptor 844	1.34	0.0021
Olr964	olfactory receptor 964	1.34	0.0356
Olr964	olfactory receptor 964 (Olr964), mRNA	1.34	0.0356
Nudt1	nudix (nucleoside diphosphate linked moiety X)-type motif 1	1.34	0.0296
Meox1	mesenchyme homeobox 1	1.34	0.0480
---	Uncharacterized protein	1.34	0.0365
LOC100364062	M2 pyruvate kinase-like	1.34	0.0215
Rybp	RING1 and YY1 binding protein	1.34	0.0153
Zfp830	zinc finger protein 830	1.34	0.0404
LOC688442	similar to limkain b1	1.34	0.0167
LOC500077	similar to RIKEN cDNA 3110062M04	1.34	0.0159

Tas2r138	taste receptor, type 2, member 138	1.34	0.0090
Fam151b	family with sequence similarity 151, member B	1.33	0.0328
LOC689959	hypothetical protein LOC689959	1.33	0.0043
Arfgap1	ADP-ribosylation factor GTPase activating protein 1	1.33	0.0305
Rabl6	RAB, member RAS oncogene family-like 6	1.33	0.0205
Nfat5	ENCODES a protein that exhibits DNA binding (ortholog)	1.33	0.0018
Gpr152	G protein-coupled receptor 152	1.33	0.0222
Zmym4	zinc finger, MYM-type 4	1.33	0.0199
Tlr5	toll-like receptor 5	1.33	0.0249
Tbx10	T-box 10	1.33	0.0067
Sycp1	synaptonemal complex protein 1	1.33	0.0108
Ap4m1	adaptor-related protein complex 4, mu 1 subunit	1.33	0.0202
NONMMUG002872	Non-coding transcript identified by NONCODE: Linc	1.33	0.0367
Ylpm1	YLP motif containing 1	1.33	0.0407
Mpl	MPL proto-oncogene, thrombopoietin receptor	1.33	0.0365
Dock7	dedicator of cytokinesis 7	1.33	0.0397
Olr1393	olfactory receptor 1393	1.33	0.0344
Dennd4b	DENN/MADD domain containing 4B	1.33	0.0163
Slc25a36l1	INVOLVED IN transport (inferred) AND FOUND IN integral component of membrane (inferred)	1.33	0.0219
Slc22a6	solute carrier family 22 (organic anion transporter), member 6	1.33	0.0230
Trim71	tripartite motif containing 71, E3 ubiquitin protein ligase	1.33	0.0336
Tsfm	Ts translation elongation factor, mitochondrial	1.33	0.0317
Dtx3	deltex 3, E3 ubiquitin ligase	1.33	0.0437
LOC686911	similar to Exocyst complex component 1 (Exocyst complex component Sec3)	1.33	0.0172
Osgin2	oxidative stress induced growth inhibitor family member 2	1.32	0.0474
Fam64a	family with sequence similarity 64, member A	1.32	0.0284
RGD1566369	similar to ribosomal protein S8 [Source:RGD Symbol;Acc:1566369]	1.32	0.0247
Hfm1	HFM1, ATP-dependent DNA helicase homolog	1.32	0.0430
Clcn7	chloride channel, voltage-sensitive 7	1.32	0.0133
Tank	TRAF family member-associated NFKB activator	1.32	0.0093
Plekha1	pleckstrin homology domain containing, family A (phosphoinositide binding specific) member 1	1.32	0.0111
Pkp4	plakophilin 4	1.32	0.0409
Fap	fibroblast activation protein, alpha	1.32	0.0255
Hist1h2aa	histone cluster 1, H2aa	1.32	0.0434
Klc2	kinesin light chain 2	1.32	0.0177
Taf1d	TATA box binding protein (Tbp)-associated factor, RNA polymerase I, D	1.32	0.0298
Nfat5	nuclear factor of activated T-cells 5, tonicity-responsive	1.32	0.0400
Taf4b	TAF4b RNA polymerase II, TATA box binding protein (TBP)-associated factor, 105kDa	1.32	0.0327
Smagp	small cell adhesion glycoprotein	1.32	0.0365
Pex12	peroxisomal biogenesis factor 12	1.32	0.0240
Pfkfb3	6-phosphofructo-2-kinase/fructose-2,6-biphosphatase 3	1.32	0.0272
Dvl1	dishevelled segment polarity protein 1	1.32	0.0054
Tmub1	transmembrane and ubiquitin-like domain containing 1	1.32	0.0281
Ccdc89	coiled-coil domain containing 89 [Source:RGD Symbol;Acc:1305335]	1.32	0.0190
Dcun1d1	DCN1, defective in cullin neddylation 1, domain containing 1 (S. cerevisiae) (Dcun1d1), mRNA. Chalmel, et. al. Testis-expressed Unannotated Transcripts (TUTs)	1.32	0.0428
LOC684545	similar to NACHT, leucine rich repeat and PYD containing 2	1.32	0.0468

Apbb1	amyloid beta (A4) precursor protein-binding, family B, member 1 (Fe65)	1.32	0.0417
Drp2	dystrophin related protein 2	1.32	0.0275
LOC100912377	uncharacterized LOC100912377	1.32	0.0015
Tnrc6c	trinucleotide repeat containing 6C	1.31	0.0062
Nudt2	nudix (nucleoside diphosphate linked moiety X)-type motif 2	1.31	0.0018
Tspy1	testis specific protein, Y-linked 1	1.31	0.0001
Far1	fatty acyl CoA reductase 1	1.31	0.0286
LOC100910996	uncharacterized LOC100910996	1.31	0.0462
Ofd1	oral-facial-digital syndrome 1	1.31	0.0107
Zfp428	zinc finger protein 428	1.31	0.0413
Hoxc8	homeobox C8	1.31	0.0087
Commd1	copper metabolism (Murr1) domain containing 1	1.31	0.0162
Ptger4	prostaglandin E receptor 4 (subtype EP4)	1.31	0.0492
Atmin	ATM interactor	1.31	0.0309
Gtpbp2	GTP binding protein 2	1.31	0.0012
Isy1	ISY1 splicing factor homolog	1.31	0.0487
Cd83	CD83 molecule	1.31	0.0251
Srcap	Snf2-related CREBBP activator protein	1.31	0.0280
Sh2b1	SH2B adaptor protein 1	1.31	0.0371
NONMMUG043962	Non-coding transcript identified by NONCODE: Linc	1.31	0.0162
Abhd17b	abhydrolase domain containing 17B	1.31	0.0381
Nxf1	nuclear RNA export factor 1	1.31	0.0235
Scaf4	SR-related CTD-associated factor 4	1.31	0.0185
Clrn3	clarin 3	-1.31	0.0013
Atxn7l3b	ataxin 7-like 3B	-1.31	0.0096
RGD1562136	similar to D1Ertd622e protein	-1.31	0.0453
LOC100909464	ENCODES a protein that exhibits protein phosphatase type 2A regulator activity (inferred)	-1.31	0.0161
Snx21	---	-1.31	0.0064
Psg29	pregnancy-specific glycoprotein 29	-1.31	0.0288
Tyw3	tRNA-yW synthesizing protein 3 homolog (S. cerevisiae)	-1.31	0.0040
Mtch2	mitochondrial carrier 2	-1.31	0.0161
Nmnat2	nicotinamide nucleotide adenylyltransferase 2	-1.31	0.0455
Ncstn	nicastrin	-1.31	0.0477
Fgf10	fibroblast growth factor 10	-1.31	0.0429
Olr162	olfactory receptor 162	-1.31	0.0412
RGD1561157	RGD1561157	-1.31	0.0080
Sccpdh	saccharopine dehydrogenase (putative)	-1.31	0.0063
Olr1422	olfactory receptor 1422	-1.31	0.0395
Nit2	nitrilase family, member 2	-1.31	0.0000
LOC102554132	uncharacterized LOC102554132	-1.31	0.0489
Tktl1	ENCODES a protein that exhibits catalytic activity (inferred)	-1.31	0.0063
Muc2	mucin 2 [Source:MGI Symbol;Acc:MGI:1339364]	-1.31	0.0068
Fbxo2	F-box protein 2	-1.31	0.0179
Cyb561	cytochrome b-561	-1.31	0.0128
Vom1r79	vomeronasal 1 receptor 79	-1.31	0.0490
Cfap57	cilia and flagella associated protein 57	-1.31	0.0320
Prrt1	proline-rich transmembrane protein 1	-1.31	0.0067
Ddx52	DEAD (Asp-Glu-Ala-Asp) box polypeptide 52	-1.31	0.0500
Trim67	tripartite motif-containing 67	-1.31	0.0255
Map7d3	MAP7 domain containing 3	-1.31	0.0308
Stoml1	stomatin (EPB72)-like 1	-1.31	0.0173
Klhl31	kelch-like family member 31	-1.31	0.0287

Acer2	alkaline ceramidase 2	-1.31	0.0297
Bpgm	2,3-bisphosphoglycerate mutase	-1.32	0.0291
Sgcg	sarcoglycan, gamma	-1.32	0.0048
Jph4	junctophilin 4	-1.32	0.0031
Cd59	CD59 molecule, complement regulatory protein	-1.32	0.0170
---	Uncharacterized protein	-1.32	0.0221
LOC100909612	mitochondrial brown fat uncoupling protein 1-like	-1.32	0.0419
Olr807	olfactory receptor 807	-1.32	0.0209
Cthrc1	collagen triple helix repeat containing 1	-1.32	0.0261
Adam10	ADAM metallopeptidase domain 10	-1.32	0.0334
Apobec4	apolipoprotein B mRNA editing enzyme, catalytic polypeptide-like 4 (putative)	-1.32	0.0041
Bmpr2	bone morphogenetic protein receptor, type II (serine/threonine kinase)	-1.32	0.0212
Rangap1	RAN GTPase activating protein 1	-1.32	0.0398
Wnt2	Protein Wnt	-1.32	0.0220
Taar7d	trace-amine-associated receptor 7d	-1.32	0.0348
Fam167b	family with sequence similarity 167, member B	-1.32	0.0289
Rps21	ribosomal protein S21	-1.32	0.0245
Rab5a	RAB5A, member RAS oncogene family	-1.32	0.0275
Tmc3	transmembrane channel-like 3	-1.32	0.0482
Gng3	guanine nucleotide binding protein (G protein), gamma 3	-1.32	0.0315
Olr1306	olfactory receptor 1306	-1.33	0.0461
Kcng3	potassium channel, voltage gated modifier subfamily G, member 3	-1.33	0.0024
Sft2d2	SFT2 domain containing 2	-1.33	0.0050
Ankk1	ankyrin repeat and kinase domain containing 1	-1.33	0.0040
LOC100361944	hypothetical protein LOC100361944 [Source:RGD Symbol;Acc:2319136]	-1.33	0.0355
Slc30a6	solute carrier family 30 (zinc transporter), member 6	-1.33	0.0308
Tmem178b	transmembrane protein 178B	-1.33	0.0052
P4ha3	prolyl 4-hydroxylase, alpha polypeptide III	-1.33	0.0065
Ednrb	endothelin receptor type B	-1.34	0.0096
Fam91a1	family with sequence similarity 91, member A1	-1.34	0.0152
Xrcc6	X-ray repair complementing defective repair in Chinese hamster cells 6	-1.34	0.0194
Hyi	hydroxypyruvate isomerase	-1.34	0.0181
Tcrb	T-cell receptor beta chain	-1.34	0.0477
Slc23a1	solute carrier family 23 (ascorbic acid transporter), member 1	-1.34	0.0408
Ikzf2	IKAROS family zinc finger 2	-1.34	0.0400
Sh2d6	SH2 domain containing 6 [Source:RGD Symbol;Acc:1587427]	-1.34	0.0480
Tom1	target of myb1 membrane trafficking protein	-1.34	0.0228
LOC100911937	ENCODES a protein that exhibits G-protein coupled receptor activity (inferred)	-1.34	0.0372
LOC102549389	sarcoplasmic/endoplasmic reticulum calcium ATPase 3-like	-1.34	0.0110
Tmem158	transmembrane protein 158	-1.34	0.0496
Slc8a3	solute carrier family 8 (sodium/calcium exchanger), member 3	-1.34	0.0373
Ado	2-aminoethanethiol (cysteamine) dioxygenase	-1.34	0.0292
Ppp1r14d	protein phosphatase 1, regulatory (inhibitor) subunit 14D	-1.34	0.0004
Serpinb9	serpin peptidase inhibitor, clade B (ovalbumin), member 9	-1.34	0.0102
Krt78	Protein Krt78	-1.34	0.0460
Thg1l	tRNA-histidine guanylyltransferase 1-like	-1.34	0.0453
Gsta5	glutathione S-transferase alpha 5	-1.34	0.0124
LOC100360477	ENCODES a protein that exhibits hydrolase activity (inferred) AND isopentenyl-diphosphate delta-isomerase activity (inferred)	-1.35	0.0238
Ppox	protoporphyrinogen oxidase	-1.35	0.0425

Olr91	olfactory receptor 91	-1.35	0.0079
Zfp330	zinc finger protein 330	-1.35	0.0010
Slc35b4	solute carrier family 35 (UDP-xylose/UDP-N-acetylglucosamine transporter), member B4	-1.35	0.0446
Ric3	RIC3 acetylcholine receptor chaperone	-1.35	0.0339
Cps1	carbamoyl-phosphate synthetase 1	-1.35	0.0420
Lcmt1	leucine carboxyl methyltransferase 1	-1.35	0.0411
Lims1	LIM and senescent cell antigen-like domains 1	-1.35	0.0061
Egflam	EGF-like, fibronectin type III and laminin G domains	-1.35	0.0274
C1qtnf7	C1q and tumor necrosis factor related protein 7	-1.35	0.0278
Pigs	phosphatidylinositol glycan anchor biosynthesis, class S	-1.35	0.0417
Enpp1	ectonucleotide pyrophosphatase/phosphodiesterase 1	-1.35	0.0287
Tox	thymocyte selection-associated high mobility group box	-1.35	0.0350
Vom1r13	vomeronasal 1 receptor 13	-1.36	0.0155
Gnat2	guanine nucleotide binding protein (G protein), alpha transducing activity polypeptide 2	-1.36	0.0338
Wbscr16	Williams-Beuren syndrome chromosome region 16	-1.36	0.0278
Fbxo3	F-box protein 3	-1.36	0.0061
Dyrk4	dual-specificity tyrosine-(Y)-phosphorylation regulated kinase 4	-1.36	0.0394
Dbt	dihydrolipoamide branched chain transacylase E2	-1.36	0.0076
Csmd3	CUB and Sushi multiple domains 3	-1.36	0.0356
RGD1565183	ENCODES a protein that exhibits structural constituent of ribosome (inferred) AND INVOLVED IN translation (inferred) AND FOUND IN ribosome (inferred) AND INTERACTS WITH C60 fullerene	-1.36	0.0251
Slitrk6	SLIT and NTRK-like family, member 6	-1.36	0.0276
Lpgat1	lysophosphatidylglycerol acyltransferase 1	-1.36	0.0171
Lama2	laminin, alpha 2	-1.36	0.0228
Uox	urate oxidase	-1.36	0.0174
Syce1	synaptonemal complex central element protein 1	-1.36	0.0158
RGD1560334	similar to Myosin light chain 1 slow a [Source:RGD Symbol;Acc:1560334]	-1.36	0.0200
Uhmk1	U2AF homology motif (UHM) kinase 1	-1.36	0.0365
Il20rb	interleukin 20 receptor beta	-1.36	0.0046
Vom2r44	vomeronasal 2 receptor 44	-1.36	0.0338
Tulp3	tubby-like protein 3	-1.36	0.0053
Padi3	peptidyl arginine deiminase, type III	-1.36	0.0303
Ndufs8	NADH dehydrogenase (ubiquinone) Fe-S protein 8	-1.36	0.0381
LOC685700	hypothetical protein LOC685700	-1.37	0.0116
Ppm1j	protein phosphatase, Mg2+/Mn2+ dependent, 1J	-1.37	0.0255
LOC103692040	olfactory receptor 8B3-like	-1.37	0.0355
Olr1239	ENCODES a protein that exhibits olfactory receptor activity (inferred)	-1.37	0.0355
Olr1239	olfactory receptor 1239	-1.37	0.0355
Stoml2	stomatin (Epb7.2)-like 2	-1.37	0.0264
LOC102552923	olfactory receptor 8B3-like	-1.37	0.0358
Aplp1	amyloid beta (A4) precursor-like protein 1	-1.37	0.0119
Colq	collagen-like tail subunit (single strand of homotrimer) of asymmetric acetylcholinesterase	-1.37	0.0133
Lzts2	leucine zipper, putative tumor suppressor 2	-1.37	0.0144
Olr812	olfactory receptor 812	-1.37	0.0478
Il23r	interleukin 23 receptor	-1.37	0.0441
Avpr2	arginine vasopressin receptor 2	-1.37	0.0121
Rab5c	RAB5C, member RAS oncogene family	-1.37	0.0447
Vwde	von Willebrand factor D and EGF domains [Source:RGD Symbol;Acc:1561223]	-1.37	0.0257

Olr332	olfactory receptor 332	-1.37	0.0210
Usp11	ubiquitin specific peptidase 11	-1.38	0.0418
Olr851	olfactory receptor 851	-1.38	0.0042
Lamtor5	late endosomal/lysosomal adaptor, MAPK and MTOR activator 5	-1.38	0.0331
Erbb4	erb-b2 receptor tyrosine kinase 4	-1.38	0.0389
Ociad2	OCIA domain containing 2	-1.38	0.0074
Si	sucrase-isomaltase (alpha-glucosidase)	-1.38	0.0410
Npsr1	neuropeptide S receptor 1	-1.38	0.0138
LOC691418	hypothetical protein LOC691418	-1.38	0.0485
Dkk2	dickkopf WNT signaling pathway inhibitor 2	-1.38	0.0050
LOC102552106	paired immunoglobulin-like type 2 receptor beta-like	-1.38	0.0401
Pex3	peroxisomal biogenesis factor 3	-1.38	0.0224
Mrpl16	mitochondrial ribosomal protein L16	-1.38	0.0151
LOC291871	similar to heat shock protein 1 beta	-1.38	0.0152
Tdh	L-threonine dehydrogenase	-1.38	0.0185
Arsg	arylsulfatase G	-1.38	0.0431
Zfp787	zinc finger protein 787	-1.38	0.0476
Olr1107	olfactory receptor 1107	-1.38	0.0163
Alpp	alkaline phosphatase, placental	-1.38	0.0140
Irgq	immunity-related GTPase family, Q	-1.38	0.0455
Gabra4	gamma-aminobutyric acid (GABA) A receptor, alpha 4	-1.38	0.0364
Sostdc1	sclerostin domain containing 1	-1.39	0.0319
LOC685463	similar to Ral guanine nucleotide dissociation stimulator (RalGEF) (RalGDS)	-1.39	0.0373
Prl3d4	prolactin family 3, subfamily d, member 4	-1.39	0.0127
Cyb5r3	cytochrome b5 reductase 3	-1.39	0.0272
Krtap13-1	keratin associated protein 13-1	-1.39	0.0065
Psd2	pleckstrin and Sec7 domain containing 2	-1.39	0.0204
Wfdc13	WAP four-disulfide core domain 13	-1.39	0.0183
Fasn	fatty acid synthase	-1.39	0.0381
Pga5	pepsinogen 5, group I (Pga5), mRNA	-1.39	0.0401
Cmbl	carboxymethylenebutenolidase homolog (Pseudomonas)	-1.39	0.0295
Acy1	aminoacylase 1	-1.39	0.0321
Sult1b1	sulfotransferase family, cytosolic, 1B, member 1	-1.39	0.0158
Rnase1l1	ribonuclease, RNase A family, 1-like 1 (pancreatic)	-1.39	0.0186
Tex13	testis expressed gene 13	-1.39	0.0439
Olr1246	olfactory receptor 1246 [Source:RGD Symbol;Acc:1334003]	-1.39	0.0274
Olr1246	olfactory receptor 1246	-1.39	0.0274
Dhcr24	24-dehydrocholesterol reductase	-1.39	0.0284
Stk33	serine/threonine kinase 33	-1.39	0.0324
St6galnac3	ST6 (alpha-N-acetyl-neuraminyl-2,3-beta-galactosyl-1,3)-N-acetylgalactosaminide alpha-2,6-sialyltransferase 3	-1.39	0.0042
Pgbd5	piggyBac transposable element derived 5	-1.39	0.0073
Lix1l	Lix1-like	-1.39	0.0341
Zfp108	zinc finger protein 108 (Zfp108), mRNA.	-1.39	0.0429
Eva1a	eva-1 homolog A	-1.40	0.0320
Olr247	olfactory receptor 247	-1.40	0.0436
Tmem181	transmembrane protein 181	-1.40	0.0364
Iars2	isoleucyl-tRNA synthetase 2, mitochondrial [Source:RGD Symbol;Acc:1311857]	-1.40	0.0157
Heatr1	HEAT repeat containing 1	-1.40	0.0268
Foxa3	forkhead box A3	-1.40	0.0064
Slc44a4	solute carrier family 44, member 4	-1.40	0.0118
Tbxas1	thromboxane A synthase 1, platelet	-1.40	0.0341

Rec114	REC114 meiotic recombination protein	-1.40	0.0237
Zc2hc1b	zinc finger, C2HC-type containing 1B	-1.40	0.0074
Prss12	protease, serine, 12 neurotrypsin (motopsin)	-1.40	0.0140
RGD1559588	similar to cell surface receptor FDFACT [Source:RGD Symbol;Acc:1559588]	-1.40	0.0016
Gp2	glycoprotein 2 (zymogen granule membrane)	-1.40	0.0177
Ap2a2	adaptor-related protein complex 2, alpha 2 subunit	-1.40	0.0117
Lrrn1	leucine rich repeat neuronal 1	-1.40	0.0290
Cd93	CD93 molecule	-1.40	0.0061
LOC102547532	paired immunoglobulin-like type 2 receptor beta-like	-1.40	0.0322
Ano3	anoctamin 3	-1.41	0.0074
Mfsd7	major facilitator superfamily domain containing 7	-1.41	0.0032
Rnase2	ribonuclease, RNase A family, 2 (liver, eosinophil-derived neurotoxin)	-1.41	0.0379
Bles03	basophilic leukemia expressed protein BLES03	-1.41	0.0310
RGD1559683	similar to RIKEN cDNA 1700001C02	-1.41	0.0240
LOC100910256	INTERACTS WITH crocidolite asbestos (ortholog)	-1.41	0.0277
Olr1275	ENCODES a protein that exhibits olfactory receptor activity (inferred)	-1.41	0.0277
LOC100910445	INTERACTS WITH crocidolite asbestos (ortholog)	-1.41	0.0277
Gsta2	ENCODES a protein that exhibits drug binding AND glutathione binding AND glutathione transferase activity	-1.41	0.0048
Olr397	olfactory receptor 397	-1.41	0.0091
Vgll1	INTERACTS WITH diuron AND 17alpha-ethynylestradiol (ortholog) AND 2 3 7 8-tetrachlorodibenzodioxine (ortholog)	-1.42	0.0476
Six6os1	Six6 opposite strand transcript 1	-1.42	0.0220
Agr3	anterior gradient 3, protein disulphide isomerase family member	-1.42	0.0359
Efna4	ephrin A4	-1.42	0.0008
Ttyh2	tweety family member 2	-1.42	0.0285
Barx1	BARX homeobox 1	-1.43	0.0030
Idh3B	isocitrate dehydrogenase 3 (NAD+) beta	-1.43	0.0402
Nme3	NME/NM23 nucleoside diphosphate kinase 3	-1.43	0.0146
Trmt2a	tRNA methyltransferase 2 homolog A	-1.43	0.0204
RGD1565054	similar to 60S acidic ribosomal protein P1	-1.43	0.0042
Adam28	ADAM metallopeptidase domain 28	-1.43	0.0113
Rps29	ribosomal protein S29	-1.43	0.0208
Rrs1	ribosome biogenesis regulator homolog	-1.43	0.0141
LOC688702	similar to RIKEN cDNA 1700001F22	-1.43	0.0009
Pus1	pseudouridylate synthase 1	-1.43	0.0179
Ccdc110	coiled-coil domain containing 110	-1.43	0.0245
Tst	thiosulfate sulfurtransferase	-1.43	0.0248
Cyp2a3	cytochrome P450, family 2, subfamily a, polypeptide 3	-1.44	0.0029
Olr409	olfactory receptor 409	-1.44	0.0236
Olr1602	olfactory receptor 1602	-1.44	0.0073
LOC100910335	INTERACTS WITH crocidolite asbestos (ortholog)	-1.44	0.0153
Olr1275	olfactory receptor 1275	-1.44	0.0153
Stx19	syntaxin 19	-1.44	0.0360
Sigirr	single immunoglobulin and toll-interleukin 1 receptor (TIR) domain	-1.44	0.0037
Ppp2r3c	protein phosphatase 2, regulatory subunit B, gamma	-1.44	0.0417
Abhd11	abhydrolase domain containing 11	-1.44	0.0316
Hapln1	hyaluronan and proteoglycan link protein 1	-1.44	0.0220
St8sia6	ST8 alpha-N-acetyl-neuraminide alpha-2,8-sialyltransferase 6	-1.44	0.0153
Rps29	ribosomal protein S29 [Source:RGD Symbol;Acc:3596]	-1.44	0.0192
Plbd1	phospholipase B domain containing 1	-1.45	0.0384
l7Rn6	lethal, Chr 7, Rinchik 6	-1.45	0.0121
Olr1622	olfactory receptor 1622	-1.45	0.0058

Olr727	olfactory receptor 727	-1.45	0.0492
LOC100910833	histone-lysine N-methyltransferase setd3-like	-1.45	0.0033
Gstm7	glutathione S-transferase, mu 7	-1.45	0.0141
Olr1701	olfactory receptor 1701	-1.45	0.0178
Mmp10	matrix metallopeptidase 10	-1.45	0.0461
Plpp2	phospholipid phosphatase 2	-1.45	0.0354
Ndufa8	NADH dehydrogenase (ubiquinone) 1 alpha subcomplex, 8	-1.45	0.0280
Fam161b	family with sequence similarity 161, member B [Source:RGD Symbol;Acc:1309058]	-1.45	0.0126
Gys1	glycogen synthase 1, muscle	-1.45	0.0156
LOC100912165	rho guanine nucleotide exchange factor 9-like	-1.45	0.0451
LOC102552920	armadillo repeat-containing X-linked protein 5-like	-1.45	0.0299
Creg1	cellular repressor of E1A-stimulated genes 1	-1.45	0.0086
Tpsg1	tryptase gamma 1	-1.46	0.0246
Stxbp6	syntaxin binding protein 6 (amisyn)	-1.46	0.0192
Lyzl6	lysozyme-like 6	-1.46	0.0304
Haus1	HAUS augmin-like complex, subunit 1 (Haus1), mRNA	-1.46	0.0021
LOC100912361	16.5 kDa submandibular gland glycoprotein-like	-1.46	0.0396
Ubxn8	UBX domain protein 8	-1.46	0.0251
Olr1148	olfactory receptor 1148	-1.46	0.0459
Prcp	prolylcarboxypeptidase (angiotensinase C)	-1.47	0.0318
Reep6	receptor accessory protein 6	-1.47	0.0177
LOC100363116	INTERACTS WITH copper(2+) sulfate (ortholog) AND valproic acid (ortholog)	-1.47	0.0237
Olr1142	olfactory receptor 1142	-1.47	0.0015
Elp5	elongator acetyltransferase complex subunit 5	-1.48	0.0225
Olr1368	olfactory receptor 1368	-1.48	0.0002
Far2	fatty acyl CoA reductase 2	-1.48	0.0148
Siva1	SIVA1, apoptosis-inducing factor	-1.48	0.0136
Desi1	desumoylating isopeptidase 1	-1.48	0.0177
Fut11	fucosyltransferase 11 (alpha (1,3) fucosyltransferase)	-1.49	0.0283
Rps29	ribosomal protein S29 [Source:RGD Symbol;Acc:3596]	-1.49	0.0353
C1qtnf3	C1q and tumor necrosis factor related protein 3	-1.49	0.0081
Zfp133	zinc finger protein 133	-1.49	0.0075
Bche	butyrylcholinesterase	-1.49	0.0096
E2f7	E2F transcription factor 7	-1.50	0.0023
Fbxo43	F-box protein 43	-1.50	0.0092
Olr1251	olfactory receptor 1251	-1.50	0.0051
Trpv6	transient receptor potential cation channel, subfamily V, member 6	-1.50	0.0002
Olr1029	olfactory receptor 1029	-1.50	0.0301
Creg2	cellular repressor of E1A-stimulated genes 2 [Source:RGD Symbol;Acc:1564056]	-1.51	0.0039
Entpd7	ectonucleoside triphosphate diphosphohydrolase 7	-1.51	0.0018
Olr373	olfactory receptor 373	-1.51	0.0156
Cplx2	complexin 2	-1.51	0.0146
Olr1143	olfactory receptor 1143	-1.51	0.0174
Olr77	olfactory receptor 77	-1.52	0.0166
Gpx1	glutathione peroxidase 1	-1.53	0.0496
Apmap	adipocyte plasma membrane associated protein	-1.53	0.0105
Rdh11	retinol dehydrogenase 11 (all-trans/9-cis/11-cis)	-1.53	0.0183
Gpr155	G protein-coupled receptor 155	-1.53	0.0052
Edem1	Protein Edem1	-1.53	0.0106
Cttnbp2nl	CTTNBP2 N-terminal like	-1.54	0.0186
Defb10	defensin beta 10	-1.54	0.0101

Pate3	prostate and testis expressed 3	-1.54	0.0300
Kcnn4	potassium channel, calcium activated intermediate/small conductance subfamily N alpha, member 4	-1.54	0.0123
Coa3	cytochrome C oxidase assembly factor 3	-1.54	0.0497
Rps29	ribosomal protein S29 [Source:RGD Symbol;Acc:3596]	-1.55	0.0259
LOC102553032	ellis-van Creveld syndrome protein homolog	-1.56	0.0167
Lamb3	laminin, beta 3	-1.56	0.0255
Nrip2	nuclear receptor interacting protein 2	-1.56	0.0144
Tas2r140	taste receptor, type 2, member 140	-1.57	0.0036
Ifi27	interferon, alpha-inducible protein 27	-1.57	0.0389
Gstk1	glutathione S-transferase kappa 1	-1.57	0.0248
Zbtb26	zinc finger and BTB domain containing 26	-1.57	0.0359
LOC688842	hypothetical protein LOC688842	-1.58	0.0317
Tm7sf2	transmembrane 7 superfamily member 2	-1.58	0.0402
Adssl1	Adenylosuccinate synthetase isozyme 1	-1.58	0.0152
Thoc6	THO complex 6	-1.58	0.0106
Slc26a7	solute carrier family 26 (anion exchanger), member 7	-1.59	0.0042
Srek1ip1	SREK1-interacting protein 1	-1.60	0.0191
Ankrd39	ankyrin repeat domain 39	-1.60	0.0031
Hyal5	hyaluronoglucosaminidase 5	-1.61	0.0002
Cyp26a1	cytochrome P450, family 26, subfamily a, polypeptide 1	-1.63	0.0087
LOC500300	similar to hypothetical protein MGC6835	-1.64	0.0228
LOC102552988	uncharacterized LOC102552988	-1.64	0.0325
Hes6	hes family bHLH transcription factor 6	-1.65	0.0363
LOC100361139	ENCODES a protein that exhibits actin binding (inferred) AND INVOLVED IN actin cytoskeleton organization (inferred) AND sequestering of actin monomers (inferred) AND FOUND IN cytoplasm (inferred)	-1.65	0.0210
Siglec1	sialic acid binding Ig-like lectin 1, sialoadhesin	-1.66	0.0404
Lrrcc1	leucine rich repeat and coiled-coil centrosomal protein 1	-1.66	0.0287
Ppp1r3b	protein phosphatase 1, regulatory subunit 3B	-1.68	0.0323
RGD1559459	similar to Expressed sequence AI788959	-1.69	0.0116
Hebp2	heme binding protein 2	-1.71	0.0283
Enpp5	ectonucleotide pyrophosphatase/phosphodiesterase 5	-1.72	0.0120
Map3k15	mitogen-activated protein kinase kinase kinase 15 [Source:RGD Symbol;Acc:1560603]	-1.73	0.0174
Gstt3	glutathione S-transferase, theta 3	-1.74	0.0093
Aqp11	aquaporin 11	-1.76	0.0114
Ces4a	carboxylesterase 4A	-1.78	0.0082
Vom2r75	vomeronasal 2 receptor, 75	-1.86	0.0040
Trim24	transcription intermediary factor 1-alpha [Source:RefSeq peptide;Acc:NP_001037731]	-1.98	0.0428
Angptl8	angiopoietin-like 8	-2.19	0.0160

Table S4 qPCR validation of microarray data for selected differentially expressed transcripts (FC > 1.3 or < -1.3, *P* < 0.05) hepatic transcripts between the groups OC vs. LC.

	FC OC vs. LC		*P*-value OC vs. LC	
**Gene symbol**	**Microarray**	**qPCR**	**Microarray**	**qPCR**
G6pd	21.80	5.56	<0.0001	<0.0001
Scd2	10.50	8.89	0.0053	0.0005
Elovl6	9.49	7.17	0.0032	0.0001
Gpam	8.64	7.77	<0.0001	0.0001
Cd36	8.42	11.93	<0.0001	0.0037
Me1	6.61	4.08	0.0006	0.0001
Fasn	3.64	2.19	0.0051	0.0093
Srebf1	2.36	1.28	0.0003	0.5101
Fads2	2.07	1.64	<0.0001	0.0327
Ldlr	1.63	1.27	0.0268	0.2104
Elovl5	1.62	1.52	0.0013	0.0220
Car3	-13.50	-7.74	0.0001	0.0050
Dhrs7	-14.98	-4.79	<0.0001	0.0003
Sult1c3	-16.33	-6.34	0.0001	<0.0001
Nrep	-16.95	-7.52	<0.0001	0.0033
Cyp3a18	-20.66	-7.81	<0.0001	<0.0001
Sds	-22.07	-27.00	0.0108	0.0377
Cdh17	-59.12	-71.54	<0.0001	0.0054
Acnat2	-190.34	-301.0	<0.0001	0.0008

The microarray FC was calculated from n = 6 microarrays/group. The qPCR FC was calculated from n = 8 samples/group.

Table S5 Characteristics of gene-specific primers used for qPCR analysis

Gene symbol	Primer sequence	NCBI GenBank	Annealing T	Product size (bp)
Reference genes				
Actb	GACCTCTATGCCAACACAGT<br>CACCAATCCACACAGAGTAC	NM_031144	60	154
Atp5b	GCACCGTCAGAACTATTGCT<br>GAATTCAGGAGCCTCAGCAT	NM_134364	57	203
Canx	CCAGATGCAGATCTGAAGAC<br>CTGGGTCCTCAATTTCACGT	NM_172008	60	175
Target genes				
Acnat2	ACTTGCTCACCCCAAGATCC<br>CCCCTGACCCATGAAATTGGA	NM_001014063	60	221
Car3	GGCCCTCTCTCTGGACCCTA<br>AATCCCATCGGGCTGCTTCA	NM_019292	60	177
Cdh17	AGCAAGTATCTCCACGGCTG<br>GCCATATGCGCCAGTCAAAT	NM_053977	60	152
Cd36	GCTGATTACTTCTGTGTAGTAGCTT<br>GCTCCAGTAATGAGCCCACA	NM_031561	60	242
Cyp3a18	CTGTCAGCTGGGAAGGAAACT<br>AAATTTCCACATGCCATCACCG	NM_145782	60	232
Dhrs7	CTAGTCTTGGGGCTGCTGAG<br>TAGGTTGCCATTCTCCAGGC	NM_001013098	60	249
Elovl5	ATGAACTGGGTTCCCTGCGG<br>GGAAGGGACAGAGGACAGGC	NM_134382	60	102
Elovl6	AAGTTTGAACTGCGGAAGCCG<br>CACCTAGTTCGGGTGCTTTGC	NM_134383	60	211
Fads2	CATCGACCGCAAGGTCTACAAC<br>CTTGCCCACGAAATCCAGGTC	NM_031344	60	139
Fasn	AGGTGCTAGAGGCCCTGCTA<br>GTGCACAGACACCTTCCCAT	NM_017332	60	281
Gpam	CAGCGTGATTGCTACCTGAA<br>CTCTCCGTCCTGGTGAGAAG	NM_017274	60	194
G6pd	TTGTACCAGGGTGATGCCTTCC<br>GCTCACTCTGTTTGCGGATGTC	NM_017006	60	199
Ldlr	ACAGTGTCCTCCCAAGTCCAA<br>GCAAATGTGGATCTCGTCCTC	NM_175762	60	222
Me1	CTGCCTTGGGGATTGCTCAT<br>GAGAGAAGCACGCCCCTTA	NM_012600	60	128
Nrep	GGAGGGAGGTCTTACTAAGGGAA<br>GTAACCGATAGCTGGGGAGC	NM_178096	60	130
Scd2	TGCACCCCCAGACACTTGTAA<br>GGATGCATGGAAACGCCATAG	NM_031841	60	94
Sds	CCGGCACTAGTGTGTTCCTT<br>ACAGCCTTGTTTTGCCTTCATC	NM_053962	60	104
Srebf1	TCTTGACCGACATCGAAGACAT<br>CCAGCATAGGGGGCATCAAA	NM_001276707	60	85
Sult1c3	ACCTGTCAAGTCCCCATTCT<br>ATTTCCTGCGTCCAGGTGGT	NM_031732	60	217
